# Pharmacologic and surgical therapies for patients with Meniere’s disease: A systematic review and network meta-analysis

**DOI:** 10.1371/journal.pone.0237523

**Published:** 2020-09-01

**Authors:** Nadera Ahmadzai, Wei Cheng, Shaun Kilty, Leila Esmaeilisaraji, Dianna Wolfe, James Bonaparte, David Schramm, Elizabeth Fitzpatrick, Vincent Lin, Becky Skidmore, Brian Hutton

**Affiliations:** 1 Ottawa Hospital Research Institute, Clinical Epidemiology Program, Ottawa, Canada; 2 Department of Otolaryngology – Head and Neck Surgery, The Ottawa Hospital, Ottawa, Canada; 3 Dr. S. Kilty Medicine Prof. Corp, Ottawa, Canada; 4 The University of Ottawa Faculty of Epidemiology and Community Medicine, Ottawa, Canada; 5 Faculty of Health Sciences, University of Ottawa, Ottawa, Canada; 6 CHEO Research Institute, Ottawa, Canada; 7 Department of Otolaryngology – Head & Neck Surgery, Sunnybrook Health Sciences Centre, Sunnybrook Research Institute, Toronto, Canada; 8 Faculty of Medicine, University of Toronto, Toronto, Canada; Universidad de Antioquia, COLOMBIA

## Abstract

**Background:**

Meniere’s disease (MD) is a chronic condition of the inner ear consisting of symptoms that include vertigo attacks, fluctuating sensorineural hearing loss, tinnitus and aural fullness. Despite availability of various interventions, there is uncertainty surrounding their relative efficacy, thus making it difficult to select the appropriate treatments for MD. The objective of this systematic review was to assess the relative effects of the available pharmacologic and surgical interventions in patients with MD with regard to vertigo and other key patient outcomes based on data from randomized clinical trials (RCTs).

**Methods:**

Our published protocol registered with PROSPERO (CRD42019119129) provides details on eligibility criteria and methods. We searched various databases including MEDLINE, Embase and the Cochrane Library from inception to December 10^th^, 2018. Screening at citation and full-text levels and risk of bias assessment were performed by two independent reviewers in duplicate, with discrepancies resolved by consensus or third-party adjudication. Bayesian network meta-analyses (NMA) were performed for hearing change and vertigo control outcomes, along with pairwise meta-analyses for these and additional outcomes.

**Results:**

We identified 2,889 unique citations, that yielded 23 relevant publications describing 18 unique RCTs (n = 1,231 patients). Overall, risk-of bias appraisal suggested the evidence base to be at unclear or high risk of bias. Amongst pharmacologics, we constructed treatment networks of five intervention groups that included placebo, intratympanic (IT) gentamicin, oral high-dose betahistine, IT steroid and IT steroid plus high-dose betahistine for NMAs of hearing change (improvement or deterioration) and complete vertigo control. IT steroid plus high-dose betahistine was associated with the largest difference in hearing improvement compared to placebo, followed by high-dose betahistine and IT steroid (though 95% credible intervals failed to rule out the possibility of no difference), while IT gentamicin was worse than IT steroid. The NMA of complete vertigo control suggested IT gentamicin was associated with the highest probability of achieving better complete vertigo control compared to placebo, followed by IT steroid plus high-dose betahistine. Only two studies related to surgical interventions were found, and data suggested no statistically significant difference in hearing changes between endolymphatic duct blockage (EDB) versus endolymphatic sac decompression (ESD), and ESD with or without steroid injection. One trial reported that 96.5% of patients in EDB group compared to 37.5% of the patients in ESD group achieved complete vertigo control 24 months after surgery (p = 0.002).

**Conclusion:**

To achieve both hearing preservation and vertigo control, the best treatment option among the pharmacologic interventions compared may be IT steroid plus high-dose betahistine, considering that IT gentamicin may have good performance to control vertigo but may be detrimental to hearing preservation with high cumulative dosage and short interval between injections. However, IT steroid plus high-dose betahistine has not been compared in head-to-head trials against other interventions except for IT steroid alone in one trial, thus future trials that compare it with other interventions will help establish comparative effectiveness with direct evidence.

## Background

Hearing loss, one of the leading causes of disability in Canada and worldwide, affects more than one million Canadians [1, 2]. In spite of various available interventions, Canadians with hearing loss may experience severely reduced quality of life [3]. Meniere’s disease (MD), a condition that causes hearing loss, has a variable clinical course [4] and often an underestimated emotional and financial toll on patients, their families and society [5]. MD consists of a triad of symptoms, including fluctuating sensorineural hearing loss (SNHL), vertigo attacks, and tinnitus [6]. Quality of life can be dramatically impacted due to reduction in social participation, physical activity, increased fatigue, and diminished work capacity. These restrictions on life, the persistent uncertainty of vertigo, the underlying hearing loss, and tinnitus [6, 7] can cause anxiety and other psychological disorders, with 40–60% of individuals with intractable MD experiencing neuroses and/or depression [8]. An increase in the hydraulic pressure and endolymphatic volume within the inner ear system, termed “endolymphatic hydrops” (EH), is associated with MD symptoms. MD is diagnosed based on symptoms, with guidelines that define diagnostic criteria and certainty [9]. Both medical and surgical interventions are used in the management of MD [10] with the aim to treat and prevent vertigo attacks, improve or preserve hearing and vestibular function, and prevent bilateral MD [11]. Medical interventions, systemic (e.g., diuretics, antihistamines) and intratympanic therapies (e.g., gentamicin, steroids) focus on either reducing pressure in the endolymphatic system or chemical labyrinthectomy [10, 11]. Dietary therapies including restrictions of salt, water, alcohol, and caffeine, are used to reduce endolymphatic pressure [11]. Surgical treatments include ablative labyrinthectomy, after which hearing is lost, and more conservative procedures in which hearing preservation is attempted [11]. The results of any therapy may be improved through adjunctive psychological support [8].

Early detection of MD and rapid intervention are important to reduce the damaging effects of MD on patients’ quality of life [7]. In addition to patients suffering with MD, other stakeholders will benefit from this work, including clinicians and family members of patients with MD. Clinicians were actively involved throughout the review process including question formulation, development of eligibility criteria, provision of clinical expertise at various steps of the review and interpretation of findings.

Despite the availability of various interventions, there is uncertainty surrounding their comparative efficacy, making it difficult to select the appropriate treatment. We conducted a systematic review incorporating pairwise and network meta-analyses (NMAs) with the objectives to assess the relative effects of the available pharmacologic and surgical interventions in patients with MD with regard to vertigo and other key outcomes.

## Methods

Our review adhered to methods recommended by the Cochrane Collaboration for the conduct of systematic reviews of interventions [12], and conformed to reporting standards of the Preferred Reporting Items for Systematic Reviews and Meta-Analyses for Network Meta-Analyses (PRISMA-NMA) [13]. We registered the review with the International Prospective Register of Systematic Reviews (PROSPERO; registration #CRD42019119129) [14] and published the review protocol [15]. Minor deviations from the planned protocol were encountered during the review and are detailed in S1 Text. A summary of the review methods is presented next. For readers unfamiliar with NMA, overview documents regarding this methodology may prove to be of interest toward gaining additional familiarity in this area [16–20].

### Data sources and search for studies

The search strategies were developed and tested through an iterative process by an experienced medical information specialist in consultation with the review team. The strategies were peer reviewed by another senior information specialist prior to execution using the PRESS Checklist [21]. Using the OVID platform, we searched Ovid MEDLINE^®^, including Epub Ahead of Print and In-Process & Other Non-Indexed Citations, and Embase Classic+Embase. We also searched the Cochrane Library on Wiley. All searches were performed on December 10, 2018.

Strategies utilized a combination of controlled vocabulary (e.g., “Meniere Disease”, “Endolymphatic Hydrops”) and keywords (e.g., “Meniere”, “cochlear hydrops”, “labyrinth syndrome”). Results were filtered using hedges for systematic reviews, randomized controlled trials (RCTs) and non-randomized controlled trials as applicable for each database. Vocabulary and syntax were adjusted across databases. When possible, animal-only and opinion pieces were removed from the results.

A grey literature search of targeted clinical trial registries, ClinicalTrials.gov and the International Clinical Trials Registry Platform, was performed on December 14, 2018. We also searched hearing-related web sites and performed various searches using Google Scholar. The proposed database search strategies are provided in S2 Text.

### Identification of articles

The study’s research question and eligibility criteria with regard to the PICOTS (Population, Intervention, Comparator, Outcomes, Timing, Study design) framework were established a prior and reported in the published protocol [15], and are also detailed in SA1 Table in S3 Text. In brief, a RCT or quasi-RCT was to be included if it compared pharmacological or surgical interventions of interest to placebo or other active arms for treatment of patients with MD defined as per the established criteria (i.e., American Academy of Otolaryngology-Head and Neck Surgery (AAO-HNS) 1985, AAO-HNS 1995, and American Academy of Ophthalmology and Otolaryngology (AAOO) 1972) [9, 22], and reported a minimum follow-up time of six months post-treatment. Studies that used other designs, RCTs that used other MD diagnostic criteria, and studies with less than six months of follow-up time were excluded.

### Screening and data collection process

Screening was performed in two levels involving two reviewers (NA and LE), working independently with the established eligibility criteria via online systematic review software (Distiller Systematic Review (DSR) Software; Evidence Partners Inc, Ottawa, Canada). At Level 1, abstracts and titles of the de-duplicated literature search findings were reviewed, while at Level 2, the full-texts of articles deemed relevant during level 1 were screened. Level 1 screening was performed using the liberal accelerated method [23], in which one reviewer is required to include citations for further assessment at the next level and two reviewers are needed to exclude a citation. Level 2 screening required two reviewers to assess all full-texts independently and in duplicate. Screening at both levels began with a calibration exercise to ensure consistent application of the review eligibility criteria between reviewers. Overall 25 citations were piloted at Level 1 and 25 full-texts were piloted at Level 2. Disagreements among reviewers were resolved through consensus or third-party adjudication. References of all included studies were scanned for inclusion by one reviewer (LE), and potentially relevant records were screened using the above described approach. One content expert (SK) was consulted for additional studies. Where necessary, content experts were consulted, and study authors were contacted to verify eligibility of the studies.

A standardized data extraction form in Microsoft Excel (Microsoft Corporation, Seattle, Washington, USA) was designed and employed. Key data extracted included study traits (e.g. author, publication year), patient characteristics (e.g., inclusion and exclusion criteria, diagnostic criteria for MD, important baseline traits such as initial hearing loss, vertigo, age, and gender), intervention and comparator details (e.g. dose, frequency, unit, duration, strategy, route of administration, co-interventions, surgical approach), outcome information (e.g. number analyzed, number of events, mean, standard deviation), and study design information (e.g. cited trial design, number of sites, duration of follow-up, funding source, and authors’ conflicts of interest). A complete listing of data extraction items is presented in the published protocol [15]. Data extraction was piloted on a set of five studies prior to proceeding with extraction of all included studies. Data were extracted by two reviewers (NA and LE), working independently and in duplicate, except for aggregated data extracted from figures or calculated from individual data (WC). Conflicts were resolved via consensus or third-party adjudication. We contacted study authors for any missing or additional data of interest via email (one initial contact and at least two reminders).

### Risk of bias assessment

The Cochrane Risk of Bias Tool for RCTs [24] was used to evaluate the risk of bias of included RCTs. Two reviewers (NA, LE) carried out assessments independently and in duplicate, and resolved conflicts via consensus or third-party adjudication. All domains of the Cochrane Risk of Bias tool for RCTs were considered, including selection bias (sequence generation, and allocation sequence concealment), performance bias (blinding of participants and personnel), detection bias (blinding of outcome assessment), attrition bias (incomplete outcome data), reporting bias (selective reporting), and other biases considered relevant to the review topic. Where possible, we also considered baseline imbalances between groups with respect to comorbidities and factors that may affect the outcomes to assess selection bias. We assessed outcome-specific risks of bias across all reported outcomes of interest in a trial and assigned an overall rating if the risk of bias did not differ across outcomes. In such studies, an overall high risk of bias was assigned if at least one domain was rated to be at high risk of bias. Similarly, the study was rated at unclear risk of bias if none of the domains was appraised as high risk of bias but at least one domain was evaluated as unclear risk of bias.

### Approach to evidence synthesis

Detailed information related to study methods, patient characteristics, and patient enrollment criteria were collected to assess the transitivity assumption for NMA (i.e. the similarity of studies). These data included the mean age at onset and duration of MD, proportion of female patients, frequency and severity measures of vertigo at baseline, measures of average tinnitus intensity/frequency at baseline, measure of initial pure tone average, MD stage, and other factors. The study features were reviewed with our clinical experts using a combination of table summaries and descriptive plots to identify potential outlier studies that may warrant exclusion from meta-analyses, and to appraise the comparability of characteristics across studies. Network geometry was assessed through the creation of network diagrams and inspection of the patterns of comparisons available for each outcome. Separate comparisons between pharmacologic therapies and between surgical interventions were performed.

Initially, we inspected the characteristics of included studies such as patients’ clinical characteristics (age, sex, and clinical history, duration of MD, baseline severity, enrollment criteria about failure to respond to certain prior interventions, etc), interventions (concentration and dosage, time interval between injections, etc) and methodologic homogeneity (e.g., risk of bias, study design), and we summarized them accordingly. A pairwise meta-analysis for each intervention comparison was pursued to explore statistical heterogeneity, if data permitted. Statistical heterogeneity was assessed using a combination of factors [25], including 1) visual inspection of the degree to which point estimates varied across studies, 2) visual inspection and identification of scenarios wherein confidence intervals (CIs) of study treatment effects of common treatment comparisons showed minimal or no overlap, 3) review of whether the statistical test for heterogeneity showed a low P-value (< 0.10), and 4) noting whether the *I*^*2*^ statistic quantified a large proportion of the variation in point estimates to be due to among-study differences. When there was a high degree of overlap in the 95% CIs of treatment effects and the research team judged the characteristics of study populations and interventions to be similar across studies, we still presented the pooled estimates from meta-analyses despite substantial heterogeneity (I^2^ between 50–90%). When we did not present the meta-analysis of a pairwise comparison due to excessive heterogeneity, the results were presented in a forest plot without a pooled estimate. All pairwise meta-analyses were conducted using Review Manager version 5.3 [26]. If the evidence network of a specified clinical endpoint allowed for NMA and the expert team judged the clinical and methodologic homogeneity of studies to be adequate, both fixed-effect and random-effects (RE) Bayesian NMAs were performed to compare interventions used in the included studies that were sufficiently connected. A common between-trial standard deviation was used for random effects NMA models as per established methods [27–29]. Vague prior distributions were planned for treatment effects (specifically, Normal (0,10000) for the mean difference in hearing change and Normal (0, 100) for the log odds ratio of vertigo control). For the between-study standard deviation parameter in random effects models, a vague prior distribution was used (specifically, Uniform (0, 20) for hearing change and Uniform (0, 5) for vertigo control). We fit random effects unrelated means models to the data and compared deviance information criteria (DIC) values and posterior mean deviance contributions with those from consistency models to detect violations of the consistency assumption. Model fit was assessed by comparing total residual deviance with the number of unconstrained data points [30] and was considered adequate if these quantities were approximately equal. The DIC was used for selection between models, with a difference of five points suggesting an important difference [30], (and smaller values being preferred).

The type of endpoint under analysis (e.g., continuous or binary) determined the use of specific NMA models. We used mean change per arm (between pre- and post-intervention) to analyze continuous endpoints measured in the same units, with mean difference as the corresponding effect size. It was common for studies to report results regarding the same continuous endpoint in different formats. In some cases mean changes with corresponding standard errors (SEs) were presented, while in other cases only mean values at baseline and post-treatment with corresponding standard deviations (SDs) for each treatment arm were reported. For the latter scenario, we considered the appropriateness of assuming a correlation between mean values at baseline and follow-up and calculated the mean changes and corresponding SEs when they were not reported. Estimates of effect sizes for binary endpoints were expressed as odds ratios. NMA estimates of all pairwise comparisons between interventions were expressed with their corresponding 95% credible intervals (CrIs). Secondary measures of effect including the Surface Under the Cumulative RAnking curve (SUCRA) and average treatment rankings [31] were estimated to explore potential orderings of treatments. The comparison-adjusted funnel plots were applied to assess for small-study effects as signals of publication bias [32] All NMAs were carried out using OpenBUGS version 3.2.3 [33] and the R2OpenBUGS package [34] version 3.2–3.2 in R.

If the evidence network for an outcome was not well-connected (e.g., no closed loops among interventions), we reported the results from pairwise meta-analysis. If the research team judged a study to be too different based on clinical characteristics from the remainder of studies reporting a common intervention, we performed the NMA without the study and explored the possible options of sensitivity analysis. We also conducted sensitivity analyses using an alternative correlation between mean values at baseline and follow-up for each outcome. Whenever feasible, we also planned to explore subgroup analyses to evaluate the impact of gender distribution (e.g., percent females), age (older versus younger), body mass index (BMI) (higher BMI versus lower BMI), race (white versus others), presence of dizziness, number of days since initial treatment (or onset of MD), severity of initial hearing loss, type of MD (unilateral versus bilateral), and types of unilateral MD.

## Results

### Extent of available literature

**Fig 1:**
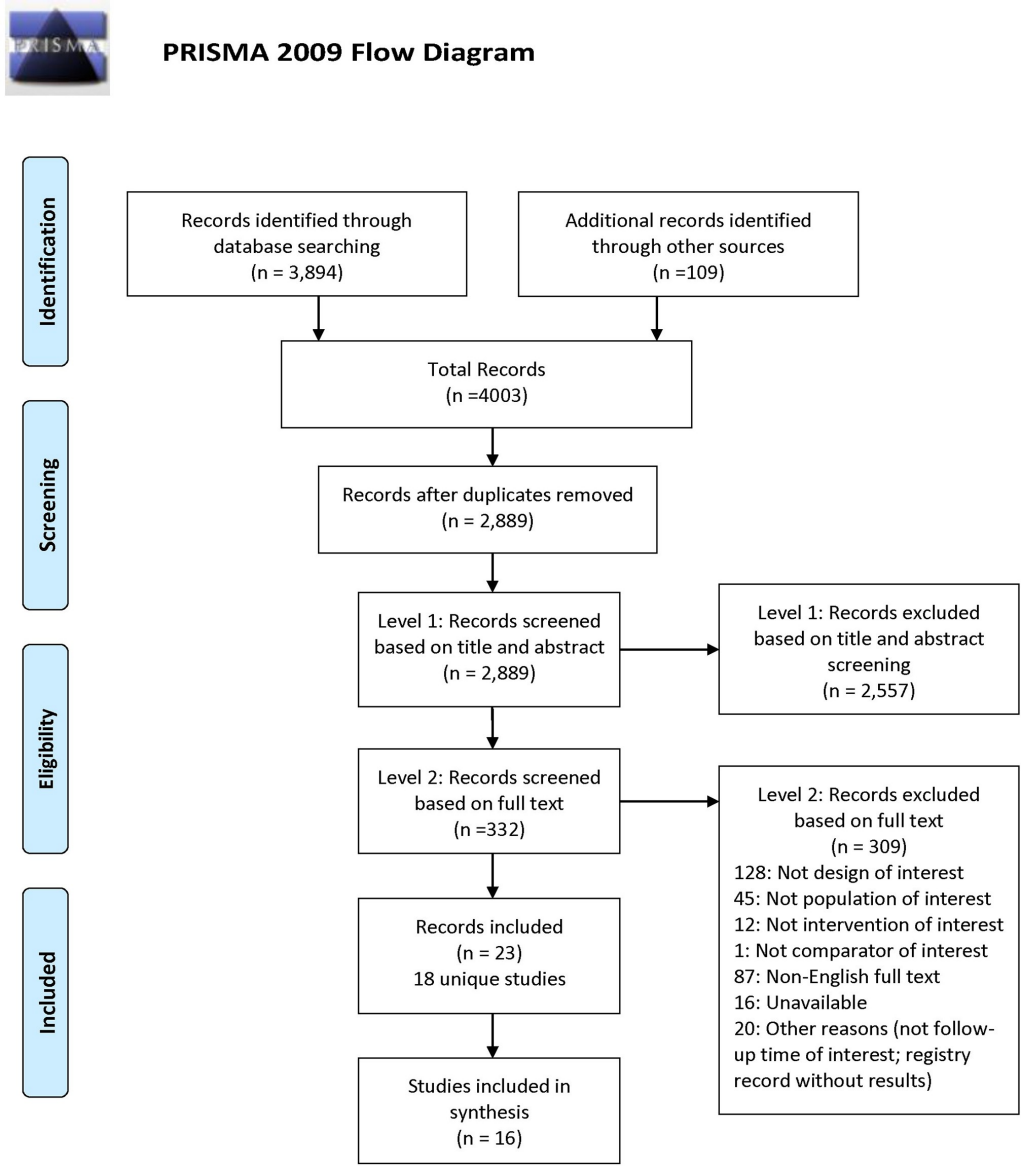
Process of study selection. The flow diagram shown presents details of the process of study selection toward identification of the evidence base for this systematic review.

A total of 4,003 bibliographic records (database searches yield = 3,894; grey literature = 84; reference checking and expert nominated = 25) were identified. Duplicate records were removed, and 2,889 remaining citations were screened at level 1 based on title and abstract. We excluded 2,557 citations at Level 1, and 332 passed to Level 2 for full-text screening. Amongst these, 23 records describing 18 unique studies were included [35–57], with 16 of these studies being quantitatively or narratively analyzed [35, 36, 38–46, 48–51, 57] (Fig 1, PRISMA flow diagram). Two studies were not included in data summaries because one [47] was not deemed a randomized trial, although it was labelled as such, and the other was terminated early before reaching its targeted sample size, resulting in a sample (n = 15 patients in total with 4, 5 and 5 patients per arm) that was considered too small to reach balanced baseline characteristics and support the validity of the results [43]. Further detail on these studies is provided in S3 Text.

Across the 16 studies included for syntheses, the median publication year was 2012 (range: 2004 [50] to 2018 [35]), and the median sample size was 57 (range: 16 [48] to 214 [41]).

Six studies were non-industry funded [35, 36, 38, 39, 41, 47], five declared no funding [40, 42–44, 57], and seven did not report funding source [37, 45, 46, 48–51]. Country of conduct and/or the corresponding authors’ locations varied across the studies, and included the United States [35], Canada [57], Brazil [45], Egypt [51], Germany [41], India [37], Iran [36], Italy [44], Italy and Romania [40, 42], Japan [39, 47], Mexico [48, 49], the Netherlands [43, 46, 50], and the United Kingdom [38].

### Study populations

The included patients were adults diagnosed with MD based on AAO-HNS 1995 [9] in 19 publications of 14 unique studies [35, 38–42, 44–46, 48–57], AAO-HNS 1985 in one study [37] and AAO-HNS with no mention of the year in one study [36]. MD was defined as definite in all studies except in three (suspected in one [45], interactable in one [47], not reported in one [48]). Patients suffered from unilateral MD in 11 studies [35, 36, 38–40, 42, 44, 46, 49–51], a mixture of unilateral and bilateral MD in two studies [41, 57], and laterality (unilateral or bilateral) was unclear in three studies [37, 45, 48]. Type of MD was definite in 14 studies [35–42, 44, 46, 49–51, 57], suspected in one study [45], and was not reported in another study [48]. Studies reported unsuccessful benefit from prior salt restriction and/or conservative medical treatment, including failed medical therapy for three months [35, 36], three to six months [39], at least six months [40, 42, 44, 49–51, 57], and for an unknown duration of time [38, 46]; in three studies, there was no information about prior treatment failure [37, 41, 45], and in one study patients had rejected surgical management and were poorly controlled by maintenance therapy [48]. Further details on the specific prior treatments that patients received are presented in SA2 Table in S3 Text.

A total of 1,020 patients were included in the set of 16 studies, with mean age ranging from 38.5 [37] to 59 years [50]. Thirteen of the 16 studies reported total enrollment of 435 males (ranging from 27% [49] to 65% [37]) and 531 females [36–42, 44, 45, 49–51, 57]; three studies did not report this information [35, 46, 48]. Study populations consisted of adults, except in one trial [37] where the control group (n = 20) ranged in age from 17 to 78 years. Twelve of 13 studies that reported laterality for MD involved 682 unilateral and 49 bilateral patients [35, 36, 38–42, 44, 46, 49–51], while one study did not report this information [37]. Seven studies [36, 38, 39, 44, 45, 48, 51] reported MD duration that ranged from a few months [38] to 17 years [51]. Further details are presented in Table 1.

**Table 1:** Baseline demographic characteristics of the patients in the included studies based on treatment groups (1; 2; 3).

Author (Publication Year)	N	Age in years	Sex	Affected ear	Initial PTA	MD duration in years; (n); mean±SD; median (range)	MD Stage: I, II, II, IV (n)	Vertigo class (n); vertigo frequency / month mean±SD
Treatment Groups		mean±SD; median (range)	(F)	(Rt: Lt)	mean±SD; level (n)			
**Bojrab (2018)** [35]								
**Group 1**: Endolymphatic sac decompression surgery (ESD) + Steroid injection	18	56.7 ± 9.5	NR	NR	45.5 ± 17.4*	NR	NR	3.3±NR
**Group 2**: ESD	17	51.8 ± 10.6	NR	NR	39.9 ±16.5*	NR	NR	3.4±NR
**Kitahara (2016)** [39]								
**Group 1**: Tympanic ventilation tube + medication (including Diuretics, Betahistine, Diphenidol, Dimenhydrinate, and Diazepam)	63	51.7 ± 14.7	41	31:32:00	49.5 ± 13.2**	3.1±1.7	NR	1.8 ±1.1**
**Group 2**: medication (including diuretics, Betahistine, Diphenidol, Dimenhydrinate, and Diazepam)	70	49.7 ± 16.5	45	31:39:00	47.3 ± 13.4**	2.5±1.9	NR	1.6±1**
**Masoumi (2017)** [36]					≤25(20),			A (0), B (27), C (9);
**Group 1**: IT dexamethasone	36	39.9±13.84	15	22;14	26-40(11), 41-70(5);	4.54±1.98	NR	A (0), B (20), C (13)
**Group 2**: IT methylprednisolone	33	41.51±11.68	15	16:17	≤25(15), 26-40(15), 41-70(3)	4.06±3.12	NR	
**Paragache (2005)** [37]								
**Group 1**: IT Dexamethasone	20	38.5 (24–56)	7	NR	NR	NR	NR	NR
**Group 2**: Conventional medical treatment (salt and caffeine restricted diet, and nicotine and alcohol restrictions, cinnarizine 25 mg three times a day for acute episodes, and betahistine 16 mg three times a day for maintenance therapy)	20	40.3 (17–78)	7	NR	NR	NR	NR	NR
**Albu (2015)** [42]							II: 5, III: 20,	
**Group 1**: IT Dexamethasone +placebo pills	30	50.2 ± 14.3	23	NR	57.4 ± 14.7	NR	IV: 8;	8.6±NR
**Group 2**: Betahistine + IT placebo (saline)	29	51.5 ± 14.3	20	NR	58.2 ± 13.9	NR	II: 7, III: 21, IV: 5	7.9±NR
**Patel (2016)** [38]								
**Group 1**: IT Methylprednisolone	30	51·6 ± 10·2	10	13:17	53.3 ±21.2	4.1 ±3.2	NR	5.5±6.5
**Group 2**: IT Gentamicin	30	53·3 ± 10·8	15	12:18	51.5 ±11.3	4.9 ±5.6	NR	6.9±16.7
**Casani (2012)** [44]								
**Group 1**: IT Dexamethasone	26	53.7±12.9	18	NR	56.5 ± 13.4	1.43±0.6	NR	NR
**Group 2**: IT Gentamicin	31	54.2±12.9	21	NR	58.7 ± 13.3	1.45±0.59	NR	NR
**Albu (2016)** [40]							II: 6, III: 18,	
**Group 1**: ITD (Dexamethasone) +placebo	32	NR	21	NR	51.4± 13.6	NR	IV: 9;	7.5±NR
**Group 2**: ITD (Dexamethasone)+ high dosage betahistine (HDBH)	30	NR	18	NR	54.6± 15.2	NR	II: 7, III: 19, IV: 7	6.7±NR
**Bremer (2014)** [43]					Rt ear:59± 8.6			
**Group 1**: IT Gentamicin	5	64.5±8	2	NR	Lt ear: 46.8±21.3;	3.1 (1.1–19.6)	NR	NR
					Rt ear:29.7± 7.4, Lt ear: 61.3±30.7;			
**Group 2**: IT Gentamicin+placebo	5	72.6±5	0	NR	Rt ear:35± 24.5, Lt ear: 43.8±21.6	3.3 (0.7–7.5)	NR	NR
**Group 3**: placebo	4	57.3±16.7	4	NR		2.5 (0.1–18.2)	NR	NR
**Postema (2008)** [46]					Diseased ear: 56			Vertigo score***: 2.1±0.8;
**Group 1**: IT gentamicin	16	55(NR-NR)	NR	NR	Contralateral ear:16	NR	NR	2.0±0.8
**Group 2**: Placebo					Diseased ear: 53			
	12	53(NR-NR)	NR	NR	Contralateral ear:18	NR	NR	
**Ganança (2009)** [45]								
**Group 1**: Low-dose betahistine (16 mg tid)	60	45.9±13.3	37	NR	NR	1.4±NR; 12(2–48) □	NR	76.6% †
**Group 2**: High-dose betahistine (24 mg bid)	60	45.4±13.8	36	NR	NR	1.34±NR 14(4–48) □	NR	73.4% †
**Saliba (2015)** [57]								
**Group 1**: Endolymphatic duct blockage (EDB)	35	47.2±NR	21	NR	50.9±30.2	NR	NR	8.37±5.8 ǂ
**Group 2**: Endolymphatic sac decompression (ESD)	22	53.5±NR	13	NR	55.8±23.9	NR	NR	9.64±7.9 ǂ
**Garduño-Anaya (2005)** [49]								
**Group 1**: IT Dexamethasone	11	Overall mean: 50; median 48 (range 28–77)	16	Overall: 10:12	55.7±NR	Overall mean: 6.9; median 5 (range 3–30)	NR	0.87±0.61
**Group 2**: Placebo	7		(all)		56.6±NR		NR	1.03±0.80
**Stokroos & Kingma (2004)** [50]			9					
**Group 1**: IT Gentamicin	12	59 (34–74)	(all)	Overall: 8:14	60±18.7 ∫	NR	NR	74±114 ‡
**Group 2**: Placebo	10	58 (45–70)			53±16.5 ∫	NR	NR	25±31 ‡
**ElBeltagy (2012)** [51]			11					Overall: 43.8±63.8 in last 6 months
**Group 1**: IT Gentamicin	15	Overall: 42 (29–57)	(all)	Overall: 19:11	57.7±10.6	Overall: 4±4.9; range 1–17	NR	
**Group 2**: IT Dexamethasone	15				47.5±8.33		NR	
**Kitahara (2008)** [47]							I:4, II: 18, III: 60, IV: 18	
**Group 1**: Endolymphatic sac drainage + steroid instillation into the sac	100	50.3± 14.5	56	NR	NR	8.7±7.5	I:3, II: 5, III: 32, IV: 7;	3.8±2.3
**Group 2**: Endolymphatic sac drainage without steroid instillation into the sac							I: 3, II: 8, III: 27, IV: 12	
**Group 3**: Medical managements included diuretics, betahistine, diphenidol, dimenhydrinate, and diazepam	47	55.6± 10.1	26	NR	NR	9.5±8.9		3.9±2.6
	50	53.7± 12.4	26	NR	NR	8.8±6.2		3.6±2.3
**Adrion (2016)** [41]								
**Group 1**: Low-dose betahistine	70	56.1± 11.1	34	28:21:00	NR	NR	NR	NR
**Group 2**: High-dose betahistine	72	56.1± 12.6	39	24:25:00	NR	NR	NR	NR
**Group 3**: Placebo	72	54.5± 12.8	39	28:25:00	NR	NR	NR	NR
**Morales-Luckie (2005)** [48]								
**Group 1**: Oral Prednisolone: [Prednisolone + diphenidol + acetazolamide + low-sodium diet (< 1,500 mg/d)]	8	40.8 (33–47)	NR	NR	NR	3.6	NR	C:8
**Group 2**: maintenance therapy: diphenidol + acetazolamide + low-sodium diet (< 1,500 mg/d)	8	38.8 (32–49)	NR	NR	NR	3.3	NR	C:8

* SE (standard error)

** six months before the start of treatment

*** vertigo scores based on a form to score vertigo on a four-point scale: severe (3), moderate (2), mild (1) and none (0).

^∫^ extended Fletcher index (dB) at 500, 1000, 2000 and 4000 Hz (AAO-HNS stage 3–4) before treatment

^∫∫^ data was not reported for MD patients separately.

^†^ percent of patients have ≥ 2 spells of vertigo lasting≥ 1 hour: 76.6% at baseline.

^‡^ number of attacks per year

^ǂ^ number of spells patient reported for the last 6 months before the surgery

^□^ data shown in months

**Abbreviations**: A = Complete control of definitive spells (numeric value 0–40); B = Limited control of definitive spells (numeric value 41–80); C = Insignificant control of definitive spells (numeric value 81–120); /d = per day; F = female; IT = intratympanic; L = left; Lt = left; M (SD) = Mean and standard deviation; M (range) = Median or Mean (range); mg = milligram; N = sample size analyzed; NR = not reported; PTA = pure tone average; R = right; Rt = right; & = and.

### Intervention and comparators

Of the 16 studies included in data syntheses, two compared surgical procedures (endolymphatic sac decompression (ESD) with steroid injection versus ESD without steroid injection [35]; and endolymphatic duct blockage (EDB) versus ESD [57]), one assessed oral medications (including diuretics, betahistine, diphenidol, dimenhydrinate, and diazepam), with and without tympanic ventilation [39], and the remaining trials compared various medical treatments to each other. IT dexamethasone was compared to IT methylprednisolone [36], high-dose betahistine [42], IT gentamicin [44, 51], IT dexamethasone plus high-dose betahistine [40], placebo [49], and systemic pharmaceutical therapy (salt and caffeine restricted diet, nicotine and alcohol restrictions, cinnarizine for acute episodes and low-dose betahistine hydrochloride for maintenance therapy) [37]. IT gentamicin was compared to IT methylprednisolone [38], IT dexamethasone [44, 51] and placebo [46, 50]. High-dose betahistine was compared to low-dose betahistine [41], IT dexamethasone [42] and placebo [41]. One study compared prednisolone plus maintenance therapy (diphenidol + acetazolamide + low-sodium diet < 1,500 mg/d) to the same maintenance therapy alone [48]. High-dose betahistine refers to 144 mg/day while low-dose betahistine refers to 48 mg/day. Details regarding dosage and treatment protocol are presented in SA2 Table in S3 Text.

### Outcomes reported

The following outcomes of interest were reported: hearing changes (improvement or deterioration) based on pure tone audiometry in eleven studies [35, 38, 40–42, 44, 46, 49–51, 57], patients with improved hearing in ten studies [35–40, 42, 44, 48, 49], Speech Discrimination Score (SDS)/Speech Recognition Threshold (SRT) in eight studies [37, 38, 40, 42, 44, 49, 51, 57], vertigo control in 13 studies [36–40, 42, 44, 46, 48–51, 57], vertigo frequency in ten studies [35, 38, 41, 42, 45, 48–51, 57], aural fullness in seven studies [37, 38, 46, 48, 49, 51, 57], and harms in six studies [38, 41, 42, 44, 48, 57]. Other endpoints including various measures of handicap, impairment of quality of life and functional impairment and disability were also reported. For instance, the Tinnitus Handicap Inventory (THI) and tinnitus related endpoints were reported in 11 studies [35, 37, 38, 40–42, 46, 48, 49, 51, 57], the Dizziness Handicap Inventory (DHI) and dizziness were reported in six studies [35, 38, 41, 42, 49, 51], the Meniere’s Disease Outcomes Questionnaire (MDOQ) was reported in one study [35], Functional Level Scale (FLS) was reported in five studies [38, 40, 42, 44, 49], the total Vestibular Disorders Activities of Daily Living (VDADL) was reported in one study [41], Self-rating Depression Scale and Stress Response Scale-18 (SRS-18) was reported in one study [39], and self-assessed functional disability was described in one study [48].

Length of follow-up varied between studies from a few weeks to years; the review focuses on the longest time point reported from six months to two years with complete reporting of data and adherence of protocol.

**Fig 2:**
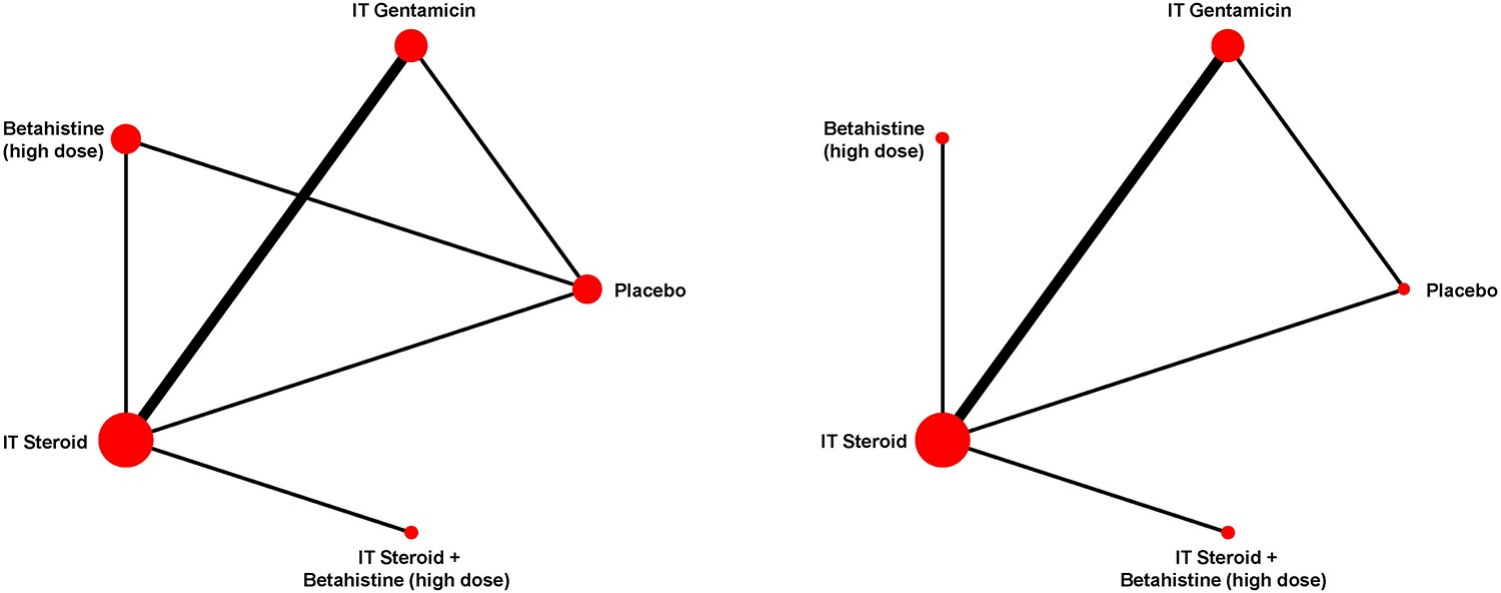
Network diagram of hearing change (left) and complete vertigo control (right). Each line links interventions and comparators directly compared. The size of treatment nodes was weighted by the number of patients, while the width of the edges each representing a pairwise comparison was weighted by the number of studies. Note: IT steroid plus high-dose betahistine has not been compared in head-to-head trials against other interventions except for IT steroid alone.

Of note, NMA was conducted only for hearing change and complete vertigo control because only these outcomes had closed loops of head-to-head comparisons in their network diagrams (see Fig 2).

### Findings from risk of bias assessment

Of the 16 included RCTs, nine had a high risk of bias [35, 36, 39, 42, 44, 45, 48, 49, 57], and six were judged to be at unclear risk of bias [37, 40, 41, 46, 50, 51] for all reported outcomes of interest, but one study had a low risk of bias judgment for two outcomes and an unclear risk of bias assessment for four outcomes [38]. The outcome-specific rating across all reported outcomes in trials changed the domain- specific rating in only three studies [35, 38, 46], but the overall rating was affected in only one study [38]. As such, the overall risk of bias ratings in 15 trials apply to all reported outcomes within these trials. The findings for these 16 studies are presented under summarized Risk of Bias Assessment in S3 Text. Two trials that were not included in the synthesis were assessed to be at high risk of bias due to concerns in selection bias and attrition bias [43, 47]. Further details on these two trials are presented in SA4a and SA4b Table in S3 Text.

**Fig 3:**
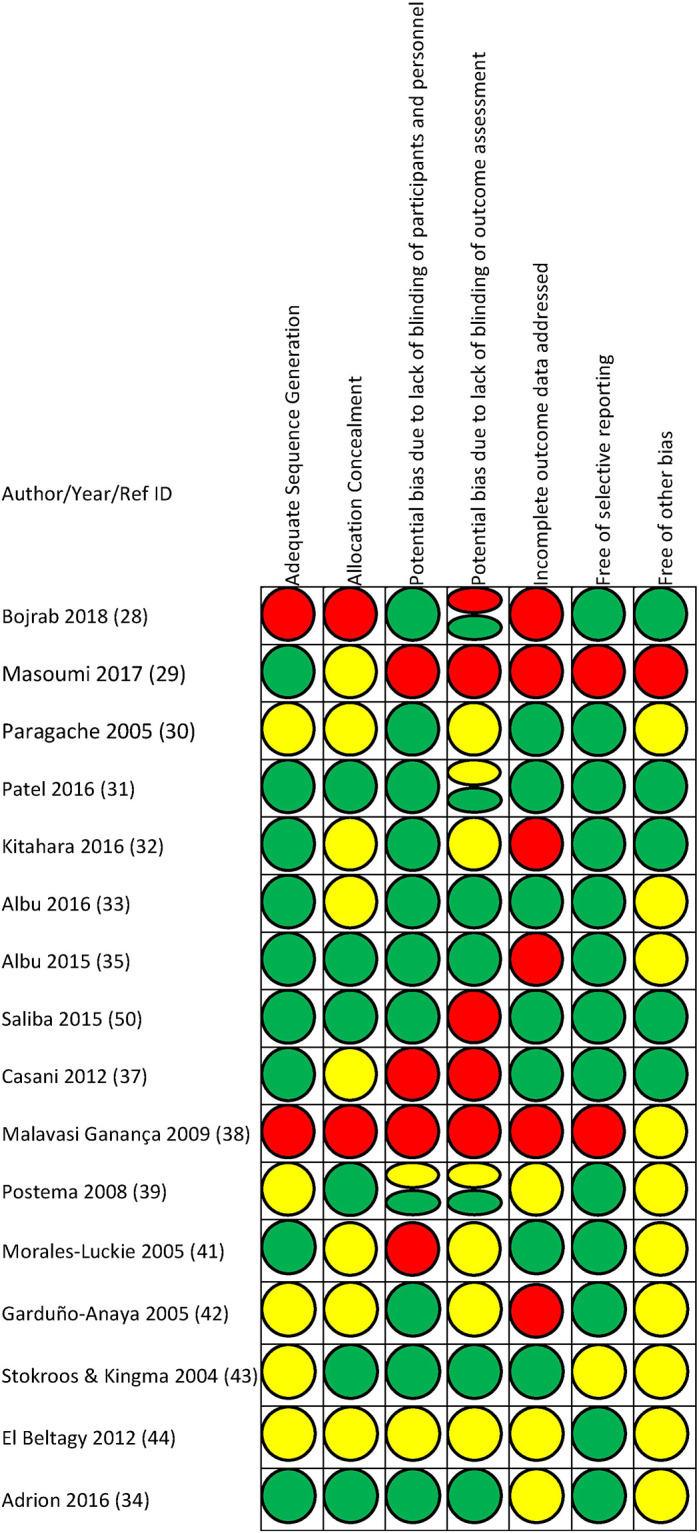
Findings from risk of bias evaluation. Findings from risk of bias evaluations using the Cochrane Scale are shown. Green = low risk of bias, red = high risk of bias, yellow = unclear risk of bias.

The outcome specific risk of bias status of the individual RCTs is presented in Fig 3, and details of each assessment are provided in SA3 Table in S3 Text.

### Findings: Hearing changes

Hearing changes were measured through pure tone average (PTA) change from baseline in eleven studies [35, 38, 40–42, 44, 46, 49–51, 57], based on the number of patients with improved hearing in ten studies [35–40, 42, 44, 48, 49] and changes in Speech Discrimination Score (SDS)/Speech Recognition Threshold (SRT) in eight studies [37, 38, 40, 42, 44, 49, 51, 57]. The thresholds were reported as: 1) PTA improvement ≥ 10 dB or > 15% SDS improvement [36, 40, 42, 48] or 2) PTA improvement ≥ 10 dB alone [37–39, 44, 49], while and one trial did not specify any definition [35]. We conducted both pairwise meta-analyses and NMA for hearing change. Findings regarding “improved hearing ≥ 10 dB” and changes in SDS/SRT are presented in S4 Text.

#### Hearing change (PTA): Findings from pairwise meta-analyses

Of the 11 studies that reported hearing change [35, 38, 40–42, 44, 46, 49–51, 57], two [41, 46] reported mean hearing change and the corresponding standard deviation (SD) or confidence interval per arm, while another trial [49] reported individual level data which allowed us to calculate the mean hearing change and the corresponding SD per arm, as well as the correlation between pre- and post-intervention hearing levels (0.859 based on all 22 patients). Before analyses, we calculated the mean hearing changes and the corresponding SDs for the other six studies [38, 40, 42, 44, 50, 51], assuming a fixed correlation of 0.859 between pre- and post-intervention hearing levels. Two trials comparing surgical treatments reported insufficient information regarding the number of patients beyond baseline [35, 57], and therefore were not included in meta-analyses.

**Fig 4:**
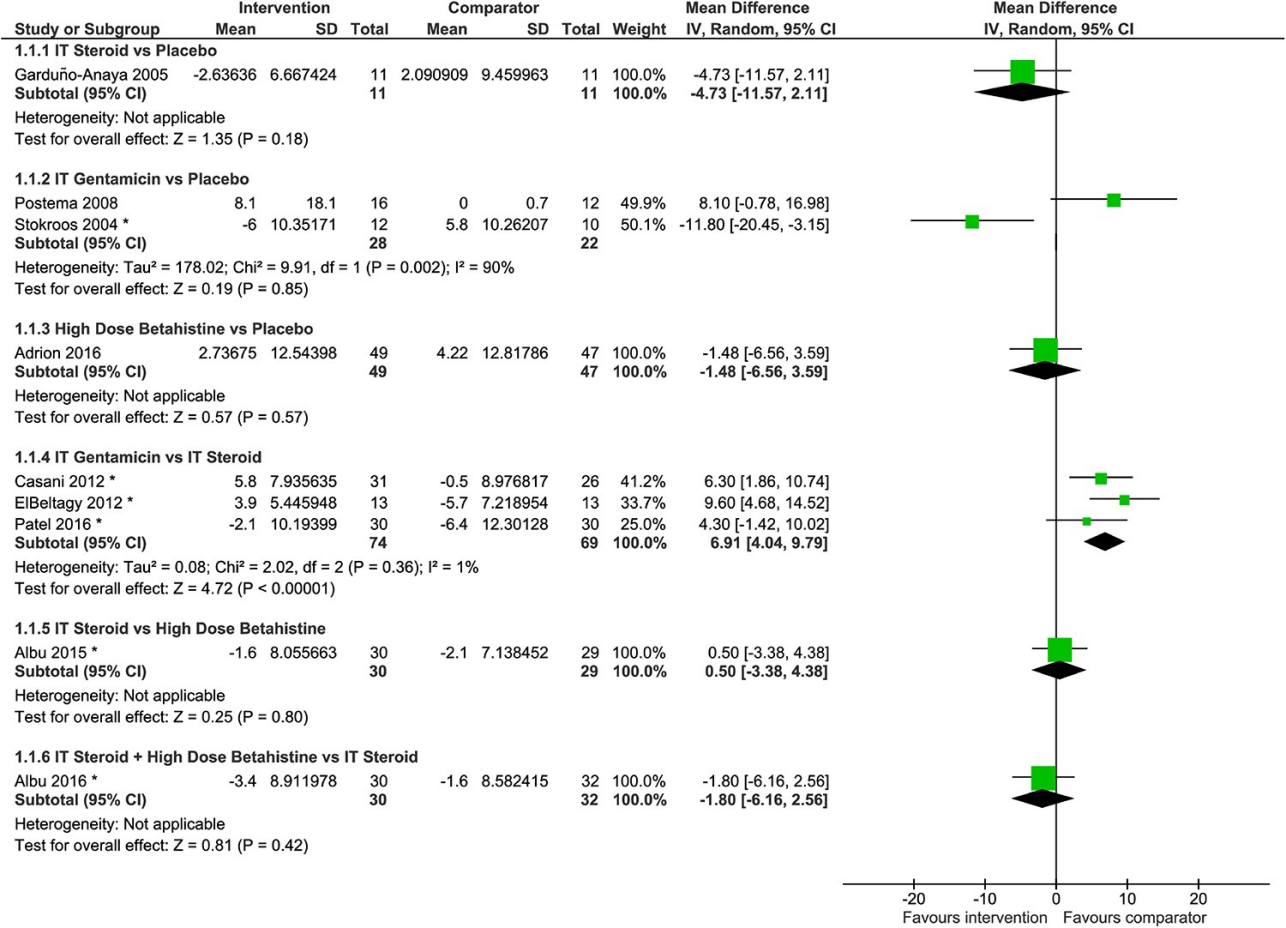
Difference in hearing change, where positive pure tone average (PTA) change per group indicates hearing deterioration. *Six studies [38, 40, 42, 44, 50, 51] reported the means and SDs before and after intervention but not the SDs of PTA changes. We calculated the mean PTA changes and the corresponding SDs, assuming the correlation of 0.859 before and after intervention. This correlation had been calculated from individual level data of 22 patients [49].

Meta-analysis of hearing change was performed for IT gentamicin versus IT steroid [38, 44, 51], but not for IT gentamicin versus placebo [46, 50] due to the presence of contradictory directions of effect size between the two studies with data available (Fig 4). Negative hearing change indicates hearing improvement. Four pairwise comparisons in hearing change were informed by one study each [40–42, 49]. Findings from pairwise comparisons of interventions are as follows:

*Findings*, *IT steroid versus placebo*. Findings from a single trial [49] suggested that IT steroid may be more beneficial than placebo; however, the difference between interventions was not statistically significant (mean difference -4.73 dB, 95% CI -11.57 dB to 2.11 dB; n = 22).

*Findings*, *IT gentamicin versus placebo*. Two trials demonstrated contradictory results with regard to PTA change; one study showed 8.10 dB more hearing loss on average associated with IT gentamicin compared with placebo [46] (95% CI -0.78 to 16.98 dB), while the other trial favored IT gentamicin [50] (mean difference: -11.80 dB, 95% CI -20.42 to -3.15 dB). Meta-analysis was not performed for these two studies considering the contradictory direction of treatment effects with confidence intervals not overlapping. While the study populations were considered similar, the difference in treatment effects may be due to differences in dosage and frequency of administration of interventions. There was a longer time interval between injections and fewer total injections in Stokroos and Kingma [50] in which 4 mL of gentamicin 30 mg/mL was administered every six weeks until either control of symptoms was achieved or one of the exclusion criteria was met (mean number of injections ± SD was 1.5±0.51). In Postema et al [46], 0.4 mL of gentamicin sulfate 30 mg/mL was administered on a weekly basis for four weeks.

*Findings*, *high-dose Betahistine versus placebo*. No significant difference was observed between high-dose betahistine and placebo in terms of PTA change (mean difference: -1.48 dB, 95% CI -6.56 to 3.59 dB; n = 96) in a single trial [41]. Reported adjusted mean change at four frequencies 0.25, 0.5, 1, and 2 kHz showed no important differences between the groups.

*Findings*, *IT gentamicin versus IT steroid*. Based upon data from three trials [38, 44, 51], IT gentamicin was associated with some degree of hearing deterioration compared to pre-treatment, on average, while IT steroid might be beneficial to reduce hearing loss. According to the meta-analysis of PTA change, hearing deterioration was 6.91 dB worse in the IT gentamicin group compared to the IT steroid group (95% CI 4.06 to 9.79; n = 143, I^2^ = 1%). IT steroid has an advantage over IT gentamicin in terms of hearing preservation.

*Findings*, *IT steroid versus high-dose Betahistine*. There was no significant difference between IT steroid and high-dose betahistine in terms of PTA change (mean difference: 0.50 dB, 95% CI -3.38 to 4.38 dB; n = 59) based on findings from a single trial [42].

*Findings*, *IT steroid + high-dose Betahistine versus IT steroid*. There was no significant difference between IT steroid with and without high-dose betahistine (mean difference: -1.80 dB, 95% CI -6.16 to 2.56 dB; n = 62) based on data from a single trial [40].

*Findings*, *ESD with or without steroid injection*. No statistically significant difference in mean PTA was observed between ESD with or without steroid injection, (PTA at 0.5, 1, 2, and 4 kHz) (45.5 dB preoperatively and 50.8 dB at 6 months, 52.8 dB at 12 months and 44.4 dB at 24 months postoperatively in the steroid group; and 39.9 dB preoperatively and 38.1 dB at 6 months, 37.3 dB at 12 months and 37.6 dB at 24 months postoperatively in the non-steroid group). Investigators combined data from both groups and reported that hearing remained stable before and after surgery [35]. The number of patients per group beyond baseline was unclear, and thus the data could not be analyzed quantitatively.

*Findings*, *EDB versus ESD*. In a single study, there was no statistically significant difference in PTA (0.25, 0.5, 1, 2, and 4 kHz) between the groups at all follow-up times (pre- and post- surgery at one week, one month, six months, 12 months and 24 months). There was a significant increase at one week in both groups, but 24 months after surgery the PTA level returned to baseline levels with no difference compared to preoperative levels (p = 0.932 for the EDB group and p = 0.864 for the ESD group). The number of patients per group beyond baseline was unclear, and thus the data could not be analyzed quantitatively [57].

#### Hearing change (PTA): Findings from network meta-analysis

Head-to-head trials were available for six out of ten (60%) of the pairwise comparisons for hearing change (Fig 2 left panel). Evaluations of model fit suggested that the RE consistency model was an adequate fit to the data (see S4 Text), and furthermore that no severe violations of the consistency assumption were observed. Findings from this model are described next.

Nine studies [38, 40–42, 44, 46, 49–51] were included in the NMA of hearing change comparing five interventions. Based upon the traits of interventions and the existence of conflicting findings, Stokroos and Kingma [50] was excluded from NMA for reasons described earlier. A supplemental NMA presented in S4 Text included this study and considered IT gentamicin six weeks apart as a separate node from IT gentamicin.

**Fig 5:**
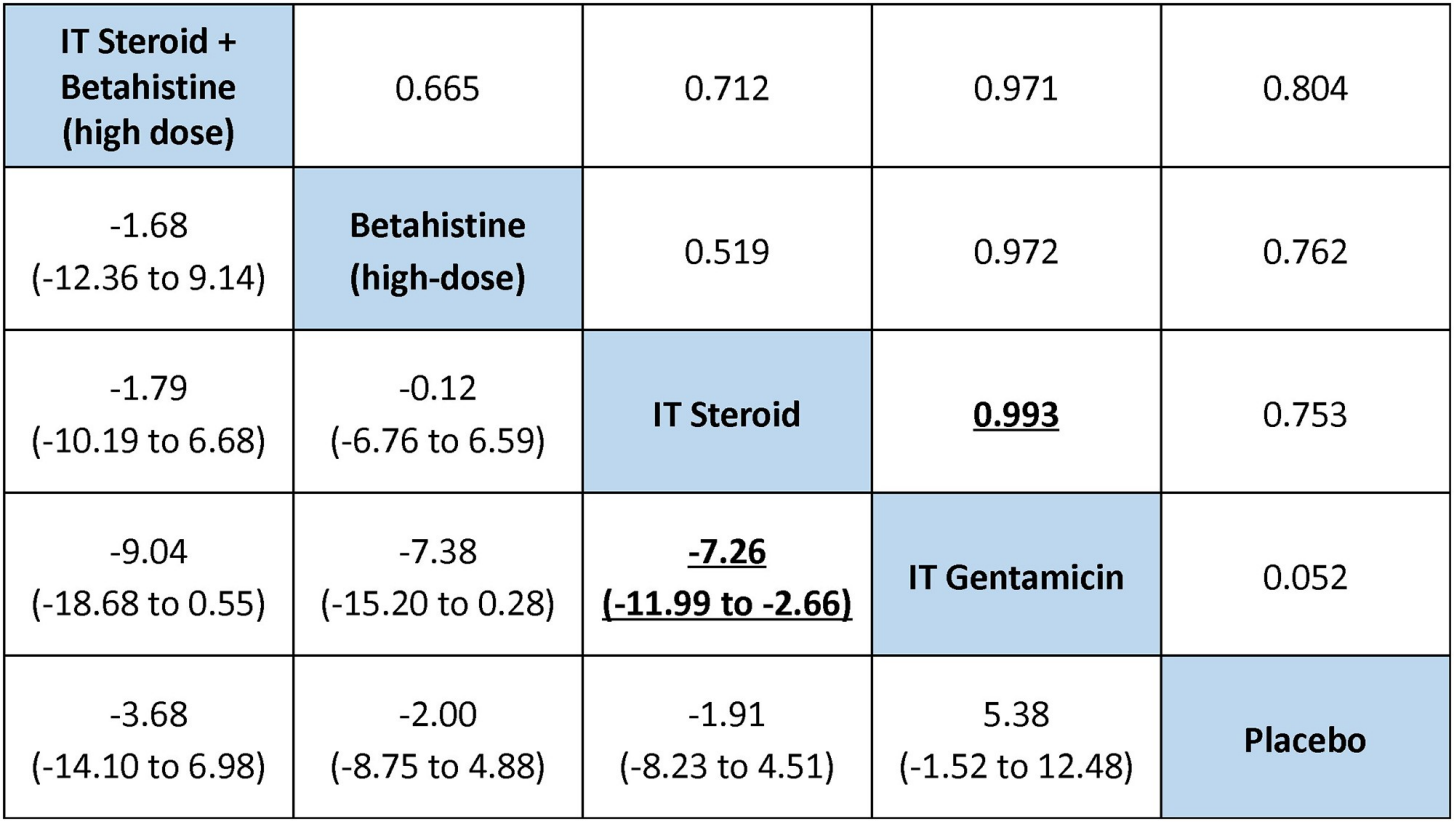
League table of pairwise difference estimates in hearing change (lower triangle), and the probabilities that a treatment is better than another (upper triangle). The league table presents pairwise differences in hearing change (PTA) with credible intervals (2.5% and 97.5% quantiles), and the pairwise probabilities that a treatment is better than another based on NMA. A complete summary of estimates for efficacy from the RE consistency model assuming vague priors is displayed. Estimates of difference in PTA change between regimens which ruled out the possibility of no difference are shown in bold, underlined font. For each comparison, the lower/right-most treatment is the reference treatment. For example, the largest difference in PTA improvement compared to placebo was associated with IT steroid + high-dose betahistine, estimated as -3.68 dB (95% CrI -14.10 to 6.98), with a negative sign indicating benefit over comparator in hearing preservation.

Fig 5 presents a league table of mean differences estimated from NMA, while SA6 Table in S4 Text presents a summary of the associated secondary measures (namely SUCRA, p(best) and treatment rankings). The largest difference in PTA change compared to placebo was associated with IT steroid plus high-dose betahistine: -3.68 dB (95% credible interval (CrI) -14.10 to 6.98 dB; SUCRA 0.788, mean rank 1.85), wherein the negative values denote benefit over placebo in hearing preservation. In order of descending SUCRA value, the next interventions were high-dose betahistine (-2.00 dB versus placebo, 95% CrI -8.75 to 4.88 dB; SUCRA 0.647, mean rank 2.41) and IT steroid (-1.91 dB versus placebo, 95% CrI -8.23 to 4.51 dB; SUCRA 0.629, mean rank 2.49). IT steroid was associated with better hearing improvement compared with IT gentamicin, with a -7.26 dB difference in PTA change (95% CrI -11.99 to -2.66 dB).

SA2 Fig in S4 Text presents a summary of the probabilities of each treatment to be at specific rankings. Based on summary estimates and corresponding 95% CrIs, IT steroid plus high-dose betahistine, high-dose betahistine, and IT steroid alone may be beneficial to reduce hearing loss compared to placebo. However, all pairwise comparisons were associated with 95% credible intervals that failed to rule out the possibility of no difference. The comparison-adjusted funnel plot (SA3 Fig in S4 Text) detected no evidence of small-study effects.

### Findings: Vertigo

Vertigo was assessed through measurement of vertigo control in 13 studies [36–40, 42, 44, 46, 48–51, 57], changes in vertigo frequency in ten studies [35, 38, 41, 42, 45, 48–51, 57] and vertigo severity in two studies [38, 46]. The AAO-HNS classes for vertigo control include complete control (class A: numeric value 0) or substantial control (class B: 1–40), limited control (class C: 41–80), insignificant control (class D: 81–120), worse control (class E: >120), and secondary treatment initiated due to disability from vertigo (class F) [9]. Quantitative analyses were not performed for vertigo frequency and vertigo severity due to reasons outlined in S4 Text, where study-specific findings are narratively reported. Findings from vertigo control are presented next.

**Fig 6:**
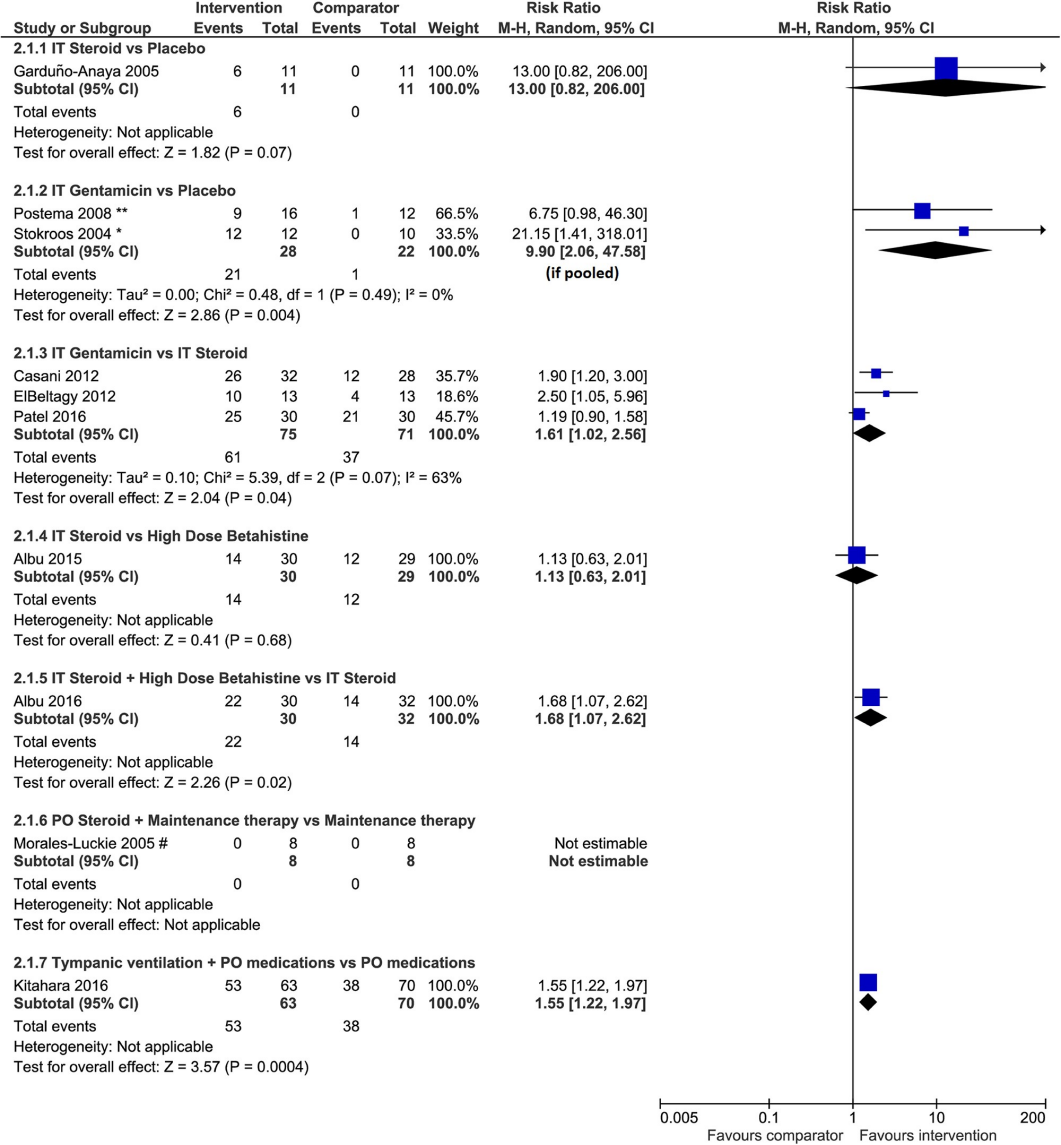
Risk ratio of complete vertigo control versus other categories as per the 1995 AAO-HNS definition. The AAO-HNS classes for vertigo control include complete (class A: numeric value 0) or substantial control (class B: 1–40), limited control (class C: 41–80), insignificant control (class D: 81–120), worse control (class E: >120), and secondary treatment initiated due to disability from vertigo (class F). * In Stokroos and Kingma [50], the number of patients with no complaints of vertigo attacks 6 weeks after the last treatment and during follow-up (6–28 months) had been used. ** In Postema et al [46], the number of patients with no complaints of vertigo (0 of a vertigo score which ranged 0 to 3) 12 months after treatment had been used. # In Morales-Luckie et al [48], maintenance therapy in both groups consisted of diphenidol (25 mg/d) plus acetazolamide (250 mg/48 h) and a low-sodium diet (< 1500 mg/d). Only patients with limited vertigo control (Class C) and severe disability (Scale 3) were included.

Vertigo control: **F**indings from pairwise meta-analyses. Of the thirteen studies that reported on vertigo control [36–40, 42, 44, 46, 48–51, 57], we performed pairwise meta-analyses for three studies that compared IT gentamicin with IT steroid [38, 44, 51], and two studies that compared IT gentamicin with placebo [46, 50]. The remaining pairwise comparisons had data from only one study each. The forest plots depict the risk ratio for complete vertigo control only (Fig 6) and for complete and substantial control of vertigo (SA7 Fig in S4 Text), mainly as per the 1995 definition of AAO-HNS [9]. Findings from pairwise comparisons of interventions are described next.

*Findings*, *IT steroid versus placebo*. Six of 11 patients who received IT steroid (dexamethasone) and zero of 11 patients who received placebo achieved complete vertigo control based on complete data at six months in one small trial [49]. IT steroid (dexamethasone) was associated with a benefit over placebo in terms of complete vertigo control (RR 13.00, 95% CI 0.82 to 206.00 if a continuity correction of 0.5 was applied; n = 22), as displayed in Fig 6. The difference was statistically significant (RR 4.50, 95% CI 1.25 to 16.25) for complete or substantial vertigo control [49] as demonstrated in SA7 Fig in S4 Text.

*Findings*, *IT gentamicin versus placebo*. IT gentamicin was associated with more benefit than placebo in terms of complete vertigo control (RR 9.90, 95% CI 2.06 to 47.58; I^2^ = 0%; n = 50) based on meta-analysis of two studies [46, 50] displayed in Fig 6, and complete control or significant reduction in the frequency of vertigo attacks (RR 7.05, 95% CI 1.59 to 31.32; N = 22) based on a single trial [50] as demonstrated in SA7 Fig in S4 Text. We considered patients with no vertigo complaints reported in the two studies to have experienced complete vertigo control.

*Findings*, *IT gentamicin versus IT steroid*. According to meta-analysis of complete vertigo control (class A), IT gentamicin may be more likely to help patients achieve vertigo control than IT steroid (RR 1.61, 95% CI 1.02 to 2.56; I^2^ = 63%; n = 146) based on three trials (Fig 6). The meta-analysis of complete or substantial control of vertigo (class A and B) found the difference was not statistically significant (RR 1.19, 95% CI 0.89 to 1.59; I^2^ = 83%), as displayed in SA7 Fig in S4 Text. The value of I^2^ showed considerable heterogeneity that may be associated with the types of steroids used (dexamethasone [44, 51] and methylprednisolone [38]), the dosages used (SA2 Table in S3 Text), and the baseline vertigo control status of the patients across the included studies. Patients within the trials were otherwise considered to be similar in terms of prerequisites regarding previous treatments.

*Findings*, *IT steroid versus high-dose Betahistine*. Based on one trial [42], there was no difference between IT steroid (dexamethasone) and high-dose betahistine in terms of complete vertigo control alone (RR 1.13, 95% CI 0.63 to 2.01; n = 59) displayed in Fig 6 and complete or substantial vertigo control (RR 1.14, 95% CI 0.76 to 1.69) demonstrated in SA7 Fig in S4 Text.

*Findings*, *IT steroid + high-dose Betahistine versus IT steroid*. IT steroid (dexamethasone) in combination with high-dose betahistine was more likely to help patients to achieve complete vertigo control than IT steroid without betahistine (RR 1.68, 95% CI 1.07 to 2.62; n = 62) as displayed in Fig 6. A similar benefit was observed for complete or substantial vertigo control (RR 1.37, 95% CI 1.04 to 1.81) based on a single trial [40] demonstrated in SA7 Fig in S4 Text.

*Findings*, *oral steroid + maintenance therapy versus maintenance therapy*. One trial [48] demonstrated that oral steroid (prednisone) combined with maintenance therapy may be beneficial compared to maintenance therapy alone among patients with limited vertigo control (class C) and severe disability, where maintenance therapy consisted of diphenidol (25 mg/d) plus acetazolamide (250 mg/48 h) and a low-sodium diet (< 1500 mg/d). However, the effect estimate was based on eight patients per group with wide 95% CI, and the lower 95% confidence limit sat upon the null value of 1 (RR 15.00, 95% CI 1.00 to 225.33, n = 16). No patient achieved complete vertigo control in the two groups.

*Findings*, *tympanic ventilation + oral medication (including diuretics*, *betahistine*, *diphenidol*, *dimenhydrinate*, *and diazepam) versus the same oral medication alone*. One trial [39] demonstrated a statistically significant difference favoring tympanic ventilation combined with oral medication against the same medication alone for complete vertigo control (RR 1.55, 95% CI 1.22 to 1.97; n = 133) demonstrated in SA7 Fig.

*Findings*, *IT steroid versus low-dose Betahistine + cinnarizine + diet restrictions*. There was no difference between IT steroid (dexamethasone) and conventional therapy comprising of salt and caffeine- restricted diet, nicotine and alcohol restrictions, cinnarizine (75 mg/day) for acute episodes and low-dose betahistine (48 mg/day) for maintenance therapy in terms of excellent and good vertigo control measured based on Sakata's criteria [58] (RR 1.13, 95% CI 0.55 to 2.32; n = 40) based on a single trial [37] demonstrated in SA7 Fig in S4 Text.

*Findings*, *IT steroid (methylprednisolone) versus IT steroid (dexamethasone)*. The difference between IT injection of the two steroids was not statistically significant with regard to complete or substantial vertigo control (RR 1.27, 95% CI 0.48 to 3.40; n = 69) according to data from one trial [36] demonstrated in SA7 Fig in S4 Text.

*Findings*, *EDB versus ESD*. One trial reported that 96.5% of patients in EDB group compared to 37.5% of the patients in ESD group achieved complete vertigo control 24 months after surgery (p = 0.002) [57].

#### Vertigo control: Findings from network meta-analysis

**Fig 7:**
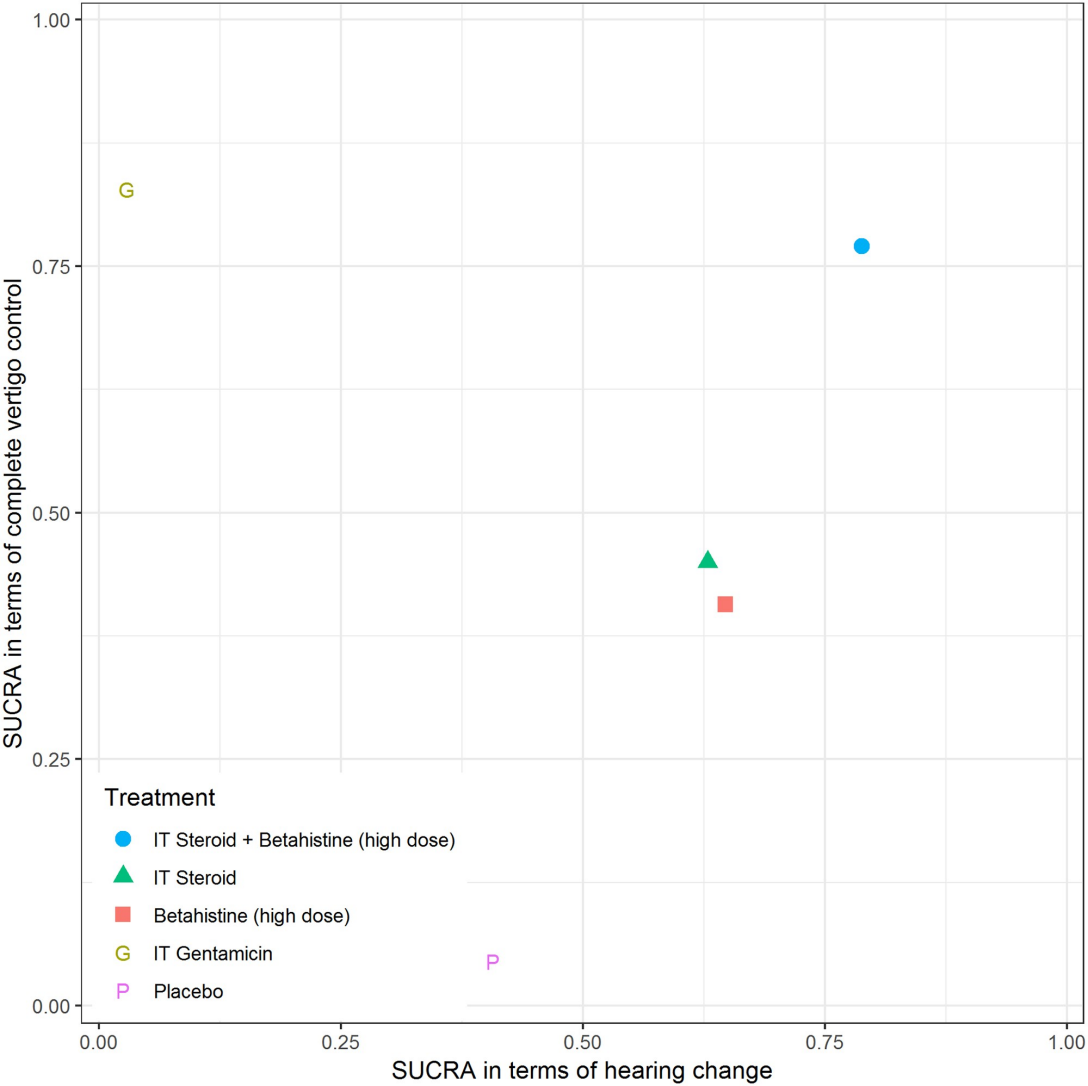
SUCRA values of treatments according to hearing change and complete vertigo control.

Head-to-head trials were available for 5 of 10 (50%) of the pairwise comparisons for complete vertigo control (Fig 2 right panel); there was no strong evidence of inconsistency (see S4 Text). Compared with the NMA of hearing change, the network diagram for the NMA of complete vertigo control contained no direct comparison between high-dose betahistine and placebo. Among all interventions, IT gentamicin was associated with the largest probability to achieve better performance in terms of complete vertigo control compared with placebo, followed by IT steroid plus high-dose betahistine. Comparisons of active interventions failed to rule out the possibility of no difference. Further details regarding NMA of complete vertigo control are presented in S4 Text. We generated a scatterplot of the SUCRA values of treatments according to NMA findings for complete vertigo control and hearing change (Fig 7), and found IT steroid plus high-dose betahistine was associated with the best balance of benefits across outcomes, followed by IT steroid and high-dose betahistine alone.

### Other measures of effectiveness

The presence of insufficient data and/or various metrics for the same outcome precluded pairwise meta-analyses for many endpoints. The pairwise analyses and narrative summaries of relevant findings are presented in S4 Text for these outcomes: SDS/SRT, aural fullness, tinnitus, Tinnitus Handicap Inventory (THI), Functional Level Scale (FLS), the total Vestibular Disorders Activities of Daily Living (VDADL), Dizziness Handicap Inventory (DHI) and dizziness related outcomes, self-assessed functional disability, MiniTF, Meniere’s Disease Ooutcomes Questionnaire (MDOQ), Self-Rating Depression Scale and Stress Response Scale-18 (SRS-18). Amongst these outcomes, FLS change echoed the findings of hearing change and vertigo control regarding the advantage of IT steroid plus high-dose betahistine over IT steroid and the advantage of IT steroid over placebo in pairwise analyses.

### Measures of patient harm

Six studies reported data on different harms of interest comparing various therapies that precluded meta-analyses and NMA [38, 41, 42, 44, 48, 57]. A narrative summary is presented next.

Two studies reported data regarding tympanic membrane perforation [42, 44]. One study compared IT dexamethasone plus placebo pill with IT placebo plus betahistine pills and reported tympanic membrane perforation in three patients that healed spontaneously [42]. The other study compared IT Gentamicin to IT dexamethasone and observed no cases of tympanic membrane perforation [44].

In terms of withdrawals due to adverse events, one study comparing oral prednisolone plus maintenance therapy (diphenidol plus acetazolamide plus low-sodium diet (<1,500 mg/d)) versus maintenance therapy alone reported one patient withdrawal in the prednisolone group due to bilateral malleolar edema [48]. Another study comparing high-dose betahistine to low-dose betahistine and to placebo reported totals of 11/74 (15%), 4/72 (6%), and 5/74 (7%) withdrawals, respectively [41].

Two studies reported data regarding the occurrence of serious adverse events [38, 41]. One study comparing IT methylprednisolone to IT gentamicin observed no serious side effects [38]. The other study compared high-dose betahistine, low-dose betahistine and placebo, and reported frequency of severe adverse events (SAEs) as at least one: 1) SAE, 2) severe treatment emergent adverse event (TEAE), and 3) treatment emergent serious adverse event (TESAE) [41]. TEAE was defined as an “adverse event that started or worsened in severity on or after the first study drug use and within 21 days of last study drug use”, and TESAE as “adverse event that was judged to be serious by the investigator and started at or after the first use of study drug and within the gap period (21 days) after the last study drug use, or an adverse event that already existed before the start of that treatment but worsened during the treatment and within the gap period including any subsequent washout or post-treatment period” [41]. At least one SAE was observed in 14/74 (19%) patients receiving high-dose betahistine, in 12/72 (17%) of patients receiving low-dose betahistine, and in 11/74 (15%) of patients receiving placebo. Similarly, at least one severe TEAE was recorded in 19/74 (26%) patients in the high-dose betahistine group, 20/72 (28%) patients in the low-dose betahistine group, and in 20/74 (27%) patients in the placebo group. At least one TESAE was reported in 10/74 (14%) of patients receiving high-dose betahistine, in 10/74 (14%) low-dose betahistine, and in 11/72 (15%) of patients on placebo [41].

One study comparing EDB to ESD reported that five patients initially underwent ESD but the operation failed, so these patients were reoperated for EDB [57]. Additionally, one may consider hearing loss with ITG as a harm of the drug. ITG may help vertigo control but may come at a cost to patient hearing, depending on dose, frequency and duration of therapy. Further details on this is provided in “hearing changes” section above and in S4 Text.

### Subgroup and sensitivity analyses

Due to a lack of data, we were unable to carry out analyses for the a priori subgroups of interest outlined in the study protocol.

In S4 Text, sensitivity analyses for each continuous outcome assuming an alternative correlation between baseline and final values are presented. Additionally, in SA4 Fig in S4 Text, an NMA for hearing change is presented that included data from Stokroos and Kingma and considered IT gentamicin with injections six weeks apart as a separate treatment node from IT gentamicin in other studies. IT gentamicin with a long interval between two injections (six weeks) and less cumulative dosage (1.5±0.51 injections) may be beneficial compared to placebo; however, future trials are needed to confirm this trend which was observed in a single study [50].

## Discussion

This systematic review assessed the effectiveness and harms of pharmacologic and surgical therapies for patients with MD. The evidence base for this review was limited in several aspects including single or few trials per treatment comparison, heterogeneous approaches to outcome measurement or use of various metrics that precluded pooling in some cases, and reporting limitations with data in the primary trials that prevented quantitative analyses in other cases. We conducted NMAs and pairwise MAs when data permitted, and measures of ranking hierarchy (i.e., SUCRA) suggested that the best balance in terms of hearing change and complete vertigo control was associated with IT steroid plus high-dose betahistine, followed by IT steroid and high-dose betahistine alone (Fig 7). While IT gentamicin was found to be the most effective for vertigo control, it may have an unfavorable effect on hearing preservation. It is important to note that IT steroid plus betahistine was assessed in only one trial judged to be at unclear risk of bias comparing it with IT steroid alone. Future head-to-head trials which compare it with placebo or other interventions are needed to confirm these results.

The two studies at unclear risk of bias that compared gentamicin with placebo demonstrated contradictory results in PTA change. Stokroos and Kingma [50] concluded that IT gentamicin with injections six weeks apart might be beneficial compared to placebo in terms of hearing preservation, while Postema et al. [46] observed IT gentamicin with injections one week apart to be associated with deterioration in hearing. Both trials were judged to be of unclear risk of bias. The discrepancy might be attributed to a larger time interval between injections or less gentamicin (1.5 ± 0.51 versus four injections) in the former study compared to the latter [59]. Such speculation should be verified through future trials. Meta-analysis revealed inferiority of IT gentamicin (one to three weeks apart) compared with IT steroid based on three trials, and results from NMA conveyed the same message. According to results from our NMA of hearing change, IT steroid plus high-dose betahistine was favored among the interventions compared and was associated with probabilities of 66.5%, 71.2%, 97.1%, and 80.4% to achieve better hearing preservation compared to high-dose betahistine, IT steroid, IT gentamicin (excluding injections six weeks apart), and placebo, respectively; the corresponding CrIs of difference in hearing change did not rule out the possibility of null difference.

In terms of complete vertigo control, both IT steroid plus high-dose betahistine and IT gentamicin were associated with an important advantage over IT steroid alone. However, when considering complete or substantial control, the advantage of IT gentamicin over IT steroid diminished to non-significant based on the same three studies [38, 44, 51], while the advantage of IT steroid plus high-dose betahistine over IT steroid alone was maintained [38]. Two of the trials were assessed to be at unclear risk of bias [31, 44] while one at high risk of bias [37]. Given the small number of trials per pairwise comparison, future trials are necessary to provide more evidence for or against these findings.

Other systematic reviews have recently been published in the field, and certain differences between the current review and NMAs conducted by Cao et al [60] and Hao et al [61] were noted. First, we summarized 16 trials which considered pharmaceutical interventions (via both systemic and IT routes of administration) and surgical interventions, and summarized data on various endpoints such as vertigo, hearing, aural fullness, tinnitus, FLS, DHI and other endpoints related to quality of life, handicap, and disability measures. Cao et al. included nine trials that compared five pharmaceutical medications (with dexamethasone and methylprednisolone considered as separate treatment nodes), focused on only IT administration and did not report any other endpoints such as hearing, tinnitus, aural fullness and adverse effects. Hao et al. [61] included ten trials of IT glucocorticoids and IT gentamicin compared with each other or placebo, and reported analyses for hearing change, frequency of vertigo attacks, vertigo control, FLS, SDS, and THI. Second, we conducted separate meta-analyses for complete vertigo control (class A per AAO-HNS 1995 definition, equivalent to no vertigo complaints) versus other categories, and complete and substantial control (class A and B) versus other categories, together with meta-analyses for hearing change and other outcomes. We performed NMA for hearing change and complete vertigo control, while vertigo frequency was considered too skewed to be analyzed as a continuous outcome for meta-analysis and NMA. Cao et al performed an NMA of vertigo control based on a mixture of definitions for vertigo control (mostly class A and B, and no vertigo complaints). Hao et al [61] performed an NMA for vertigo frequency and complete and substantial vertigo control (class A and B), an NMA for the other four outcomes which contained no head-to-head comparisons between IT gentamicin and placebo, and meta-analyses between glucocorticoids and IT gentamicin (61). Third, there were differences in the data usage for vertigo control compared with the current review that are detailed in S5 Text. Considering the differences above, our review has included more studies and interventions and did not have the same results for vertigo control compared with the two systematic reviews [60, 61] which incorporated NMA. As such, based upon differences in approach, we did not seek to compare and contrast our results relative to these other NMAs.

Wright et al 2015 [62] reported limited evidence that IT gentamicin may reduce vertigo symptoms (severity and frequency) compared to placebo based on two trials with very weak evidence. Pullens et al 2011 [59] summarized similar results regarding vertigo severity and frequency, tinnitus severity, aural fullness, but found a contradictory message regarding hearing changes between the same two studies [46, 50]. We identified both RCTs and narratively summarized similar results in these outcomes, and additionally found IT gentamicin to be more helpful than placebo in terms of complete vertigo control through meta-analysis.

Certain limitations of our review should be noted, several of which relate to issues in the set of included studies. Incomplete reporting of study design features pertaining to the trials’ risk of bias assessment was common, and lack of clarity with regard to reported data (e.g., use of imputation or last observation carried forward methods was not been mentioned when there were patients lost to follow-up, and the sample size of complete data was sometimes not clear) or lack of sufficient data (e.g., more than two thirds of the studies did not report the CIs/SEs of mean hearing change) were important limitations. The process of randomization is a vital feature of RCTs, and study authors should always aim to provide sufficient detail to inform reviewer judgments as to whether and how sequence generation and allocation concealment were carried out; however, several trials included in our review did not reach this standard. Additionally, in several studies there was a lack of clarity regarding who assessed patient outcomes and whether they were blinded to the trial intervention. Only two trials referenced an a priori protocol to assess if the trials were free of selective reporting. MD definition, intervention duration, outcome assessment time, sample size per group beyond baseline, whether intention-to-treat or per protocol analysis was used for a specific analysis, and study design features distinguishing RCT from observational studies were sometimes unclear. We contacted authors and requested the information; however, not all provided a response. We encourage the primary RCTs investigators to adhere to reporting guidelines when reporting their findings for clarity and completeness purposes and to reference their protocols to increase transparency. Only a few studies on surgical interventions which met our review eligibility criteria were included. Single primary studies compared ESD surgery with and without steroid treatment, and two surgical procedures (EDB versus ESD) precluded the possibility of meta-analysis or NMA and were therefore limited to narratively summaries. While our review is limited by the omission of a formal GRADE evaluation of the evidence as the optimal approach is currently unclear, we point readers to the many study limitations pointed to in our review when considering the clinical findings we have reported. Lastly, there may be skepticism amongst physicians as to the benefits of betahistine interventions for Meniere’s disease, and past guidance regarding its use has been variable; readers should consider the evidence presented in our review (and its limitations) in considering perspectives on its benefits for patients.

## Conclusion

To achieve both hearing preservation and vertigo control, the best pharmacologic treatment option among the interventions compared may be IT steroid plus high-dose betahistine, considering that IT gentamicin was found to be associated with benefits toward vertigo control but potentially detrimental effects on hearing preservation (often considered as chemical ablation) with high cumulative dosage and a short interval between injections. However, IT steroid plus high-dose betahistine has not been compared in head-to-head trials against other interventions except for IT steroid alone [40], and future trials which compare it with other interventions is crucial to establish comparative effectiveness with direct evidence. The superiority of IT steroid plus high-dose betahistine is thus yet to be confirmed. Clinicians may consider using high-dose betahistine first, followed by IT steroid plus high-dose betahistine, and if patients are willing to control vertigo at the risk of hearing deterioration, IT gentamicin. IT gentamicin or IT steroid plus high dose betahistine could be considered, if the patient is willing to stop the vertigo attacks regardless of the treatment effect on hearing; further research is needed to confirm whether IT gentamicin can control vertigo with adequate hearing preservation, if administered at a lower dose and with longer intervals between injections.

## Supporting information

S1 Text Deviations from study protocol. (DOCX)

S2 Text Search strategy. (DOCX)

S3 Text Study eligibility criteria, patient demographics and risk of bias. (DOCX)

S4 Text Additional analyses and measures of effectiveness reviewed. (DOCX)

S5 Text Differences between the current review and two existing SRs with NMA. (DOCX)

S6 Text Completed PRISMA NMA checklist. (DOCX)

S1 Data Study traits, interventions and patient demographics. (XLSX)

S2 Data Hearing change data. (XLSX)

S3 Data Vertigo control data. (XLSX)

S4 Data THI, Relief from aural fullness, FLS, VDADL, DHI. (XLSX)

## Abbreviations

AAO-HNS: American Academy of Otolaryngology-Head and Neck Surgery
COMET: Core Outcome Measures in Effectiveness Trials
DSR: Distiller Systematic Review
EDB: endolymphatic duct blockage
ESD: endolymphatic sac decompression
MD: Meniere's Disease
NMA: network meta-analysis
PICOTs: population, intervention, comparator, outcome, timing, study design
PRESS: peer review of electronic search strategies
PRISMA: Preferred Reporting Items for Systematic Review and Meta-Analysis
PROSPERO: Prospective Register of Systematic Reviews
PTA: pure tone average
QWB: Quality of Well-being Scale
SD: standard deviation
SDS: speech discrimination score
SNHL: sensorineural hearing loss
SR: systematic review
SRT: speech reception threshold
WRS: word recognition score
