# Supplementary material for: Pharmacologic and surgical therapies for patients with Meniere’s disease: A systematic review and network meta-analysis
Source: PLoS One. 2020 Sep 1;15(9):e0237523. doi: 10.1371/journal.pone.0237523 (PMC7462264; doi:10.1371/journal.pone.0237523)
Supplement: S1 Text — (DOCX) [file pone.0237523.s001.docx]

# S1 Text: Deviations from Study Protocol

Outcome measures only available from figures were extracted using a validated graphical digitizer (DigitizeIt, <https://www.digitizeit.de/>), via one reviewer and verified by another reviewer instead of dual independent extraction (because the software may provide slightly different estimates even if carried out by the same reviewer). Our protocol did not prespecify how to use individual patient data. As per a post-hoc decision, one reviewer obtained such data from the articles and calculated the aggregated summaries of interest (e.g. mean change and the corresponding standard deviation per arm for any continuous outcome), and another reviewer verified the extraction.

We decided not to consider fixed-effect NMA models; only random-effects NMA models were performed given the diversity of the study populations and interventions among the included studies.
