# Supplementary material for: Pharmacologic and surgical therapies for patients with Meniere’s disease: A systematic review and network meta-analysis
Source: PLoS One. 2020 Sep 1;15(9):e0237523. doi: 10.1371/journal.pone.0237523 (PMC7462264; doi:10.1371/journal.pone.0237523)
Supplement: S2 Text — (DOCX) [file pone.0237523.s002.docx]

# S2 Text: Search Strategy

*Reviews*

Database: Embase Classic+Embase <1947 to 2018 December 07>, Ovid MEDLINE(R) ALL <1946 to December 06, 2018>

Search Strategy:

--------------------------------------------------------------------------------

1 Endolymphatic Hydrops/ (9580)

2 Meniere Disease/ (16957)

3 meniere*.tw, kf. (13683)

4 ((acoustic* or auditory or aural or otogenic*) adj3 vertigo*).tw,kf. (473)

5 ((cochlea* or endolymphatic* or labyrinth*) adj3 hydrop?).tw,kf. (3486)

6 labyrinth* syndrome?.tw, kf. (81)

7 (labyrinth* adj3 vertigo*).tw,kf. (336)

8 or/1-7 [Meniere Disease] (20421)

9 exp Animals/ not Humans/ (16925146)

10 8 not 9 [ANIMAL-ONLY REMOVED] (17190)

11 (comment or editorial or news or newspaper article).pt. (1863046)

12 (letter not (letter and randomized controlled trial)).pt. (2055263)

13 10 not (11 or 12) [OPINION PIECES REMOVED] (16789)

14 limit 13 to systematic reviews [Limit not valid in Embase; records were retained] (8895)

15 meta analysis.pt. (94679)

16 exp meta-analysis as topic/ (56367)

17 (meta-analy* or metanaly* or metaanaly* or met analy* or integrative research or integrative review* or integrative overview* or research integration or research overview* or collaborative review*).tw,kf. (328507)

18 (systematic review* or systematic overview* or evidence-based review* or evidence-based overview* or (evidence adj3 (review* or overview*)) or meta-review* or meta-overview* or meta-synthes* or "review of reviews" or technology assessment* or HTA or HTAs).tw,kf. (390315)

19 exp Technology assessment, biomedical/ (23782)

20 (cochrane or health technology assessment or evidence report).jw. (38559)

21 (network adj (MA or MAs)).tw,kf. (18)

22 (NMA or NMAs).tw,kf. (4324)

23 indirect* compar*.tw, kf. (4563)

24 (indirect treatment* adj1 compar*).tw,kf. (633)

25 (mixed treatment* adj1 compar*).tw,kf. (1240)

26 (multiple treatment* adj1 compar*).tw,kf. (322)

27 (multi-treatment* adj1 compar*).tw,kf. (4)

28 simultaneous* compar*.tw, kf. (2170)

29 mixed comparison?.tw, kf. (54)

30 or/15-29 (683657)

31 13 and 30 (132)

32 14 or 31 [MENIERE REVIEWS] (8909)

33 32 use medall [MEDLINE RECORDS] (158)

34 inner ear disease/ (10103)

35 meniere disease/ (16957)

36 meniere*.tw, kw. (13951)

37 ((acoustic* or auditory or aural or otogenic*) adj3 vertigo*).tw,kw. (450)

38 ((cochlea* or endolymphatic* or labyrinth*) adj3 hydrop?).tw,kw. (3588)

39 labyrinth* syndrome?.tw, kw. (81)

40 (labyrinth* adj3 vertigo*).tw,kw. (406)

41 or/34-40 [Meniere Disease] (29203)

42 exp animal/ or exp animal experimentation/ or exp animal model/ or exp animal experiment/ or nonhuman/ or exp vertebrate/ (49260320)

43 exp human/ or exp human experimentation/ or exp human experiment/ (37874105)

44 42 not 43 (11387894)

45 41 not 44 [ANIMAL-ONLY REMOVED] (26629)

46 editorial.pt. (1066295)

47 letter.pt. not (letter.pt. and randomized controlled trial/) (2050274)

48 45 not (46 or 47) [OPINION PIECES REMOVED] (25943)

49 meta-analysis/ (248708)

50 "systematic review"/ (187806)

51 "meta-analysis (topic)"/ (39279)

52 (meta-analy* or metanaly* or metaanaly* or met analy* or integrative research or integrative review* or integrative overview* or research integration or research overview* or collaborative review*).tw,kw. (331314)

53 (systematic review* or systematic overview* or evidence-based review* or evidence-based overview* or (evidence adj3 (review* or overview*)) or meta-review* or meta-overview* or meta-synthes* or "review of reviews" or technology assessment* or HTA or HTAs).tw,kw. (393512)

54 biomedical technology assessment/ (22675)

55 (cochrane or health technology assessment or evidence report).jw. (38559)

56 (network adj (MA or MAs)).tw,kw. (18)

57 (NMA or NMAs).tw,kw. (4343)

58 indirect* compar*.tw, kw. (4626)

59 (indirect treatment* adj1 compar*).tw,kw. (637)

60 (mixed treatment* adj1 compar*).tw,kw. (1264)

61 (multiple treatment* adj1 compar*).tw,kw. (328)

62 (multi-treatment* adj1 compar*).tw,kw. (4)

63 simultaneous* compar*.tw, kw. (2170)

64 mixed comparison?.tw, kw. (55)

65 or/49-64 (744580)

66 48 and 65 [MENIERE - REVIEWS] (303)

67 66 use emczd [EMBASE RECORDS] (205)

68 33 or 67 [BOTH DATABASES] (363)

69 remove duplicates from 68 (284)

70 69 use medall (157)

71 69 use emczd (127)

***************************

*RCTs*

Database: Embase Classic+Embase <1947 to 2018 December 07>, Ovid MEDLINE(R) ALL <1946 to December 06, 2018>

Search Strategy:

--------------------------------------------------------------------------------

1 Endolymphatic Hydrops/ (9580)

2 Meniere Disease/ (16957)

3 meniere*.tw, kf. (13683)

4 ((acoustic* or auditory or aural or otogenic*) adj3 vertigo*).tw,kf. (473)

5 ((cochlea* or endolymphatic* or labyrinth*) adj3 hydrop?).tw,kf. (3486)

6 labyrinth* syndrome?.tw, kf. (81)

7 (labyrinth* adj3 vertigo*).tw,kf. (336)

8 or/1-7 [Meniere Disease] (20421)

9 exp Animals/ not Humans/ (16925146)

10 8 not 9 [ANIMAL-ONLY REMOVED] (17190)

11 (comment or editorial or news or newspaper article).pt. (1863046)

12 (letter not (letter and randomized controlled trial)).pt. (2055263)

13 10 not (11 or 12) [OPINION PIECES REMOVED] (16789)

14 (controlled clinical trial or randomized controlled trial or pragmatic clinical trial).pt. (560339)

15 clinical trials as topic.sh. (185396)

16 exp Randomized Controlled Trials as Topic/ (276400)

17 (randomi#ed or randomly or RCT? or placebo*).tw,kf. (2101152)

18 ((singl* or doubl* or trebl* or tripl*) adj (mask* or blind* or dumm*)).tw,kf. (385113)

19 trial.ti. (457361)

20 or/14-19 (2697694)

21 13 and 20 [MENIERE DISEASE - RCTS] (578)

22 21 use medall [MEDLINE RECORDS] (327)

23 inner ear disease/ (10103)

24 meniere disease/ (16957)

25 meniere*.tw, kw. (13951)

26 ((acoustic* or auditory or aural or otogenic*) adj3 vertigo*).tw,kw. (450)

27 ((cochlea* or endolymphatic* or labyrinth*) adj3 hydrop?).tw,kw. (3588)

28 labyrinth* syndrome?.tw, kw. (81)

29 (labyrinth* adj3 vertigo*).tw,kw. (406)

30 or/23-29 [Meniere Disease] (29203)

31 exp animal/ or exp animal experimentation/ or exp animal model/ or exp animal experiment/ or nonhuman/ or exp vertebrate/ (49260320)

32 exp human/ or exp human experimentation/ or exp human experiment/ (37874105)

33 31 not 32 (11387894)

34 30 not 33 [ANIMAL-ONLY REMOVED] (26629)

35 editorial.pt. (1066295)

36 letter.pt. not (letter.pt. and randomized controlled trial/) (2050274)

37 34 not (35 or 36) [OPINION PIECES REMOVED] (25943)

38 randomized controlled trial/ or controlled clinical trial/ (1272452)

39 exp "clinical trial (topic)"/ (283420)

40 (randomi#ed or randomly or RCT? or placebo*).tw,kw. (2103198)

41 ((singl* or doubl* or trebl* or tripl*) adj (mask* or blind* or dumm*)).tw,kw. (385265)

42 trial.ti. (457361)

43 or/38-42 (2872165)

44 37 and 43 [MENIERE - RCTs] (1085)

45 44 use emczd [EMBASE RECORDS] (732)

46 22 or 45 [BOTH DATABASES] (1059)

47 remove duplicates from 46 (815)

48 47 use medall (323)

49 47 use emczd (492)

***************************

*Non-RCTs*

Database: Embase Classic+Embase <1947 to 2018 December 07>, Ovid MEDLINE(R) ALL <1946 to December 06, 2018>

Search Strategy:

--------------------------------------------------------------------------------

1 Endolymphatic Hydrops/ (9580)

2 Meniere Disease/ (16957)

3 meniere*.tw, kf. (13683)

4 ((acoustic* or auditory or aural or otogenic*) adj3 vertigo*).tw,kf. (473)

5 ((cochlea* or endolymphatic* or labyrinth*) adj3 hydrop?).tw,kf. (3486)

6 labyrinth* syndrome?.tw, kf. (81)

7 (labyrinth* adj3 vertigo*).tw,kf. (336)

8 or/1-7 [Meniere Disease] (20421)

9 exp Animals/ not Humans/ (16925146)

10 8 not 9 [ANIMAL-ONLY REMOVED] (17190)

11 (comment or editorial or news or newspaper article).pt. (1863046)

12 (letter not (letter and randomized controlled trial)).pt. (2055263)

13 10 not (11 or 12) [OPINION PIECES REMOVED] (16789)

14 controlled clinical trial.pt. (92771)

15 Controlled Clinical Trial/ or Controlled Clinical Trials as Topic/ (566347)

16 (control* adj2 trial*).tw,kf. (545978)

17 Non-Randomized Controlled Trials as Topic/ (10275)

18 (nonrandom* or non-random* or quasi-random* or quasi-experiment*).tw,kf. (111490)

19 (nRCT or nRCTs or non-RCT?).tw,kf. (1721)

20 Controlled Before-After Studies/ (212507)

21 (control* adj3 ("before and after" or "before after")).tw,kf. (9021)

22 Interrupted Time Series Analysis/ (204649)

23 time series.tw, kf. (55428)

24 (pre- adj3 post-).tw,kf. (196923)

25 (pretest adj3 posttest).tw,kf. (10175)

26 Historically Controlled Study/ (222883)

27 (control* adj2 stud$3).tw,kf. (494131)

28 Control Groups/ (123916)

29 (control* adj2 group$1).tw,kf. (1106146)

30 trial.ti. (457361)

31 or/14-30 (3118790)

32 13 and 31 [MENIERE DISEASE - NON-RCTS] (978)

33 32 use medall [MEDLINE RECORDS] (510)

34 inner ear disease/ (10103)

35 meniere disease/ (16957)

36 meniere*.tw, kw. (13951)

37 ((acoustic* or auditory or aural or otogenic*) adj3 vertigo*).tw,kw. (450)

38 ((cochlea* or endolymphatic* or labyrinth*) adj3 hydrop?).tw,kw. (3588)

39 labyrinth* syndrome?.tw, kw. (81)

40 (labyrinth* adj3 vertigo*).tw,kw. (406)

41 or/34-40 [Meniere Disease] (29203)

42 exp animal/ or exp animal experimentation/ or exp animal model/ or exp animal experiment/ or nonhuman/ or exp vertebrate/ (49260320)

43 exp human/ or exp human experimentation/ or exp human experiment/ (37874105)

44 42 not 43 (11387894)

45 41 not 44 [ANIMAL-ONLY REMOVED] (26629)

46 editorial.pt. (1066295)

47 letter.pt. not (letter.pt. and randomized controlled trial/) (2050274)

48 45 not (46 or 47) [OPINION PIECES REMOVED] (25943)

49 controlled clinical trial/ (552801)

50 "controlled clinical trial (topic)"/ (9849)

51 (control* adj2 trial*).tw,kw. (550035)

52 (nonrandom* or non-random* or quasi-random* or quasi-experiment*).tw,kw. (111697)

53 (nRCT or nRCTs or non-RCT?).tw,kw. (1721)

54 (control* adj3 ("before and after" or "before after")).tw,kw. (9026)

55 time series analysis/ (22099)

56 time series.tw, kw. (56182)

57 pretest posttest control group design/ (357)

58 (pre- adj3 post-).tw,kw. (196949)

59 (pretest adj3 posttest).tw,kw. (10179)

60 controlled study/ (6357979)

61 (control* adj2 stud$3).tw,kw. (495650)

62 control group/ (123916)

63 (control* adj2 group$1).tw,kw. (1105911)

64 trial.ti. (457361)

65 or/49-64 (8240225)

66 48 and 65 [MENIERE - NON-RCTS] (2876)

67 66 use emczd [EMBASE RECORDS] (2294)

68 33 or 67 [BOTH DATABASES] (2804)

69 remove duplicates from 68 (2371)

70 69 use medall (507)

71 69 use emczd (1864)

***************************

Cochrane Library

Search Name: Hearing Loss - Meniere Disease

Date Run: 10/12/2018 16:55:48

Comment: Final - 2018 Dec 10

ID Search Hits

#1 [mh ^"Endolymphatic Hydrops"] 6

#2 [mh "Meniere Disease"] 138

#3 meniere*: ti,ab,kw 339

#4 ((acoustic* or auditory or aural or otogenic*) near/3 vertigo*): ti,ab,kw 15

#5 ((cochlea* or endolymphatic* or labyrinth*) near/3 hydrop*?): ti,ab,kw 26

#6 (labyrinth* next syndrome?): ti,ab,kw 0

#7 (labyrinth* near/3 vertigo*): ti,ab,kw 5

#8 {or #1-#7} 356

Cochrane Reviews n=7 [REVIEWS]

Protocols n=2 [REVIEWS]

Trials n=346 [RCTS]

Clinical Answers – 1 [*did not download*]
