# Supplementary material for: Pharmacologic and surgical therapies for patients with Meniere’s disease: A systematic review and network meta-analysis
Source: PLoS One. 2020 Sep 1;15(9):e0237523. doi: 10.1371/journal.pone.0237523 (PMC7462264; doi:10.1371/journal.pone.0237523)
Supplement: S3 Text — (DOCX) [file pone.0237523.s003.docx]

# S3 Text: Study eligibility criteria, patient demographics and risk of bias assessments

**Table A1:** Review eligibility criteria (research question and its PICOTS)

| Item | Description |
| --- | --- |
| **Research question** | What are the relative effects of pharmacologic therapies and surgical interventions in patients with MD on vertigo and other key patient outcomes in randomized controlled trials and quasi-randomized trials. |
| **Population** | Adult patients with MD per established criteria (i.e., American Academy of Otolaryngology-Head and Neck Surgery (AAO-HNS)) receiving pharmacologic or surgical interventions for their condition (e.g. endolymphatic sac decompression, intratympanic gentamicin injection, or others as detailed below) will be sought. |
| **Intervention/**  **Comparators** | - The following interventions will be of interest:   - *systemic pharmaceuticals*: diuretics (e.g., hydrochlorothiazide, furosemide), Motion sickness/anti-nausea medications (e.g., anticholinergics, antihistamines (betahistine), phenothiazines), benzodiazepines (e.g., diazepam);   - *Intra-tympanic pharmaceuticals*: intra-tympanic gentamicin, intra-tympanic steroids;   - *Surgical interventions*: sacculotomy, vestibular nerve section, labyrinthectomy, tympanostomy tube, endolymphatic duct blockage, endolymphatic sac decompression, endolymphatic shunt, transtympanic pressure treatment. |
| **Outcomes** | - Endpoints of interest will include   - frequency, severity, type, and control of vertigo measured via Electrochocleography score test or other methods;   - occurrence and intensity of tinnitus measured via various methods such as psychoacoustic tests (pitch match, loudness match, maskability, residual inhibition, etc.), rating scales (e.g., verbal rating scale, numerical rating scale, visual analog scale, poster style, mechanical device, etc.), questionnaires describing functional effects (e.g., tinnitus questionnaire, tinnitus handicap questionnaire, tinnitus severity scale, subjective tinnitus severity scale/tinnitus reaction questionnaire, tinnitus severity grading, tinnitus severity index, tinnitus handicap inventory, intake interview for tinnitus retraining therapy), and patients’ global perception of treatment-related changes);   - changes in hearing, based on Pure Tone Average (PTA) in decibels, and speech recognition, such as word recognition score (WRS) that may also be labelled as speech discrimination score (SDS), and speech reception threshold (SRT);   - quality of life measured by various scales such as Quality of Well-being Scale (QWB), SF-12, Physical SF-12 score; Mental SF-12 score, Center for Epidemiologic Studies–Depression Scale (CESD);   - perception of aural fullness;   - and harms, including hearing loss, withdrawals due to adverse effects, and serious side effects defined by authors. |
| **Timing** | Studies with a minimum follow up duration of six months after the first intervention |
| **Study Design** | Randomized controlled trials, and quasi-randomized trials will be included. |

**Table A2**: Study characteristics of the 16 studies included for quantitative or narrative analysis

| **Author (Publication Year)**  **Quality Assessment (QA); Time of outcome assessment used in meta-analyses/network meta-analyses** | **Inclusion Criteria**  **(underlined texts refer to MD definition; underlined and italic texts denote patients’ special characteristics); Interventions;**  **Treatment Protocol** |
| --- | --- |
| **Bojrab II (2018)** (28)  **High Risk of Bias**  We did not include this study in meta-analysis due to unclear number of patients beyond baseline. | **Inclusion criteria:** Definite Meniere’s disease as defined by 1995 AAO-HNS criteria: two or more definitive spontaneous episodes of vertigo lasting 20 minutes or longer, audiometrically documented hearing loss on at least one occasion, tinnitus, or aural fullness in the treated ear, other causes excluded. Additionally, *the patient had to have failed medical therapy (low-salt diet, diuretics, oral or trans-tympanic steroids) for 3 months*.  **Group 1:** Endolymphatic sac decompression surgery (ESD) + Steroid injection  **Group 2:** ESD  **Details of treatment protocol**: The technique of steroid injection was standardized among the surgeons. A 27-gauge needle on a 1 mL tuberculin syringe was used to inject ~ 0.2 to 0.4 ml of dexamethasone (10 mg/mL) into endolymphatic sac after decompression. Dilation of endolymphatic sac was visualized intraoperatively to confirm correct location of injection. If a surgeon was unable to definitively identify the endolymphatic sac, then the patient was not injected and was placed in the control group. |
| **Kitahara (2016)**(32)  **High Risk of Bias**  18-24 months after treatment | **Inclusion criteria:** Patients at least 20 years old and diagnosis of MD based on AAO-HNS 1995 criteria, Eligible *patients were those in whom 3–6 months of basically fixed forms of medical treatment had produced insufficient benefit* (*i.e. recurrent vertigo attacks and/or no improvement in sensorineural hearing loss) and for whom surgical treatment would otherwise be considered.*  **Group 1:** Tympanic ventilation tube + medication (including Diuretics, Betahistine, Diphenidol, Dimenhydrinate, and Diazepam)  **Group 2:** medication (including diuretics, Betahistine, Diphenidol, Dimenhydrinate, and Diazepam)  **Details of treatment protocol**: Ventilation tubes inserted through an incision in their tympanic membranes under local anesthesia to relieve inner ear hydrops. Medical treatments were fixed basically including diuretics, betahistine, diphenidol, dimenhydrinate, and diazepam. |
| **Masoumi (2017)**(29)  **High Risk of Bias**  6 months after treatment | **Inclusion criteria:** Definite MD diagnosis according AAO–HNS criteria, age greater than 18 years, no history of other otologic diseases, normal magnetic resonance imaging (MRI) scan and no history of neurological disorders. *Patients were refractory to treatment with salt restriction, diuretics, and betahistine for 3 months.*  **Group 1:** IT dexamethasone  **Group 2:** IT methylprednisolone  **Details of treatment protocol**: IT dexamethasone (4mg/dl), three injections were performed within a week. IT methylprednisolone (40mg/dl), three injections were performed within a week. |
| **Paragache (2005)** (30)  **Unclear Risk of Bias**  6 months from initiation of treatment | **Inclusion criteria:** Definitive cases of Meniere’s disease, as defined by the 1985 Committee on Hearing and Equilibrium Guidelines were included.  **Group 1:** IT Dexamethasone  **Group 2:** Conventional medical treatment (salt and caffeine restricted diet, and nicotine and alcohol restrictions, cinnarizine 25 mg three times a day for acute episodes, and betahistine 16 mg three times a day for maintenance therapy)  **Details of treatment protocol**: IT instillation of 5 drop of 0.2 mg/cc Dexamethasone eye/ear drops (Under local anesthesia, a Sheperd ventilation tube (grommet) was inserted into the posteroinferior quadrant of tympanic membrane. Dexamethasone eye/ear drops available commercially in the concentration of 1 mg/cc were diluted to 0.20 mg/cc using distilled water. 5 drops of medication was instilled into the middle ear. The patients were made to lie supine with medicated ear up for 30 minutes. Later self-instillation of drops was done by the patients for 3 months duration. Salt and caffeine restricted diet, nicotine and alcohol restrictions, Tab. Cinnarizine 25 mg TDS for acute episodes and Tab. Betahistine hydrochloride 16 mg TDS for maintenance therapy were given. |
| **Albu (2015)** (35)  **High Risk of Bias**  12 months after treatment | **Inclusion criteria:** Adult patients with unilateral definite MD fulfilling the criteria of the American Academy of Otolaryngology–Head and Neck Surgery (AAO-HNS 1995). All these *subjects failed a trial of 6 months of low-salt diet and diuretics*.  **Group 1:** IT Dexamethasone +placebo pills  **Group 2:** Betahistine + IT placebo (saline)  **Details of treatment protocol**: One group received a combination of IT dexamethasone (DX) and identical-appearing placebo pills while another group received a combination of high dosage betahistine (144 mg/day (48 mg tid) and IT saline. Dexamethasone and saline 3 IT injection with an interlude of 3 days of 1 mL of dexamethasone (4 mg/mL) were given following this procedure: in the supine position, the patient turned the head 45° toward the unaffected ear. Following anesthesia of the eardrum, using a 22-gauge spinal needle, 1 mL of dexamethasone (4 mg/mL) was injected through the tympanic membrane into the middle ear. Patients kept the supine position with the injected ear facing upward for 30 min refraining from swallowing or talking. The injection was repeated for three times with an interlude of 3 days (3 injections every 3 days) + identical appearing pills as betahistine. |
| **Patel (2016)** (31)  **Unclear Risk of Bias** (Vertigo, AEs, DHI, THI, FLS)  **Low Risk of Bias** (PTA, SDS)  24 months after treatment, or 18-24 months after treatment | **Inclusion criteria:** Patients aged 18–70 years with definite unilateral Meniere’s disease, defined according to the AAO-HNS (1995), who had experienced at least two episodes of rotational vertigo lasting at least 20 min in the previous 6 months and who had *shown no response to standard medical treatment* were eligible for inclusion.  **Group 1:** IT Methylprednisolone  **Group 2:** IT Gentamicin  **Details of treatment protocol**: Intratympanic injections of methylprednisolone (62·5 mg/mL) or Gentamicin (40 mg/mL) were performed in outpatient ENT clinics. Two injections were given, the second 2 weeks after the first. Since steroids do not disturb hearing, patients randomly assigned to methylprednisolone were given a second methyl prednisolone injection. For patients who had a 20 dB drop in hearing across any two consecutive frequencies, the pharmacy, without informing the trial team, switched gentamicin for saline. If vertigo attacks returned at any time during the trial (i.e., the patient was a non-responder) the unmasked clinician prescribed a further course of intratympanic injections, but the patient and everybody else involved remained masked to treatment allocation. The clinician had the choice to prescribe the same drug or swap, basing this decision on the patient’s response to previous injections. |
| **Casani (2012)** (37)  **High Risk of Bias** 24 months after treatment | **Inclusion criteria:** Patients affected with unilateral definite MD based on the criteria of the American Academy of Otolaryngology–Head and Neck Surgery (AAO-HNS 1995); *subjects had also undergone medical therapy (diuretics, betahistine, and low-salt diet) for at least 6 months*.  **Group 1:** IT Dexamethasone  **Group 2:** IT Gentamicin  **Details of treatment protocol**: Two mL of gentamicin sulfate (40 mg/mL) was buffered with 1 mL of sodium bicarbonate to obtain a 6.4-pH solution with 27.6-mg/mL concentration. Under the otomicroscope, patients were placed in supine position with the head turned 45 toward the unaffected ear. Local anesthesia was obtained by filling the external ear canal with lidocaine. Using a 22-gauge spinal needle and 1-mL syringe, the solution was injected through the mid posterior aspect of the eardrum to fill the middle ear. Patients were asked not to swallow or talk to prevent solution drainage from the eustachian tube, and they remained in the supine position with the affected ear facing up for 15 minutes—after which they were discharged. Patients were followed for 2 weeks to assess treatment outcome: it was effective if 1 or more bedside tests indicated a reduction of vestibular function. If the patient had not developed signs of vestibular hypofunction or a significant reduction of the caloric response on the treated ear, a second injection was planned after 20 days from the first injection.  IT Dexamethasone (4 mg/mL) perfusion repeated 3 times at intervals of 1 every 3 days using the same procedure. |
| **Albu (2016)** (33)  **Unclear Risk of Bias** 24 months after treatment | **Inclusion criteria:** Adult patients with unilateral definite MD according to the guidelines of the American Academy of Otolaryngology-Head and Neck Surgery (AAO-HNS) [1995]. According to prior recommendations, patients with episodes of spontaneous vertigo lasting between 20 min and 12 h were included. Further, as previously suggested, to be included, patients had to suffer a mean of four or more vertigo spells per month during the 3 months foregoing management. *All these patients failed a trial of 6 months of low-salt diet, dietary restrictions such as caffeine and nicotine avoidance*.  **Group 1:** ITD (Dexamethasone) + placebo  **Group 2:** ITD (Dexamethasone) + high dosage betahistine (HDBH)  **Details of treatment protocol**: Patients with definite MD were offered different therapeutic alternatives: IT injection of corticoid or IT injection of gentamicin and vestibular neurectomy. Patients elected ITD injection with or without HDBH as an opportunity that might provide transitory cessation of vertigo spells without the destruction of vestibular system. If complete or substantial vertigo control was not accomplished, another sequence of ITD was offered. In patients with persistent vertigo, despite repeated ITD injections, IT gentamicin injection or ablative surgery was offered. One group received three consecutive daily IT injections 1 ml of 4 mg/mL Dexamethasone + Placebo pills. Dexamethasone was injected under the microscope according to the guidelines [9–15]: in the supine position, the patient turned the head 45° toward the unaffected ear. Local anesthesia of the tympanic membrane was achieved and dexamethasone (4 mg/mL) was injected through a 22-gauge spinal needle and 1-mL syringe to fill the middle ear. Patients were instructed to keep the supine position with the treated ear facing upward for 30 min avoiding swallowing or talking. The other group received three consecutive daily IT injections 1 ml of 4 mg/mL Dexamethasone + Oral 144 mg/day (48 mg tid) of betahistine. |
| **Postema (2008)** (39)  **Unclear Risk of Bias** 12 months after treatment | **Inclusion criteria:** The AAO-HNS 1995 criteria was used for MD definition: two or more spontaneous episodes of vertigo each lasting 20 min or longer, sensorineural hearing loss documented audiometrically in the diseased ear and the presence of tinnitus or aural fullness (or both) in this ear. To be included in the study the patient’s most annoying complaint had to be vertigo, the vestibulum had to show a caloric response and *conservative medical treatment with betahistine was unsuccessful*.  **Group 1:**  IT gentamicin  **Group 2:** Placebo  **Details of treatment protocol**: A middle ear ventilation tube was introduced 4 weeks before the start of therapy. During 4 following weeks 0.4 ml of gentamicin sulfate (concentration 30 mg/mL) was injected once a week into the middle ear with a small needle through the ventilation tube of the intervention group. During 4 following weeks 0.4 ml of placebo was injected once a week into the middle ear with a small needle through the ventilation tube of the control group. |
| **Ganança (2009)** (38)  **High Risk of Bias**  24 weeks from initiation of treatment | **Inclusion criteria:** Patients had experienced two or more definitive spontaneous vertigo spells lasting 20 min or longer, had audiometrically documented hearing loss on at least one occasion, tinnitus or aural fullness (sounds) within the treated ear (AAO-HNS 1995). Adults completing 4, 12 and 24 weeks of continuous oral treatment with betahistine 16 mg tid or oral betahistine 24 mg bid were admitted.  **Group 1:** Low-dose Low-dose Betahistine (16 mg tid)  **Group 2:** High-dose Betahistine (24 mg bid)  **Details of treatment protocol**: Not reported. |
| **Saliba (2015)** (50)  **High Risk of Bias**  We did not include this study in meta-analysis due to unclear number of patients beyond baseline. (For example, 96.5% of the EDB group and 37.5% of the ESD group with vertigo control multiplied by 35 and 22 were not close to integer. | **Inclusion criteria:** Only patients in the definite category of the MD, a diagnostic scale based on AAO-HNS 1995, were included *who underwent medical therapy and CATS (caffeine, alcohol, theophylline [exists in tea and chocolate], and salt) restriction for at least 6 months without improvement* and who had more than 6 vertigo attacks for the last 6 months before the surgery. From the first day of the surgery all operated patients were asked *to stop all kind of medication* for MD and follow the CATS restriction.  **Group 1:** endolymphatic duct blockage (EDB)  **Group 2:** endolymphatic sac decompression (ESD)  **Details of treatment protocol**: Endolymphatic duct blockage (EDB); First, we performed a canal wall-up mastoidectomy: the tegmen mastoideum, sigmoid sinus, and sinodural angle are identified, and the posterior bony ear canal wall is thinned. We identify the posterior semicircular canal (PSCC) and the dura matter of the posterior fossa. Using the prominence of the horizontal semicircular canal, Donaldson line is identified to approximate the position of the endolymphatic sac. Bone over the sac and the dura are thinned with a diamond burrs. The sac is completely skeletonized and decompressed. The infralabyrinthine dura is exposed because the main body of the sac and its lumen often lie within this area. The sac is not incised neither dissected off the posterior fossa dura. For the ESD group, the sac is completely decompressed, and the surgery is accomplished. For the EDB we continue to dissect the bone of the vestibular aqueduct operculum and the posterior fossa dura from the retrolabyrinthine bone medial to the sac around the endolymphatic duct in order to identify the duct in its superior and inferior part in continuity from the endolymphatic sac and create a place to insert the tips of the instrument to clip the duct. At this level care must be taken not to traumatize the dura, which is often thin. Finally, we block the dissected endolymphatic duct with 2 small titanium clips. The titanium clips were applied by using the ligating clip applier, like the 1 used in vascular surgery. |
| **Garduño-Anaya (2005)**(42)  **High Risk of Bias**  Patients were followed up until 24 months after treatment, however, treatments beyond the protocol were applied and patient dropout happened starting from 6 months. Therefore, we only used data at baseline and 6 months. | **Inclusion criteria:** Patients having definite MD as outlined by the 1995 American Academy of Otolaryngology–Head and Neck Surgery Committee on Hearing and Equilibrium. They were older than 18 years of age, without any previous medical treatment with steroids or surgery for MD, were included in this study. Before the inclusion in this study *all the patients failed to respond to conventional medical therapy with caffeine and salt restriction (1500 mg/day), vasodilator, and diuretic given at least 6 months without any relief of vertigo attacks*.  **Group 1:** IT Dexamethasone  **Group 2:** Placebo  **Details of treatment protocol:** The patient lied flat on a stretcher in the office with the neck fully extended and the head turned 45 degrees to the opposite side. We used Emla cream (Lidocaine 2.5%/Prilocaine 2.5%, Astra Zeneca Pharmaceuticals, LP, Wilmington, DE) to regionally anesthetize the tympanic membrane in the quadrant anterosuperior of the involved ear. Dexamethasone solution 4 mg/mL or placebo consisting of saline solution were loaded into a 3-mL syringe on which is placed a 22-gauge needle. The tip of the needle was angled. Filling the middle ear with a simple needle injection was not difficult. After the injection was administered slowly, the patient was instructed to lie in the supine position with the treated ear up for one hour, to keep their head still, and to swallow as little as possible, with no talking. Subsequent injections were delivered in the same way each day for the next 4 days. After each day’s procedure the patient returned to his or her normal daily activities. |
| **Stokroos and Kingma (2004)** (43)  **Unclear Risk of Bias**  Follow-up time varied between 6 and 28 months | **Inclusion criteria:** Active MD according to AAO-HNS (1995) criteria, known underlying cause excluded using a diagnostic protocol, conservative/medical treatment for at least 6 months has proven unsuccessful, Incapacitating vertigo attacks occurring at least monthly and recorded for at least 6 months, Unilateral pathology, Informed consent obtained.  **Group 1:** IT Gentamicin  **Group 2:** Placebo  **Details of treatment protocol**: Gentamicin (4 ml; 30 mg/mL) in a buffered solution (pH 6.4) or placebo (buffer solution) was prepared in a 4-ml syringe and warmed in the physician’s breast pocket. The patient was in the supine position with the affected ear facing upwards local anesthesia of the tympanic membrane was achieved by application of a cottonoid soaked in 10% lidocaine spray against the tympanic membrane. A spinal puncture needle (15-cm long) was connected to the syringe and bent to an angle of 9/308. After aspirating any remaining lidocaine, a paracentesis was performed just anterior to the umbo. The needle tip was introduced slightly into the middle ear cavity. The hypo- and mesotympanum were filled with either gentamicin or placebo until the fluid meniscus was touching the paracentesis opening and fluid flowed back into the external meatus. The patient remained in this position for 45 min. Gentamicin or placebo was left in the middle ear. Applications were repeated every 6 weeks until either control of symptoms was achieved or one of the exclusion criteria was met. |
| **ElBeltagy (2012)** (44)  **Unclear Risk of Bias**  12 months after treatment | **Inclusion criteria:** Patients with unilateral definite Meniere’s disease based on AAO-HNS criteria (1995) with serviceable hearing (pure tone threshold <60dB and speech discrimination score better than 50%) and vestibular function in the unaffected ear, intractable attacks *not responding to medical treatment in the form of moduretic 5–50 (amiloride 5 mg and hydrochlorothiazide 50 mg) once daily + betaserc (betahistine 16 mg) three times daily for 6 months*, and absence of any associated neurological disorders.  **Group 1:** IT Gentamicin  **Group 2:** IT Dexamethasone  **Details of treatment protocol**: Patients in one group were treated with an intratympanic injection of 0.4 mL gentamycin 40 mg/mL that was buffered with sodium bicarbonate to pH 6.4 to reach a final concentration of 26.7 mg/mL. This was done on a weekly basis until the appearance of signs of unilateral vestibular hypoactivity, which was assessed by bedside tests (appearance of spontaneous nystagmus, head shake nystagmus, or head thrust sign), and the patient was subjected to three injections as a maximum.  Patients in another group were treated with an intratympanic injection of 0.4 mL dexamethasone 4 mg/mL every day for 5 consecutive days as a single cycle. |
| **Adrion (2016)** (34)  **Unclear Risk of Bias**  12 months after initiation of treatment (9 months of treatment and 3 months of follow-up) | **Inclusion criteria:** Patients aged 18-80 years were eligible for enrolment if they presented with two or more definitive spontaneous episodes of vertigo of at least 20 minutes’ duration, had audiometrically documented hearing loss on at least one occasion, and tinnitus or aural fullness in the treated ear, excluding other possible causes of vertigo. These factors made up a diagnosis of definite unilateral or bilateral Meniere’s disease, fulfilling the criteria of the 1995 American Academy of Otolaryngology-Head and Neck Surgery (AAO-HNS) guideline. Furthermore, patients had to be in an active phase of the disease, with at least two vertigo attacks per month in at least three consecutive months before enrolment. Female patients of childbearing potential were only included if they had a negative serum pregnancy test within seven days before initiation of treatment and were willing to practice acceptable methods of birth control during treatment and for three months after treatment.  **Group 1:** Low-dose Bethahistine  **Group 2:** High-dose Bethahistine  **Group 3:** Placebo  **Details of treatment protocol**: Patients were instructed to take six capsules per day. In the low-dose group (one betahistine capsule and one placebo capsule in the morning, two placebo capsules at noon, and one betahistine capsule together with one placebo capsule in the evening), for high-dose group (two capsules in the morning, two at noon, and two in the evening) and for placebo group (two capsules three times a day for nine months; an identically appearing capsule filled with mannitol and aerosil but not containing any active ingredient was administered as placebo). The first drug intake started as soon as possible after receipt of the study medication kits containing the vials during the baseline visit. Patients given a high-dose of betahistine dihydrochloride (Vasomotal, manufactured by Abbott Pharma, Hannover, Germany) 24mg was administered orally two times each day to the low-dose group.  Patients in high-dose group were given betahistine dihydrochloride (Vasomotal, manufactured by Abbott Pharma, Hannover, Germany) 2×24 mg three times each day. |
| **Morales-Luckie (2005)**(41)  **High Risk of Bias**  12 months after treatment | **Inclusion criteria:** All patients included in this study had severe disability (Scale 3) and limited vertigo control (Class C) and rejected any surgical management. Their clinical evolution of Meniere’s disease was similar, and their vertigo was under poor control by maintenance treatment. Based on the author response about definition of MD: MD was defined as an idiopathic syndrome of endolymphatic hydrops characterized by two or more spontaneous episodes of vertigo (20 min or more) associated with sensorineural hearing loss (fully documented by audiometry) and tinnitus or aural fullness with the absent of other possible causes.  **Group 1:** Oral Prednisolone: [Prednisolone + diphenidol + acetazolamide + low-sodium diet (< 1,500 mg/d)]  **Group 2:** maintenance therapy: diphenidol + acetazolamide + low-sodium diet (< 1,500 mg/d)  **Details of treatment protocol**: Patients received diphenidol 25 mg/d, acetazolamide 250 mg/48 h orally, and a low-sodium diet (,1,500 mg/d). In addition, reductions in consumption of alcohol, caffeine, nicotine, and stress were also suggested plus prednisone (0.35 mg/kg/d) for 18 weeks. |

# Summarized Risk of Bias Assessment

The most common reasons for an overall assessment of high risk of bias were related to the domains of attrition bias, performance bias and detection bias. Of the nine studies at high risk of bias, six were rated as such for attrition bias (28,29,32,35,38,42), five for detection bias (28,29,37,38,50), four for performance bias (29,37,38,41), two for selection bias (28,38), and two for selective outcome reporting and other bias (29,38).

The most common reasons for overall assessment of unclear risk of bias were linked with the domains of selection bias, other bias and detection bias. For some studies there was a lack of clarity as to whether sequence generation and allocation concealment were carried out, who assessed the outcomes and if the assessors were blinded to the study intervention, whether a study was funded by industry, and whether the authors had any conflict of interest. Due to insufficient information, an unclear risk of bias rating was applied in nine studies for each of the selection bias (29,30,32,33,37,41–44) and other bias (30,33–35,38,41–44) domains, in six studies for the detection bias domain (30–32,41,42,44), in three studies for the attrition bias domain (34,39,44), in one study for the performance bias domain(44), and in one study for the selective outcome reporting (43). Only two of the included 16 trials referenced a priori protocol to verify a priori outcomes against the reported ones (31,34). Two trials that were not included in the synthesis were assessed to be at high risk of bias due to concerns in selection bias and attrition bias (36,40). Further details on the risk of bias assessment for these two trials are presented in Appendix 3 (Table A4b).

**Table A3**-Risk of bias assessment for the 16 studies included in the analyses

| **Domain** | **Support for judgment** | | | **Review authors’ judgment** | | **Overall Study Risk** | | | |  |  |
| --- | --- | --- | --- | --- | --- | --- | --- | --- | --- | --- | --- |
| **Bojrab 2018** (28) | | | | | | Low (vertigo, THI, DHI, QoL)  High (PTA) | | | |  |  |
| **Selection bias** |  | | |  | | |  | | |  |  |
| Random sequence generation | It seems that they just alternated allocation without actual randomization process. Each surgeon alternated between ESD with injection of steroid and ESD without injection, such that each surgeon approximately had an equal number of patients in each group. The patients were blinded as to whether they received the steroid injection intraoperatively. If a surgeon was unable to definitively identify the endolymphatic sac, then the patient was not injected and was placed in the control group. | | | High | | |  |  |  |  |  |
| Allocation concealment | See above reasoning for ‘Random sequence generation’. It seems that surgeons knew and decided on allocation. | | | High | | |  |  |  |  |  |
| **Performance bias** |  | | |  | | |  |  |  |  |  |
| Blinding of participants and personnel | Patients were blinded but not the surgeon. However, blinding of surgeon was not practical. If a surgeon was unable to definitively identify the endolymphatic sac, then the patient was not injected and was placed in the control group. | | | Low | | |  |  |  |  |  |
| **Detection bias** |  | | |  | | |  |  |  |  |  |
| Blinding of outcome assessment | Single blinded study in which only patients were blinded. High risk for PTA because only patients were blinded not the assessor who administered the PTA. But low risk for other outcomes (vertigo, THI, DHI, QoL) because these outcomes were self-reported via patients who responded to questionnaires questions. | | | Low (vertigo, THI, DHI, QoL)  High (PTA) | | |  |  |  |  |  |
| **Attrition bias** |  | | |  | | |  |  |  |  |  |
| Incomplete outcome data | 10 patients (28%) were lost to follow up (unclear which group they belong to). 4 patients (11%), two patients in each of the two groups, were treatment failures because they received another intervention such as intratympanic gentamycin injection, vestibular nerve section, or labyrinthectomy.  The numbers of patient contributed data in different outcomes varies and some outcomes don’t have data from all patients. For example, one of the patients did not complete the questionnaires at the 24-month mark. | | | High | | |  |  |  |  |  |
| **Reporting bias** |  | | |  | | |  |  |  |  |  |
| Selective reporting | Protocol is not provided but the study has reported most of the expected outcomes | | | Low | | |  |  |  |  |  |
| **Other bias** |  | | |  | | |  |  |  |  |  |
| Other sources of bias | Non-industry funding, authors declared no conflict of interest | | | Low | | |  |  |  |  |  |
| **Outcomes** | Vertigo control, PTA, DHI, THI, QoL | | |  | | |  | | |  |  |
| **Masoumi 2017** (29) | | | | | | | High | | |  |  |
| **Selection bias** |  | | |  | | |  | | |  |  |
| Random sequence generation | Eighty patients were randomly assigned to two groups using block randomization (N1=N2=40). | | | Low | | |  |  |  |  |  |
| Allocation concealment | Insufficient information | | | Unclear | | |  |  |  |  |  |
| **Performance bias** |  | | |  | | |  |  |  |  |  |
| Blinding of participants and personnel | Not blinded | | | High | | |  |  |  |  |  |
| **Detection bias** |  | | |  | | |  |  |  |  |  |
| Blinding of outcome assessment | Not blinded | | | High | | |  |  |  |  |  |
| **Attrition bias** |  | | |  | | |  |  |  |  |  |
| Incomplete outcome data | 10-17.5% of patients were lost to follow up. There was no information if the traits and reasons for lost patients were balanced across groups. | | | High | | |  |  |  |  |  |
| **Reporting bias** |  | | |  | | |  |  |  |  |  |
| Selective reporting | The registry record reports to tinnitus as a secondary outcome, but the authors didn't report it in this manuscript. | | | High | | |  |  |  |  |  |
| **Other bias** |  | | |  | | |  |  |  |  |  |
| Other sources of bias | The study was funded by non-industry (supported by Tehran University Medical Sciences) organization; no information on conflict of interest was provided; some information in the protocol differs than what is reported in the manuscript. The number of subjects 81 (41 in each group) in the registry but 80 in the manuscript. The last follow up is 12 months but data on 12 months is not reported. | | | High | | |  |  |  |  |  |
| **Outcomes** | Vertigo control and hearing level | | |  | | |  | | |  |  |
| **Paragache 2005** (30) | | | | | | | Unclear | | |  |  |
| **Selection bias** |  | | |  | | |  | | |  |  |
| Random sequence generation | No information was provided. | | | Unclear | | |  |  |  |  |  |
| Allocation concealment | No information was provided. | | | Unclear | | |  |  |  |  |  |
| **Performance bias** |  | | |  | | |  |  |  |  |  |
| Blinding of participants and personnel | No information was provided; however, blinding was infeasible since two different method of administration (IT vs oral). | | | Low | | |  |  |  |  |  |
| **Detection bias** |  | | |  | | |  |  |  |  |  |
| Blinding of outcome assessment | No information was provided. | | | Unclear | | |  |  |  |  |  |
| **Attrition bias** |  | | |  | | |  |  |  |  |  |
| Incomplete outcome data | No missing data. | | | Low | | |  |  |  |  |  |
| **Reporting bias** |  | | |  | | |  |  |  |  |  |
| Selective reporting | no protocol or registry reported; however, common expected outcomes were reported: PTA, vertigo, SDS, tinnitus, aural fullness | | | Low | | |  |  |  |  |  |
| **Other bias** |  | | |  | | |  |  |  |  |  |
| Other sources of bias | No information on funding and, conflict of interest was reported. | | | Unclear | | |  |  |  |  |  |
| **Outcomes** | Vertigo and hearing, speech discrimination score, tinnitus, aural fullness | | |  | | |  | | |  |  |
| **Patel 2016** (31) | | | | | | | **Unclear** (Vertigo, AEs, DHI, THI, FLS)  **Low** (PTA, SDS) | | |  |  |
| **Selection bias** |  | | |  | | |  | | |  |  |
| Random sequence generation | The double-blind randomization sequence was generated by constructing 15 blocks of four possible combinations. A technical engineer outside the trial team constructed this sequence. | | | Low | | |  |  |  |  |  |
| Allocation concealment | The allocation was central (pharmacy), “The randomization sequence was allocated in numerical order, retained and concealed by the Charing Cross Hospital and Leicester Royal Infirmary pharmacy aseptic units who prepared each injection in unmarked 1 mL glass syringes and documented the drug history of each patient. The pharmacy units assigned participants to interventions but did not reveal the drug sequence to any member of the trial team.”. | | | Low | | |  |  |  |  |  |
| **Performance bias** |  | | |  | | |  |  |  |  |  |
| Blinding of participants and personnel | All investigators and patients were masked to treatment allocation. | | | Low | | |  |  |  |  |  |
| **Detection bias** |  | | |  | | |  |  |  |  |  |
| Blinding of outcome assessment | Double blind study but it is unclear who assessed the outcomes (various outcomes) although for audiograms (PTA) only pharmacy knew the outcomes and the rest of patients and investigators were masked. | | | Unclear (Vertigo, AEs, DHI, THI, FLS  Low (PTA, SDS) | | |  |  |  |  |  |
| **Attrition bias** |  | | |  | | |  |  |  |  |  |
| Incomplete outcome data | Both ITT and per protocol analyses were reported. There were two lost/withdraw (4%) in one arm and two crossed over in the other arm. The number of lost/cross over were balanced across groups. | | | Low | | |  |  |  |  |  |
| **Reporting bias** |  | | |  | | |  |  |  |  |  |
| Selective reporting | Protocol exists (ClinicalTrials.gov, number NCT00802529) and prespecified outcomes are reported in the manuscript. | | | Low | | |  |  |  |  |  |
| **Other bias** |  | | |  | | |  |  |  |  |  |
| Other sources of bias | It is funded by non-industry. One investigator declared that is involved in an industry funded trial. | | | Low | | |  |  |  |  |  |
| **Outcomes** | Vertigo, PTA, SDS, AEs, DHI, THI, FLS | | |  | | |  | | |  |  |
| **Kitahara 2016** (32) | | | | | | | High | | |  |  |
| **Selection bias** |  | | |  | | |  | | |  |  |
| Random sequence generation | Computer-generated block randomization | | | Low | | |  |  |  |  |  |
| Allocation concealment | Insufficient information | | | Unclear | | |  |  |  |  |  |
| **Performance bias** |  | | |  | | |  |  |  |  |  |
| Blinding of participants and personnel | Open label study, however, concealment was not feasible (surgery vs medication). Single blinded according to the trial's register information but that may refer to another subgroup/companion study | | | Low | | |  |  |  |  |  |
| **Detection bias** |  | | |  | | |  |  |  |  |  |
| Blinding of outcome assessment | Single blinded according to the trial's registry information but that may refer to another subgroup/companion study. Unclear who assessed the outcomes. | | | Unclear | | |  |  |  |  |  |
| **Attrition bias** |  | | |  | | |  |  |  |  |  |
| Incomplete outcome data | 4-13 (17%) lost (unbalanced across arms, no information about their characteristics and on how they were treated). | | | High | | |  |  |  |  |  |
| **Reporting bias** |  | | |  | | |  |  |  |  |  |
| Selective reporting | No protocol was available but most of the expected outcomes were reported | | | Low | | |  |  |  |  |  |
| **Other bias** |  | | |  | | |  |  |  |  |  |
| Other sources of bias | No conflict of interest, funder had no role in study design, analyses etc. | | | Low | | |  |  |  |  |  |
| **Outcomes** | Vertigo, hearing, SDS, SRS | | |  | | |  | | |  |  |
| **Albu 2016** (33) | | | | | | | Unclear | | |  |  |
| **Selection bias** |  | | |  | | |  | | |  |  |
| Random sequence generation | Computer-generated list of random numbers | | | Low | | |  |  |  |  |  |
| Allocation concealment | Insufficient information | | | Unclear | | |  |  |  |  |  |
| **Performance bias** |  | | |  | | |  |  |  |  |  |
| Blinding of participants and personnel | Both the surgeons and the patients were blinded to the treatment. | | | Low | | |  |  |  |  |  |
| **Detection bias** |  | | |  | | |  |  |  |  |  |
| Blinding of outcome assessment | Audiometric testing and completion of questionnaires were performed by researchers blinded to the surgeons. | | | Low | | |  |  |  |  |  |
| **Attrition bias** |  | | |  | | |  |  |  |  |  |
| Incomplete outcome data | 4.8% lost to follow up (3 patients, 1 in group A and 2 in group B, no further info on the cause). | | | Low | | |  |  |  |  |  |
| **Reporting bias** |  | | |  | | |  |  |  |  |  |
| Selective reporting | No protocol is available but most expected outcomes are reported. | | | Low | | |  |  |  |  |  |
| **Other bias** |  | | |  | | |  |  |  |  |  |
| Other sources of bias | No funding but no data on conflict of interest | | | Unclear | | |  |  |  |  |  |
| **Outcomes** | Vertigo, hearing, SDS, THI, FLS, Tinnitus | | |  | | |  | | |  |  |
|  |  | | |  | | |  | | |  |  |
| **Albu 2015** (35) | | | | | | | High | | |  |  |
| **Selection bias** |  | | |  | | |  | | |  |  |
| Random sequence generation | Computer generated number sequence of 0 (Group A) and 1 (Group B) number sets indicating whether the patient was allocated to Group A or B. | | | Low | | |  |  |  |  |  |
| Allocation concealment | Randomization was achieved by one investigator only (FC) one day before the injection procedure. Both the surgeons (SA, GB and FT) and the patients were blinded to the treatment. | | | Low | | |  |  |  |  |  |
| **Performance bias** |  | | |  | | |  |  |  |  |  |
| Blinding of participants and personnel | Both patients and surgeons were blinded | | | Low | | |  |  |  |  |  |
| **Detection bias** |  | | |  | | |  |  |  |  |  |
| Blinding of outcome assessment | Audiometric testing and completion of questionnaires were performed by different researchers (FC, VT, LM, AN) blinded to the surgeons. | | | Low | | |  |  |  |  |  |
| **Attrition bias** |  | | |  | | |  |  |  |  |  |
| Incomplete outcome data | 10.6% (7 patients (3 in Group A and 4 in Group B) were excluded because of insufficient compliance.) 3 vs 4 is almost balanced across groups and reason was the same in both groups. They just excluded these patients and didn't do any amputation, ITT or per protocol to know the difference etc. No info about the characteristics of these patients provided. | | | High | | |  |  |  |  |  |
| **Reporting bias** |  | | |  | | |  |  |  |  |  |
| Selective reporting | No protocol or registry record; however, has reported most of the common outcomes | | | Low | | |  |  |  |  |  |
| **Other bias** |  | | |  | | |  |  |  |  |  |
| Other sources of bias | No funding, but no information on conflict of interest | | | Unclear | | |  |  |  |  |  |
| **Outcomes** | Vertigo, hearing, AEs, FLS, SDS | | |  | | |  | | |  |  |
|  |  | | |  | | |  | | |  |  |
| **Saliba 2015** (50) | | | | | | | High | | |  |  |
| **Selection bias** |  | | |  | | |  | | |  |  |
| Random sequence generation | It was a simple nonblinded randomization using the coin toss method. | | | Low | | |  |  |  |  |  |
| Allocation concealment | The patient and the investigator are unaware of the group to which a participant will be allocated. | | | Low | | |  |  |  |  |  |
| **Performance bias** |  | | |  | | |  |  |  |  |  |
| Blinding of participants and personnel | Patients and doctors were unblinded, but it may not affect patients given they won't seek another surgery (co-intervention) | | | Low | | |  |  |  |  |  |
| **Detection bias** |  | | |  | | |  |  |  |  |  |
| Blinding of outcome assessment | It was an unblinded study, but blinding was feasible at least in patients and outcome assessors. Both subjects and outcome assessors knew the allocation. It is unclear who assessed the outcomes. If most were reported by subjects, PTA and SDS would be assessed by a health professional, so both parties could be blinded to the allocation. | | | High | | |  |  |  |  |  |
| **Attrition bias** |  | | |  | | |  |  |  |  |  |
| Incomplete outcome data | All included participants in each arm were available for analysis, no lost or drop out. | | | Low | | |  |  |  |  |  |
| **Reporting bias** |  | | |  | | |  |  |  |  |  |
| Selective reporting | No protocol or registry record but reported most common outcomes. | | | Low | | |  |  |  |  |  |
| **Other bias** |  | | |  | | |  |  |  |  |  |
| Other sources of bias | no funding, no conflict of interest | | | Low | | |  |  |  |  |  |
| **Outcomes** | Vertigo, hearing, tinnitus, aural fullness, SDS | | |  | | |  | | |  |  |
| **Casani 2012** (37) | | | | | | | High | | |  |  |
| **Selection bias** |  | | |  | | |  | | |  |  |
| Random sequence generation | A computer-generated list of random numbers was used. | | | Low | | |  |  |  |  |  |
| Allocation concealment | insufficient information | | | Unclear | | |  |  |  |  |  |
| **Performance bias** |  | | |  | | |  |  |  |  |  |
| Blinding of participants and personnel | Unblinded study. Two different regimens were given to the patients, so they could have blinded the participant and personnel to the interventions since both were administered intratympanically. | | | High | | |  |  |  |  |  |
| **Detection bias** |  | | |  | | |  |  |  |  |  |
| Blinding of outcome assessment | Unblinded study and subjective outcomes. | | | High | | |  |  |  |  |  |
| **Attrition bias** |  | | |  | | |  |  |  |  |  |
| Incomplete outcome data | 5% (3 patients 1 vs 2) were not included in the analysis due to failure of the treatments and were treated with an alternative therapy. | | | Low | | |  |  |  |  |  |
| **Reporting bias** |  | | |  | | |  |  |  |  |  |
| Selective reporting | No protocol or registry referenced; however, most of the common outcomes were reported: vertigo, PTA, SDS, FLS, AEs (tympanic membrane perforation) | | | Low | | |  |  |  |  |  |
| **Other bias** |  | | |  | | |  |  |  |  |  |
| Other sources of bias | No conflict of interest and no funding | | | Low | | |  |  |  |  |  |
| **Outcomes** | Vertigo, PTA, SDS, FLS, AEs (residual tympanic membrane perforation) | | |  | | |  | | |  |  |
| **Ganança 2009** (38) | | | | | | | High | | |  |  |
| **Selection bias** |  | | |  | | |  | | |  |  |
| Random sequence generation | From the description, it doesn’t seem to a proper randomization. They allocated the first consecutive patients to 16mg group and the next 60 patients to the 24mg group as it reads in the article, "This was a randomized, open-label study comparing betahistine 16 mg tid or betahistine 24 mg bid taken orally over 24 weeks. Sixty consecutive patients fulfilling the inclusion/exclusion criteria received betahistine 16 mg tid and the next 60 consecutive patients received betahistine 24 mg bid." | | | High | | |  |  |  |  |  |
| Allocation concealment | It was clear which patients goes to each arm (allocation seems unconcealed) as it reads in the article, “This was a randomized, open-label study comparing betahistine 16 mg tid or betahistine 24 mg bid taken orally over 24 weeks. Sixty consecutive patients fulfilling the inclusion/exclusion criteria received betahistine 16 mg tid and the next 60 consecutive patients received betahistine 24 mg bid.". | | | High | | |  |  |  |  |  |
| **Performance bias** |  | | |  | | |  |  |  |  |  |
| Blinding of participants and personnel | It was an open label study while blinding was feasible. | | | High | | |  |  |  |  |  |
| **Detection bias** |  | | |  | | |  |  |  |  |  |
| Blinding of outcome assessment | It was an open label study, so outcome assessors would not be blinded. | | | High | | |  |  |  |  |  |
| **Attrition bias** |  | | |  | | |  |  |  |  |  |
| Incomplete outcome data | They seem to have excluded non-adherence without imputation, ITT etc. as it reads, "Adults completing 4, 12 and 24 weeks of continuous oral treatment with betahistine 16 mg tid or oral betahistine 24 mg bid were admitted. Patients who were receiving other anti-vertigo drugs or other therapy for Meniere’s disease or who failed to complete all three phases of continuous treatment with betahistine 16 mg tid or betahistine 24 mg bid over 24 weeks were excluded." It is unclear how many patients were in such category and they were totally not accounted for. The study reports 120 participants (60 in each arm) in the analyses. However, they don't mention how many patients were included in the randomization after which the ones that, "failed to complete all three phases of continuous treatment with betahistine 16 mg tid or betahistine 24 mg bid over 24 weeks were excluded". It is suspected that the number of patients were more than 120 but only the ones that completed all three phases of continuous treatment were included in the analyses. If the total number of patients were 120 from the beginning, and all are included in the analyses, then who are the ones who didn't complete all 3 timepoints and were excluded? Given treatment is given after randomization, these excluded patients were included in randomization and study but not counted in the analyses. | | | High | | |  |  |  |  |  |
| **Reporting bias** |  | | |  | | |  |  |  |  |  |
| Selective reporting | No protocol or registry was referenced. Only vertigo was reported. No data on common outcomes such as hearing level, tinnitus, SDS, aural fullness etc. | | | High | | |  |  |  |  |  |
| **Other bias** |  | | |  | | |  |  |  |  |  |
| Other sources of bias | There was no information about funding. The authors alone are responsible for the content and writing of the paper. | | | Unclear | | |  |  |  |  |  |
| **Outcomes** | Vertigo | | |  | | |  | | |  |  |
| **Postema 2008** (39) | | | | | | | Low (for outcomes collected before 12 months which was perhaps the final visit)  Unclear (for outcomes collected at 12 months which was the final visit perhaps) | | |  |  |
| **Selection bias** |  | | |  | | |  | | |  |  |
| Random sequence generation | Insufficient information | | | Unclear | | |  |  |  |  |  |
| Allocation concealment | Before the randomization code was broken in the final visit, only known to one of the hospital pharmacists. It seems to have been a central allocation. | | | Low | | |  |  |  |  |  |
| **Performance bias** |  | | |  | | |  |  |  |  |  |
| Blinding of participants and personnel | During the final visit the double-blind code was broken. Before that time this code was only known to one of the hospital pharmacists. It is not clear if the data collected during the final visit was collected in unblinded manner or not. Low risk of bias for outcomes before 12 months which was the final visit perhaps. Unclear risk of bias for outcomes collected during the final visit. | | | Low (for outcomes collected before 12 months which was perhaps the final visit)  Unclear (for outcomes collected at 12 months which was the final visit perhaps) | | |  |  |  |  |  |
| **Detection bias** |  | | |  | | |  |  |  |  |  |
| Blinding of outcome assessment | During the final visit the double-blind code was broken. Before that time this code was only known to one of the hospital pharmacists. It is not clear if the data collected during the final visit was collected in unblinded manner or not. Low risk of bias for outcomes before 12 months which was the final visit perhaps. Unclear risk of bias for outcomes collected during the final visit. | | | Low (for outcomes collected before 12 months which was perhaps the final visit)  Unclear (for outcomes collected after 12 months which was the final visit perhaps) | | |  |  |  |  |  |
| **Attrition bias** |  | | |  | | |  |  |  |  |  |
| Incomplete outcome data | Of the 28 patients in the study, 12 were treated with placebo and 16 with gentamicin" but in none of the figures (1-4) the number of subjects exceeds 10. The study reports that a complete dataset could not be obtained for two subjects in the placebo group. However, it is unclear why the number of subjects is only from 1-10 in most figures and what was done about the 2 subjects in placebo group that didn’t have complete data set. | | | Unclear | | |  |  |  |  |  |
| **Reporting bias** |  | | |  | | |  |  |  |  |  |
| Selective reporting | No protocol or registry is referenced but it has reported most common outcomes | | | Low | | |  |  |  |  |  |
| **Other bias** |  | | |  |  | | | | | | |
| Other sources of bias | No information on funding and conflict of interest was reported. | | | Unclear |  |  |  |  |  |  |  |
| **Outcomes** | Vertigo, hearing, tinnitus, aural fullness | | |  |  | | | | | | |
|  | | | | | | | High | | |  |  |
| **Selection bias** |  | | |  | | |  | | |  |  |
| Random sequence generation |  | | |  | | |  |  |  |  |  |
| Allocation concealment |  | | |  | | |  |  |  |  |  |
| **Performance bias** |  | | |  | | |  |  |  |  |  |
| Blinding of participants and personnel |  | | |  | | |  |  |  |  |  |
| **Detection bias** |  | | |  | | |  |  |  |  |  |
| Blinding of outcome assessment |  | | |  | | |  |  |  |  |  |
| **Attrition bias** |  | | |  | | |  |  |  |  |  |
| Incomplete outcome data |  | | |  | | |  |  |  |  |  |
| **Reporting bias** |  | | |  | | |  |  |  |  |  |
| Selective reporting |  | | |  | | |  |  |  |  |  |
| **Other bias** |  | | |  | | |  |  |  |  |  |
| Other sources of bias |  | | |  | | |  |  |  |  |  |
| **Outcomes** |  | | |  | | |  | | |  |  |
| **Morales-Luckie 2005** (41) | | | | | | | High | | |  |  |
| **Selection bias** |  | | |  | | |  | | |  |  |
| Random sequence generation | Minimization was used. | | | Low | | |  |  |  |  |  |
| Allocation concealment | Insufficient information | | | Unclear | | |  |  |  |  |  |
| **Performance bias** |  | | |  | | |  |  |  |  |  |
| Blinding of participants and personnel | No information on blinding was available but the study doesn't report any placebo pill for the group that didn't receive steroid. | | | High | | |  |  |  |  |  |
| **Detection bias** |  | | |  | | |  |  |  |  |  |
| Blinding of outcome assessment | insufficient information | | | Unclear | | |  |  |  |  |  |
| **Attrition bias** |  | | |  | | |  |  |  |  |  |
| Incomplete outcome data | All included participants were included in the analysis | | | Low | | |  |  |  |  |  |
| **Reporting bias** |  | | |  | | |  |  |  |  |  |
| Selective reporting | No protocol or registration provided to check against, but the manuscript has reported common expected outcomes: PTA, vertigo, Aural fullness, Tinnitus, QoL | | | Low | | |  |  |  |  |  |
| **Other bias** |  | | |  |  | | |  |  |  |  |
| Other sources of bias | No information about funding and conflict of interest | | | Unclear |  |  |  |  |  |  |  |
| **Outcomes** | PTA, Vertigo, Aural fullness, Tinnitus, AEs (withdrawal) | | |  |  | | |  |  |  |  |
| **Garduño-Anaya 2005** (42) | | | | | | | High | | |  |  |
| **Selection bias** |  | | |  | | |  | | |  |  |
| Random sequence generation | Insufficient info | | | Unclear | | |  |  |  |  |  |
| Allocation concealment | Insufficient info | | | Unclear | | |  |  |  |  |  |
| **Performance bias** |  | | |  | | |  |  |  |  |  |
| Blinding of participants and personnel | Both patient and physician were blinded. | | | Low | | |  |  |  |  |  |
| **Detection bias** |  | | |  | | |  |  |  |  |  |
| Blinding of outcome assessment | It says double blind (Neither patient nor physician knew the contents of the injection) but not clear if all outcome assessors at all time points were blinded. It is not reported who administered the questionnaires and tests and if they were blinded. | | | Unclear | | |  |  |  |  |  |
| **Attrition bias** |  | | |  | | |  |  |  |  |  |
| Incomplete outcome data | 45% of patients in one arm were classified as failure and given another treatment but 36% were excluded from the analyses (7 included out of 11). It reads, "In the dexamethasone group all 11 patients were applied the data analysis at 2 years of follow-up. In the control group at 6 months of follow-up 2 patients were classified as failure and given another treatment, at 18 months of follow-up another 2 patients were classified as failure and given another treatment, and at 24 months of follow-up 1 patient have to be given another treatment due to being classified as failure. So, at the end of this 2-year follow-up study to analyze data between groups we considered the 11 patients in the dexamethasone group and 7 in the control group. It is worth mentioning that the control figures were realized after removal of 4 patients, and at the end of the 2-year follow-up they were statistically analyzed as missing values.". | | | High | | |  |  |  |  |  |
| **Reporting bias** |  | | |  | | |  |  |  |  |  |
| Selective reporting | No protocol or registry was referenced. However, it reports most common expected outcomes: vertigo, hearing, tinnitus, FLS, DHI | | | Low | | |  |  |  |  |  |
| **Other bias** |  | | |  | | |  |  |  |  |  |
| Other sources of bias | No information on funding and conflict of interest | | | Unclear | | |  |  |  |  |  |
| **Outcomes** | Vertigo, hearing, tinnitus, FLS, DHI | | |  | | |  | | |  |  |
| **Stokroos and Kingma 2004** (43) | | | | | | | Unclear | | |  |  |
| **Selection bias** |  |  | | | | | | | |  |  |
| Random sequence generation | Insufficient information | | | Unclear | | | | | |  |  |
| Allocation concealment | Randomization was performed by the hospital pharmacist, who was the only person who knew whether placebo or gentamicin was given before the end of the study period. | | | Low | | | | | |  |  |
| **Performance bias** |  | |  | | | | | | |  |  |
| Blinding of participants and personnel | Double blind study, only pharmacist knew which drug was given before the end of the study. | | |  | | | | | |  |  |
| **Detection bias** |  | | |  | | | | | |  |  |
| Blinding of outcome assessment | Double blind study, only pharmacist knew which drug was given before the end of the study. | | | Low | | | | | |  |  |
| **Attrition bias** |  | | |  | | | | | |  |  |
| Incomplete outcome data | Complete data. | | | Low | | | | | |  |  |
| **Reporting bias** |  | | |  | | | | | |  |  |
| Selective reporting | No protocol and no registry were referenced. The study only reported two outcomes: vertigo, hearing | | | Unclear | | | | | |  |  |
| **Other bias** |  | | |  | | | | | |  |  |
| Other sources of bias | No information on funding and conflict of interest was reported. | | | Unclear | | | | | |  |  |
| **Outcomes** | vertigo, hearing | | |  | | | | | |  |  |
| **ElBeltagy 2012** (44) | | | | | | | Unclear | | |  |  |
| **Selection bias** |  | | |  | | | | | |  |  |
| Random sequence generation | Insufficient information | | | Unclear | | | | | |  |  |
| Allocation concealment | Insufficient information | | | Unclear | | | | | |  |  |
| **Performance bias** |  | | |  | | | | | |  |  |
| Blinding of participants and personnel | Insufficient information | | | Unclear | | | | | |  |  |
| **Detection bias** |  | | |  | | | | | |  |  |
| Blinding of outcome assessment | Insufficient information | | | Unclear | | | | | |  |  |
| **Attrition bias** |  | | |  | | | | | |  |  |
| Incomplete outcome data | There is 13.3% loss but balanced across groups (13/15). The 2 in group I were in Class A (complete recovery) and the 2 in group II were in group B (substantial improvement). No reason is reported for lost to follow up of these 4 patients (2 in each arm). | | | Unclear | | | | | |  |  |
| **Reporting bias** |  | | |  | | | | | |  |  |
| Selective reporting | No protocol or registry referenced. However, common expected outcomes are reported: PTA, vertigo, SDS, Tinnitus, DHI, aural fullness | | | Low | | | | | |  |  |
| **Other bias** |  | | |  | | | | | |  |  |
| Other sources of bias | Authors declared no conflict of interest; however, no information on funding was available. | | | Unclear | | | | | |  |  |
| **Outcomes** | PTA, vertigo, SDS, Tinnitus, DHI, aural fullness | | |  | | | | |  | |  |

Abbreviations: AEs= Adverse Events; DHI= Dizziness Handicap Inventory; FLS=Functional Level Scale/Score; IT= Intratympanic; ITT= Intention-To-Treat; MiniTF12= Mini-Tinnitus impairment questionnaire score based on 12 items; PTA= Pure Tone Audiometry; QoL= Quality of Life; SDS= Speech Discrimination Score; SDS⃰= Self-rating Depression Scale; SRS= Stress Response Scale-18; THI=Tinnitus Handicap Inventory; VDADL= Vestibular Disorders Activities of Daily Living

**Details on the two studies excluded from the analyses**:

Two studies were not included in the synthesis because one (40) was not deemed as randomized although it was labelled as such, and the other (36) was terminated early before reaching its full inclusion resulting in too small sample size (15 in total, 4, 5 and 5 per arm) to support the validity of the results (36). Further details with regards to each study are presented in **Table A4a and b**.

**Table A4a**: Characteristics of two excluded studies from synthesis

| **Author (Publication Year), Country of conduct, Funding source, RoB Assessment** | **Inclusion criteria (underlined texts denote special characteristics)**  **Interventions groups**  **Treatment protocol**  **Follow up time** | **Age in years (Mean± SD), Female gender,**  **MD duration in years,**  **MD stage** |
| --- | --- | --- |
| Kitahara (2008)(40),  Japan, Non-industry funding,  High Risk of Bias | **Inclusion criteria:** Patients were eligible for enrollment if they had received a clinical diagnosis of intractable MD according to the 1995 American Academy of Otolaryngology–Head and Neck Surgery (AAO-HNS) criteria. Exclusions were other causes and other disorders.  **Group 1** (N=100): Endolymphatic sac drainage + steroid instillation into the sac  **Group 2** (N=47): Endolymphatic sac drainage without steroid instillation into the sac  **Group 3** (N=50): Medical managements included diuretics, betahistine, diphenidol, dimenhydrinate, and diazepam  **Details of treatment protocol**: For steroid instillation into the endolymphatic sac in Group 1, endolymphatic-sac drainage and steroid-instillation surgery was performed. This surgery was designed in the same manner as the endolymphatic sac-expanding surgery in Group 2 except for the instillation of steroids into the endolymphatic sac; a simple mastoidectomy was performed, clearly exposing the endolymphatic sac in the area between the sigmoid sinus and the inferior margin of the posterior semicircular canal. If possible, the sac was exposed, including the rugose portion. The sac was opened with an L- (right ear) or backward L- (left ear) shaped incision made along the posterior and distal margins of the lateral wall. Then the sac was filled with a mass of 20 mg of prednisolone. While dissolving the mass in the sac, we prepared a bundle of absorbable gelatin films (ca. 4 20 0.7 mm 5 sheets) with fan- and stick-shaped ends. These films were tied to one another with biochemical adhesive (human thrombin combined with human fibrinogen) at the stick shaped end. The fan-shaped end was then inserted into the sac, and small pieces of absorbable gelatin sponge soaked in a high concentration of dexamethasone (32 mg/4 mL) were placed inside and outside the sac lumen expanded with the bundle. The sponges containing dexamethasone placed outside the sac were coated with the adhesive so that dexamethasone was slowly delivered into the sac over a long period of time as a natural sustained-release vehicle. The stick-shaped end extending out of the sac was fixed to the front edge of the mastoid cavity with the same adhesive so that the incision into the sac was also expanded if possible. The mastoid cavity was filled with relatively large pieces of absorbable gelatin sponge dipped in steroid antibiotic solution, after which the wound was closed with skin sutures. Patients in Group 3 consisted of 50 patients who declined endolymphatic sac drainage and thereafter continually received the best available non-surgical medical treatments mentioned above. 143  **Follow up time**: 7 years | **Group 1**: 50.3±14.5  **Group 2**: 55.6±10.1  **Group 3**: 53.7±12.4  **Group 1**:56  **Group 2**: 26  **Group 3**: 26  **Group 1**: Mean ± SD: 8.7±7.5  **Group 2**: Mean ± SD: 9.5±8.9 **Group 3**: Mean ± SD: 8.8±6.2  **Group 1**: I:4, II: 18, III: 60,  IV: 18  **Group 2**: I:3, II: 5, III: 32, IV: 7  **Group 3**: I:3, II: 8, III: 27, IV: 12 |
| Bremer (2014)(36),  the Netherlands,  No funding,  High Risk of Bias | **Inclusion criteria:** Patients diagnosed as having unilateral MD according to the 1995 AAO-HNS criteria. Other inclusion criteria were: Meniere’s disease *resistant to conservative medical treatment executed longer than 6 months*, (that is, Dizziness Handicap Score of at least 30 points) and ability to provide written informed consent. Patients had to have compromised hearing on the affected side without fluctuations.  **Group 1**(N=5): IT Gentamicin  **Group 2**(N=5): IT Gentamicin+ placebo  **Group 3**(N=4): placebo  **Details of treatment protocol**: The three groups all received four weekly intratympanic injections. One group received placebo injections (sterile NaCl 0.9% solution), the other group received two injections with gentamicin 40 mg/mL and two injections with placebo in random order, and the other group received four injections with gentamicin 40 mg/mL.84  **Follow up time**: intended 2 years but study was terminated earlier perhaps over one year but less than two years | **Group 1**: 64.5±8  **Group 2**: 72.6±5  **Group 3**: 57.3±16.7  **Group 1**: 2  **Group 2**: 0  **Group 3**: 4  **Group 1**: Median (range): 3.1 (1.1-19.6)  **Group 2**: Median (range): 3.3 (0.7-7.5)  **Group 3**: Median (range): 2.5 (0.1-18.2)  **Group 1**: NR  **Group 2**: NR  **Group 3**: NR |

**Table A4b**-Risk of bias assessment for the two studies not included in the analyses

| **Domain** | **Support for judgment** | **Review authors’ judgment** | **Overall Study Risk** |
| --- | --- | --- | --- |

| **Bremer 2014** (36) | | | High |
| --- | --- | --- | --- |
| **Selection bias** |  |  |  |
| Random sequence generation | A computer-generated list of random numbers was used | Low |  |
| Allocation concealment | Because of the slow enrollment, apparent hearing loss in one patient and new insights with respect to the benefit of ITG treatment the randomization code was broken, and we performed an unscheduled interim analysis and presented the results. | High |  |
| **Performance bias** |  |  |  |
| Blinding of participants and personnel | It is a double blind. It is known that patients were blinded but unclear if personnel or outcome assessors were blinded. | Unclear |  |
| **Detection bias** |  |  |  |
| Blinding of outcome assessment | It is a double blind. It is known that patients were blinded but unclear if personnel or outcome assessors were blinded. It is also not reported if the questionnaires on outcomes were self-administered or personnel administered. | Unclear |  |
| **Attrition bias** |  |  |  |
| Incomplete outcome data | One patient in G2 withdrew after randomization because she didn't want to risk receiving placebo. One patient died in the same group due to comorbidity. One another patient withdrew after two injections because he suffered from Tumarkins crises. It is >40% attrition in one group, 20% in another group. | High |  |
| **Reporting bias** |  |  |  |
| Selective reporting | The trial ended prematurely due to intervention inherent risk of induced hearing loss, but it does report most common outcome: vertigo, PTA, DHI and adverse effects (hearing loss). The registry data only reported DHI as the main primary outcome and no information was given regarding secondary outcomes though. | Low |  |
| **Other bias** |  |  |  |
| Other sources of bias | There was no source of funding for this study. The authors declare that they have no competing interests. | Low |  |
| **Outcomes** | Vertigo, PTA, DHI, AEs (hearing loss) |  |  |

| **Kitahara 2008** (40) | | | High |
| --- | --- | --- | --- |
| **Selection bias** |  |  |  |
| Random sequence generation | The study is labelled as randomized but there is no information on how randomization occurred. Figure 1 shows as if the unit of randomization was hospitals than individuals but there is no information if it was a cluster randomized study. The authors consider three arms to have been randomized; however, it is clearly shown that the ones who declined surgery and received medication are not randomized. They should have been included in intention to treat analyses. The medication arm is not randomized. There is no information if the two hospitals were randomized as well. It seems like a cohort study. | High |  |
| Allocation concealment | Insufficient information | Unclear |  |
| **Performance bias** |  |  |  |
| Blinding of participants and personnel | Insufficient information | Unclear |  |
| **Detection bias** |  |  |  |
| Blinding of outcome assessment | Insufficient information | Unclear |  |
| **Attrition bias** |  |  |  |
| Incomplete outcome data | It is unclear when randomization occurred and how it occurred. In one hospital it is unclear how many patients declined drainage and were sent home (was it before randomization or after, perhaps around 50 folks in this category). In the other hospital 50 who declined were kept. No information on how these patients were different. It seems as if it was two separate cohorts in a prospective cohort study. In one cohort/arm the ones that declined surgery were sent home but in the other arm/cohort they were allocated in medication arm (third arm). | High |  |
| **Reporting bias** |  |  |  |
| Selective reporting | No protocol or registry referenced. It reported the two common outcomes: vertigo and hearing loss. No information on other outcomes e.g. tinnitus, aural fullness, SDS etc. | Unclear |  |
| **Other bias** |  |  |  |
| Other sources of bias | No report of competing interest. This study was non-industry funded (supported by the Foundation from Osaka University, School of Medicine). |  |  |
| **Outcomes** | Vertigo, PTA |  |  |
