# Supplementary material for: Pharmacologic and surgical therapies for patients with Meniere’s disease: A systematic review and network meta-analysis
Source: PLoS One. 2020 Sep 1;15(9):e0237523. doi: 10.1371/journal.pone.0237523 (PMC7462264; doi:10.1371/journal.pone.0237523)
Supplement: S4 Text — (DOCX) [file pone.0237523.s004.docx]

# S4 Text: Additional Analyses and Measures of Effectiveness Reviewed

## I. Hearing changes

## 1.1 Hearing change (PTA)

**1.1.1 Pairwise meta-analysis (sensitivity analysis)**

**Figure A1** presents a sensitivity analysis assuming the correlation of 0.5 before and after intervention for the pairwise meta-analysis of hearing change.

**Figure A1:** Sensitivity analysis: difference in hearing change, where positive pure tone average (PTA) change per group indicates hearing deterioration.

* Six studies (31,33,35,37,43,44) reported the means and SDs before and after intervention but not the SDs of PTA changes. We calculated the mean PTA changes and the corresponding SDs, assuming the correlation of 0.5 before and after intervention.


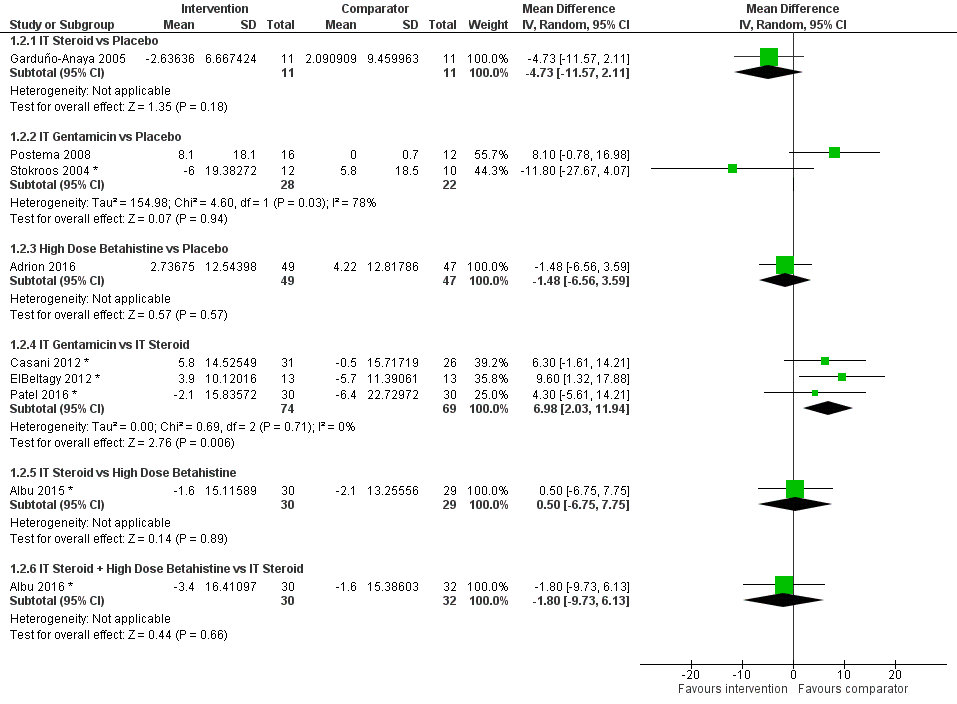


**1.1.2 More information regarding network meta-analysis of hearing change**

The following results were based on the network meta-analysis of hearing change as described in the main text, excluding Stokroos and Kingma (2004) (43) data and assuming a fixed correlation of 0.859 between pre- and post-intervention hearing levels.

**Table A5:** Summary of model fit details for the network meta-analyses of hearing change.

| **Model** | **No. of Arms / Studies** | **Total Residual Deviance** | **Deviance Information Criteria** | **Between-trial SD (95% CrI)** |
| --- | --- | --- | --- | --- |
| RE Consistency | 16 / 8 | 15.08 | 73.89 | 2.63 (0.08 to 9.63) |
| RE Unrelated Means |  | 15.73 | 76.17 | 4.76 (0.15 to 16.72) |

**Table A6:** Mean SUCRA value, mean probability to be the best, and mean rank for each treatment based on hearing change, with the treatments in descending order of mean SUCRA. These secondary measures of effect from network meta-analysis are displayed. Larger values of the mean SUCRA or the smaller values of the mean rank suggest better treatments. SUCRA: the Surface Under the Cumulative RAnking curve (SUCRA) value represents the surface underneath the cumulative ranking curve, which is the posterior probabilities for each drug to be among the n-best options.

| **Outcome: hearing change** | **Mean SUCRA** | **Mean Pr(best)** | **Mean Rank*** |
| --- | --- | --- | --- |
| **IT Steroid + Betahistine (high dose)** | 0.788 | 0.577 | 1.85 (1 to 4) |
| **Betahistine (high dose)** | 0.647 | 0.236 | 2.41 (1 to 4) |
| **IT Steroid** | 0.629 | 0.120 | 2.49 (1 to 4) |
| **Placebo** | 0.407 | 0.067 | 3.37 (1 to 5) |
| **IT Gentamicin** | 0.029 | 0.001 | 4.88 (4 to 5) |

* Mean rank with 2.5% and 97.5% quantiles in the parentheses.

**Figure A2:** The probability of each treatment to be at a specific rank for hearing change from the RE consistency model
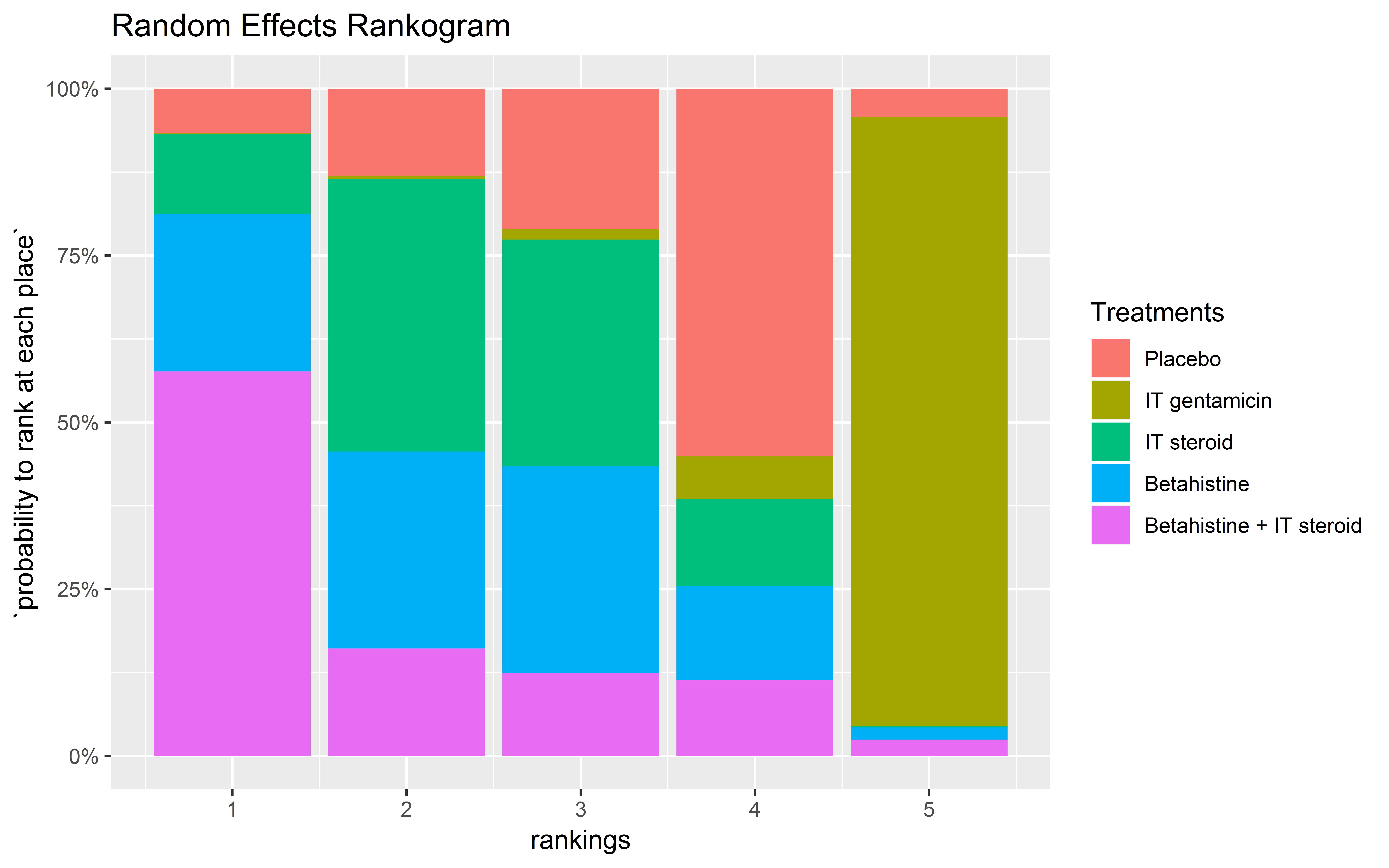
**Figure A3**: Comparison-adjusted funnel plot for hearing change
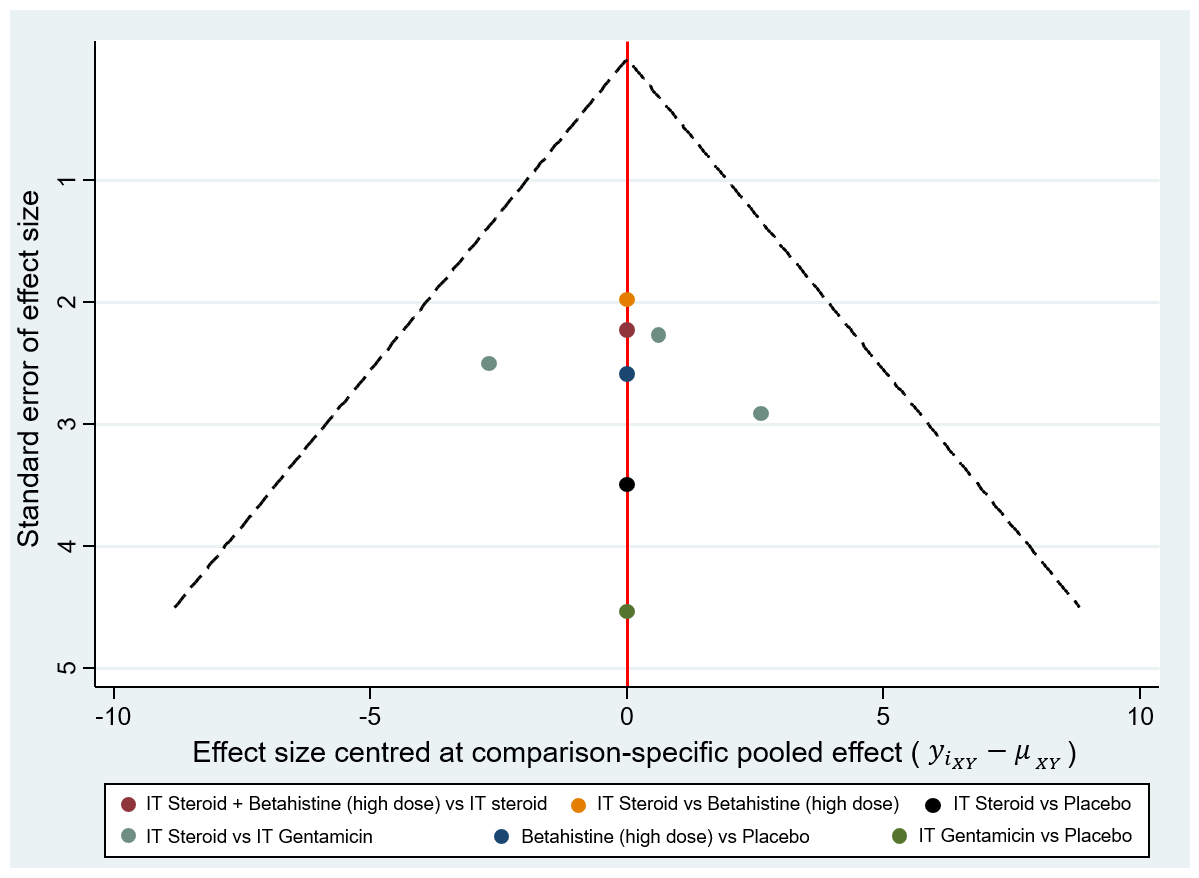


**1.1.3 Network meta-analysis (additional analysis)**

The results from an additional NMA of hearing change which included Stokroos and Kingma (2004) which considered IT gentamicin with injections six weeks apart as a separate node from IT gentamicin are displayed in **Figure A4**.

The largest difference in PTA improvement compared to placebo was associated with IT gentamicin with injections six weeks apart, estimated as -11.75 dB (95% CrI -22.93 dB to -0.65 dB), with the minus sign indicating benefit over comparator in hearing loss reduction. However, future trials are needed to confirm the advantage of IT gentamicin six weeks apart in hearing loss reduction, which was observed in a single study.

**Figure A4:** Sensitivity analysis for NMA of hearing change, which included Stokroos and Kingma (2004) and considered IT gentamicin with injections six weeks apart as a separate node from IT gentamicin. The league table presents the pairwise difference estimates in PTA change with 95% credible intervals (lower triangle), and the pairwise probabilities that a treatment is better than another (upper triangle). A complete summary of estimates for efficacy from the RE consistency model assuming vague priors is displayed. Estimates of difference in PTA change between regimens which ruled out the possibility of no difference are shown in bold, underlined font. For each comparison, the lower/right-most treatment is the reference treatment.

| **IT Gentamicin (6 wks apart)** | 0.877 | 0.939 | 0.943 | **0.991** | **0.980** |
| --- | --- | --- | --- | --- | --- |
| -8.00 (-23.29 to 7.22) | **IT Steroid + Betahistine (high dose)** | 0.667 | 0.715 | 0.972 | 0.811 |
| -9.72 (-22.82 to 3.41) | -1.70 (-12.42 to 8.97) | **Betahistine (high dose)** | 0.512 | 0.973 | 0.770 |
| -9.81 (-22.61 to 3.03) | -1.79 (-10.11 to 6.62) | -0.08 (-6.67 to 6.60) | **IT Steroid** | **0.993** | 0.763 |
| **-17.05 (-30.25 to -4.02)** | -9.06 (-18.71 to 0.48) | -7.34 (-15.13 to 0.26) | **-7.26 (-12.10 to -2.67)** | **IT Gentamicin** | 0.053 |
| **-11.75 (-22.93 to -0.65)** | -3.76 (-14.29 to 6.87) | -2.05 (-8.78 to 4.92) | -1.96 (-8.32 to 4.41) | 5.32 (-1.57 to 12.39) | **Placebo** |

## 1.2 Improved hearing >= 10 dB

Nine trials reported data on improved hearing defined as a PTA improvement ≥ 10 dB or > 15% SDS improvement (29,33,35,41) or PTA improvement ≥ 10 dB alone (30–32,37,42), and one trial did not specify any definition (28). Of these, meta-analysis was performed for the comparison between IT gentamicin and IT steroid based on two studies (31,37); findings for six pairwise comparisons of interventions were informed by data from single trials (29,30,32,33,35,42), a narrative summary was provided for one pairwise comparison due to absence of quantitative data (41) and no analysis was performed for one study due to insufficient information regarding the number of patients beyond baseline (28). **Figure A5** presents the effect estimates (risk ratio) for improved hearing >=10 dB based on single studies and meta-analysis. Cautions should be exercised regarding the interpretations given the small number of trials, most of which did not have enough sample size to ensure adequate statistical power. Findings from pairwise comparisons of interventions are as follows:

IT Steroid versus Placebo: IT steroid (dexamethasone) was associated with a marginally higher proportion of patients who had improved hearing >=10 dB than placebo, however the difference was not statistically significant (RR 3.00, 95% CI 0.37, 24.58; N=22) based on a single small trial (42).

IT Gentamicin versus IT Steroid: Based on findings from meta-analysis of two trials (31,37), there was no statistically significant difference between IT steroid and IT gentamicin for improved hearing >=10 dB (RR 0.56, 95% CI 0.08 to 4.11; N=118).

IT Steroid versus High-dose Betahistine: The RR between IT steroid (dexamethasone) and high-dose betahistine for improved hearing >= 10 dB was not statistically significant (RR 1.93, 95% CI 0.38 to 9.76; N=59) according to one study (35).**Figure A5:** Risk ratio of improved hearing >= 10 dB.


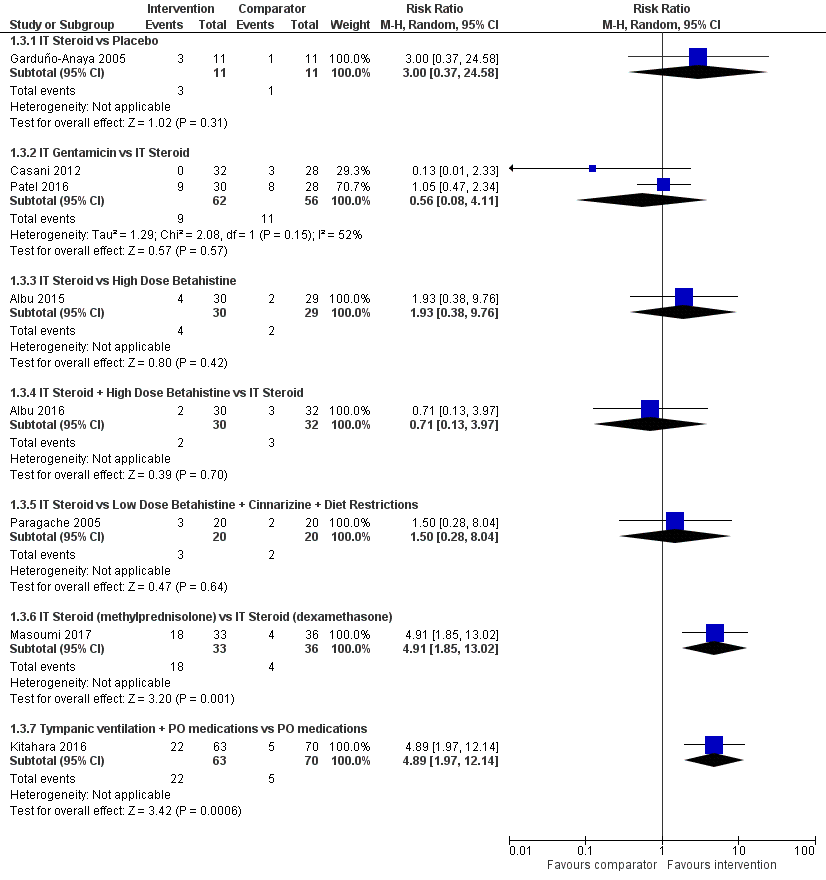


IT Steroid + High-dose Betahistine versus IT Steroid: There was no difference between IT steroid (dexamethasone) with and without high-dose betahistine for improved hearing >=10 dB (RR 0.71, 95% CI 0.13 to 3.97, N=62) based on one study (33).

IT Steroid versus Low-dose Betahistine + Cinnarizine + Diet restrictions: Based on one trial (30), there was no statistically significant difference between IT steroid (dexamethasone) and conventional therapy comprising of salt and caffeine restricted diet, nicotine and alcohol restrictions, cinnarizine for acute episodes and low-dose betahistine for improved hearing >= 10 dB (RR 1.50, 95% CI 0.28 to 8.04; N=40).

IT Steroid (methylprednisolone) versus IT steroid (dexamethasone): one study (29) compared the two steroids and IT methylprednisolone had significantly higher proportion of patients who had improved hearing >= 10 dB than IT dexamethasone (RR 4.91, 95% CI 1.85 to 13.02; N=69).

Tympanic ventilation + oral medication (including diuretics, betahistine, diphenidol, dimenhydrinate, and diazepam) versus the same oral medication alone: One study (32) demonstrated that tympanic ventilation combined with oral medication might be beneficial compared to the same oral medication alone (RR 4.89, 95% CI 1.97, 12.14 to N=133).

Oral Steroid (prednisolone) plus maintenance therapy (diphenidol + acetazolamide + low-sodium diet (< 1,500 mg/d) versus Maintenance therapy alone: hearing evaluation was carried out at both very low frequencies (125 and 250 Hz) and very high frequencies (4 to 8 kHz); however, investigators reported that the statistical analysis did not reveal significant differences between the groups in any frequency category (41).

ESD with and without steroid injection: In the group of patients treated with ESD plus steroid injection, all patients had stable hearing between baseline and 24 months after surgery except one with hearing improvement, while in the group of patients treated with ESD alone, two patients had improved hearing, two had reduced hearing and the remaining had stable hearing. Investigators combined data from both groups and reported hearing remained stable before and after surgery (28). The number of patients per group beyond baseline was unclear.

## 1.3 Speech Discrimination Score/ Speech Reception Threshold change

Eight studies reported changes in Speech Discrimination Score (SDS) (30,31,33,35,37,42,44,50) and one study reported changes in Speech Reception Threshold (SRT) (44). We could not analyze data from two trials, due to insufficient information regarding the number of patients beyond baseline (50), and unavailability of continuous SDS data (30).

Meta-analysis of SDS change was performed for one pairwise comparison, IT gentamicin vs. IT steroid (31,37,44). Three pairwise comparisons in SDS change were informed by one study each (33,35,42). Before analyses, we calculated the mean SDS changes and the corresponding SDs for five studies (31,33,35,37,44), assuming the correlation of 0.566 and 0.3 before and after intervention. The SDS change results are summarized in **Figure A6**.

**Figure A6**: Difference in Speech Discrimination Score (SDS) change, where positive SDS change per group indicates better speech discrimination. Five studies (31,33,35,37,44) reported the means and SDs before and after intervention but not the SDs of SDS changes. *: We calculated the mean SDS changes and the corresponding SDs, assuming the correlation of 0.566 before and after intervention. This correlation had been calculated from individual level data of 22 patients (42). **: Sensitivity analysis assuming the correlation of 0.3.


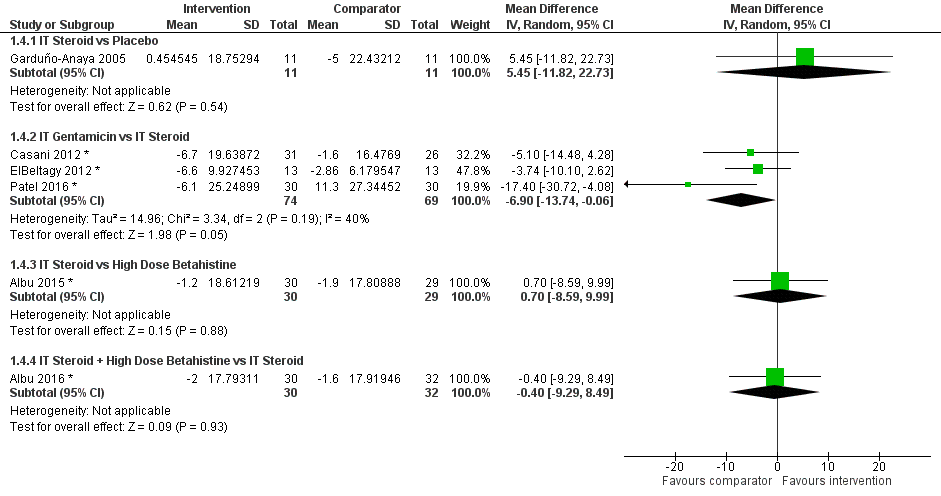

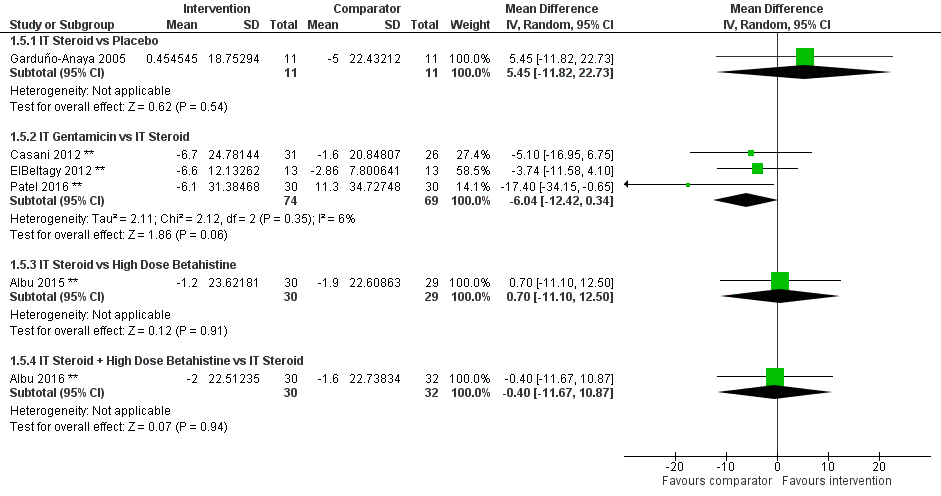


Findings from pairwise comparisons of interventions are as follows:

IT Steroid versus Placebo: Based on a single trial (42), deterioration of speech discrimination was, on average, worse in the placebo group than the IT steroid group; however, the difference was not statistically significant (mean difference 5.45, 95% CI -11.82 to 22.73; N=22).

IT Gentamicin versus IT Steroid: According to three trials (31,37,44), the level of deterioration in speech discrimination compared to pre-treatment, on average, was worse in the IT gentamicin group than the IT steroid group. The meta-analysis favored IT steroid and demonstrated fewer deterioration in speech discrimination for IT steroid compared to IT gentamicin (mean difference -6.90, 95% CI -13.74 to -0.06 if rho=0.566, or mean difference -6.04, 95% CI -12.42 to 0.34 if rho=0.3; N=143).

IT Gentamicin versus IT Steroid: Only one study reported SRT (44). The mean± SD of SRT reported at pre-intervention, 6 months and 1 year were 56.66±11.28, 59.13.25±13.25, and 57.8± 11.6 in IT gentamicin group, and 47± 14.7, 44.3± 8.83, and 40± 9.1 in IT steroid group. However, no significant difference was observed (p>0.05) between groups (44).

IT Steroid versus High-dose Betahistine: There was no significant difference between IT steroid and high-dose betahistine in terms of SDS change (mean difference 0.70, 95% CI -8.59 to 9.99 if rho=0.566, or -11.10 to 12.50 if rho=0.3; N=59) based on a single trial (35).

IT Steroid + High-dose Betahistine versus IT Steroid: There was no significant difference between IT steroid with and without high-dose betahistine (mean difference -0.40, 95% CI -9.29 to 8.49 if rho=0.566 or -11.67 to 10.87 if rho=0.3; N=62) based on a single trial (33).

EDB vs ESD: There was no significant difference between EDB and ESD in terms of SDS at all periods (last follow-up at 24 months) based on one trial (50). Six patients in the EDB group demonstrated a clinical improvement of SDS at 12 months post-surgery.

IT Steroid versus Low-dose Betahistine + Cinnarizine + diet restriction: The trial reported that three (15%) patients in IT steroid group and two (10%) patients in the conventional therapy group had SDS improvement while the rest remained the same (30).

## II. Vertigo

## 2.1 Vertigo control

**2.1.1 Pairwise meta-analysis (complete or substantial control vs other classes)**

**Figure A7:** Risk ratio of complete or substantial vertigo control versus other categories as per the 1995 AAO-HNS definition.

The AAO-HNS classes for vertigo control include complete (class A: numeric value 0) or substantial control (class B: 1-40), limited control (class C: 41-80), insignificant control (class D: 81-120), worse control (class E: >120), and secondary treatment initiated due to disability from vertigo (class F).

* In Stokroos and Kingma (2004) (43), the number of patients with no vertigo attacks or significant reduction in the frequency of attacks during follow-up (6-28 months) had been used.

** In Paragache et al (2005) (30), Sakata’s criteria (possibly excellent and good control of vertigo) had been used.

# In Morales-Luckie et al (2005) (41), maintenance therapy in both groups consisted of diphenidol (25 mg/d) plus acetazolamide (250 mg/48 h) and a low-sodium diet (< 1500 mg/d). Only patients with limited vertigo control (Class C) and severe disability (Scale 3) were included.


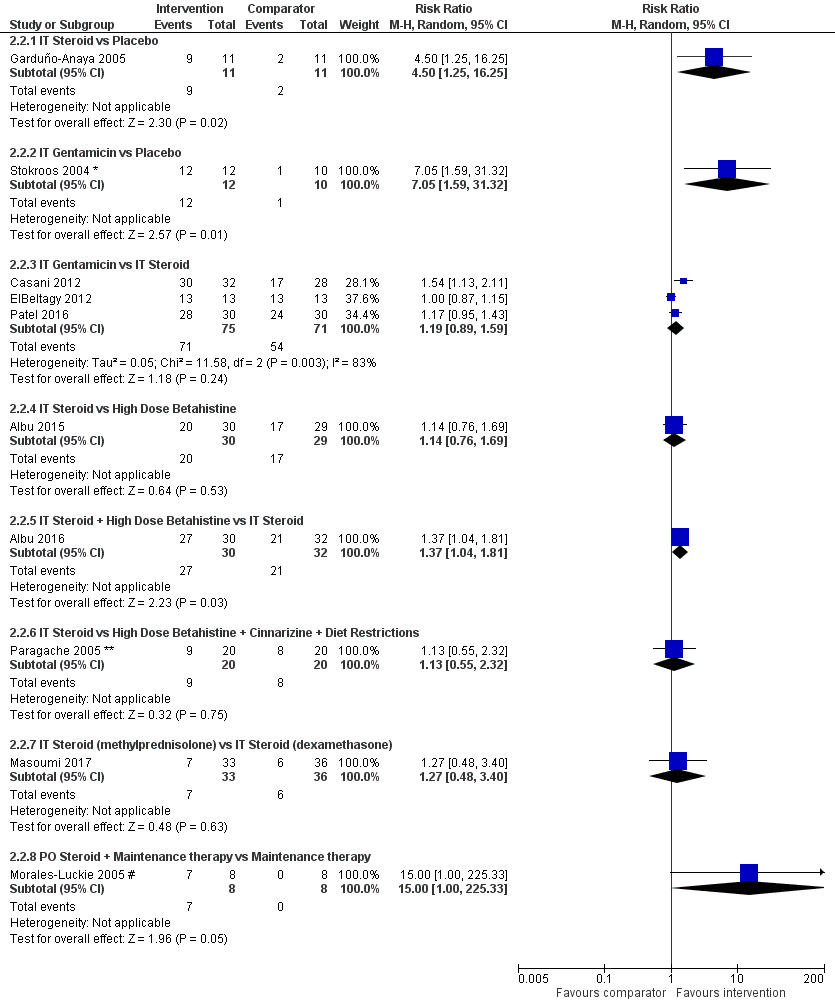


**2.1.2 Network meta-analysis of complete vertigo control**

We excluded Stokroos and Kingma (2004) (43) from the NMA of complete vertigo control because it reported that all 12 patients in the IT gentamicin arm versus zero patients in the placebo arm had no complaints of vertigo attacks six weeks after the last treatment and during follow-up (one patient in the placebo arm had a significant reduction in the frequency of vertigo attacks). This predominantly sharp contrast (12/12 vs 0/10) in complete vertigo control causes considerable uncertainty in the estimation of risk ratios (RR) and odds ratios (OR) for the comparison of IT gentamicin versus placebo, and the magnitude of RR and OR estimates was heavily dependent upon the model and continuity correction used. We did not run a sensitivity analysis for IT gentamicin with injections six weeks apart (Stokroos et al 2004) (43) as a treatment node separate from IT gentamicin in other studies because the resulting OR could be extremely large compared with any other interventions. Future large trials regarding the usage of IT gentamicin with injections six weeks apart would be informative toward quantifying its exact effect estimate and verifingy whether it is truly predominantly more beneficial than other interventions (including IT gentamicin 1-3 weeks apart) in terms of vertigo control.

Evaluations of model fit suggested that the RE unrelated means model had marginally lower values of deviance information criteria (DIC) than the RE consistency model (details provided in **Table A7, Appendix 4**), however the difference was fewer than five points (failing to meet our a priori criteria to suggest an important difference). We still preferred to report the results from the RE consistency model.

**Table A7:** Summary of model fit details for the network meta-analyses of complete vertigo control.

| **Model** | **No. of Arms / Studies** | **Total Residual Deviance** | **Deviance Information Criteria** | **Between-trial SD (95% CrI)** |
| --- | --- | --- | --- | --- |
| RE Consistency | 14 / 7 | 14.51 | 70.41 | 1.30 (0.05 to 4.19) |
| RE Unrelated Means |  | 13.29 | 69.18 | 1.22 (0.04 to 4.14) |

We present in **Figure A8** the results of pairwise comparisons in terms of complete vertigo control based upon NMA. IT gentamicin was associated with a probability of 0.989 to achieve better complete vertigo control than placebo. In descending order of mean SUCRA, IT gentamicin was followed by IT steroid plus high-dose betahistine, IT steroid, and high-dose betahistine (**Table A8**). The large odds ratio estimates of interventions compared to placebo should be interpreted with caution due to the presence of zero patients and one patient with complete vertigo control in the placebo group of the small trials (IT steroid vs. placebo (42) and IT gentamicin vs. placebo (39), respectively). The comparison-adjusted funnel plot (**Figure A9**) detected no evidence of small-study effects.

Considering that IT gentamicin was associated with strong performance to control vertigo but was found to be potentially detrimental for hearing preservation (often considered as chemical ablation) with high cumulative dosage and short interval between injections, a preferable approach to treatment may be IT steroid plus high-dose betahistine to achieve both hearing preservation (**Table A6**, **Figure A2**) and vertigo control (**Table A8**, **Figure A10**). However, IT steroid plus high-dose betahistine has only been compared in clinical trials with IT steroid alone (33), and future trials comparing it with other interventions head-to-head will be valuable to establish comparative effectiveness with additional direct evidence.

**Figure A8: League table of pairwise odds ratio estimates for complete vertigo control (lower triangle), and the probabilities that one treatment is better than another (upper triangle).** The league table presents pairwise odds ratio (OR) estimates for complete vertigo control along with credible intervals (2.5% and 97.5% quantiles), and the pairwise probabilities that a treatment is better than another based on NMA. A complete summary of estimates for efficacy from the RE consistency model assuming vague priors is displayed. Estimates of odds ratio between regimens which ruled out the possibility of no difference are shown in bold, underlined font. For each comparison, the lower/right-most treatment is the reference treatment. For example, the probability that IT gentamicin performs better than placebo in terms of complete vertigo control was 0.989. We were unable to convert OR estimates to risk ratios (as a function of OR and control group rate) due to zero patients with complete vertigo control in the placebo group of certain studies.

| **IT Gentamicin** | 0.535 | 0.926 | 0.864 | **0.989** |
| --- | --- | --- | --- | --- |
| 1.11 (0.02 to 84.95) | **IT Steroid + Betahistine (high dose)** | 0.838 | 0.818 | 0.960 |
| 3.87 (0.39 to 27.57) | 3.50 (0.07 to 112.30) | **IT Steroid** | 0.596 | 0.967 |
| 5.01 (0.08 to 347.40) | 4.56 (0.02 to 781.71) | 1.28 (0.04 to 64.60) | **Betahistine (high dose)** | 0.904 |
| **65.53 (2.55 to 2146.00)** | 61.45 (0.43 to 7304.05) | 17.25 (0.76 to 634.11) | 13.45 (0.10 to 1571.00) | **Placebo** |

**Figure A9:** Comparison-adjusted funnel plot for complete vertigo control

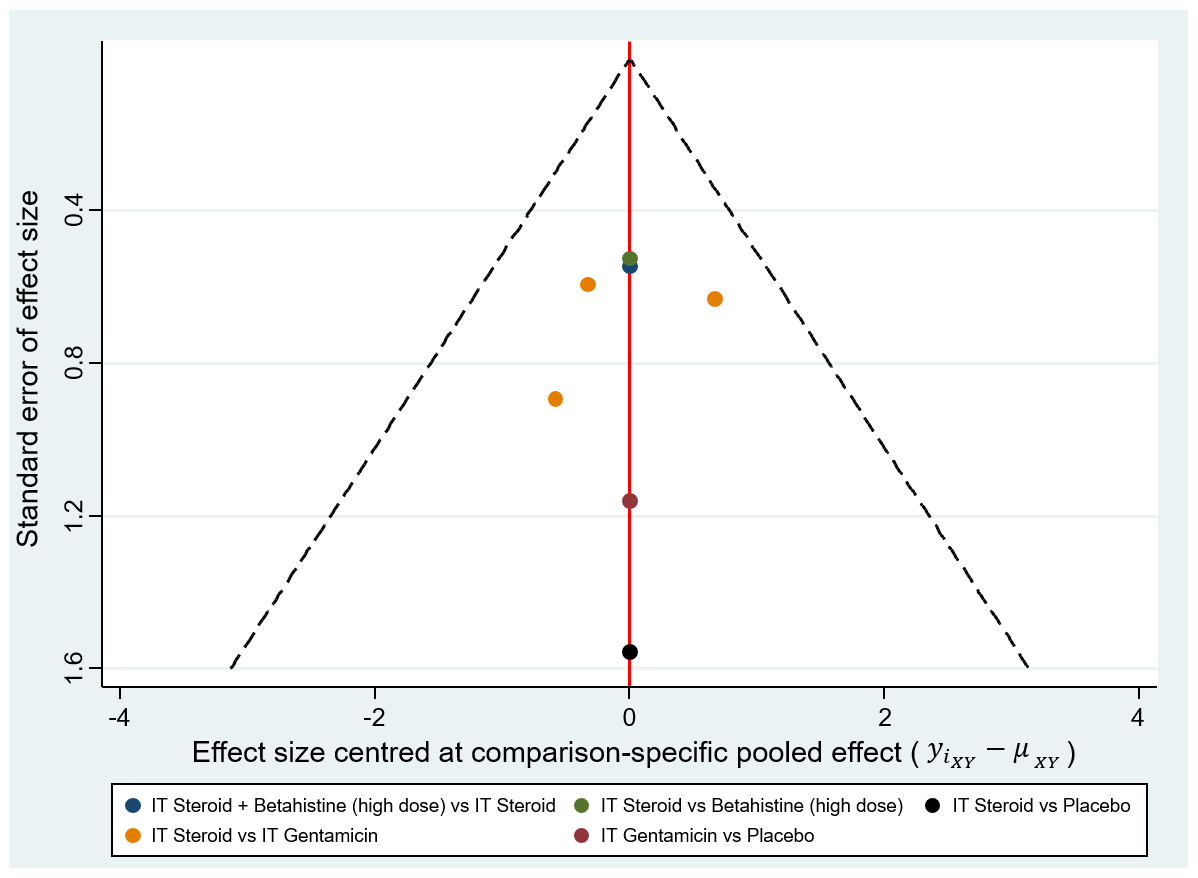


**Table A8:** Mean SUCRA value, mean probability to be the best treatment, and mean rank for each treatment based on complete vertigo control, with the treatments in descending order of mean SUCRA. These secondary measures of effect from NMA are displayed. Larger values of the mean SUCRA or the smaller values of the mean rank suggest better treatments. SUCRA: the Surface Under the Cumulative RAnking curve (SUCRA) value represents the surface underneath the cumulative ranking curve, which is the posterior probabilities for each drug to be among the n-best options.

| **Outcome: hearing change** | **Mean SUCRA** | **Mean Pr(best)** | **Mean Rank*** |
| --- | --- | --- | --- |
| **IT Gentamicin** | 0.828 | 0.473 | 1.69 (1 to 4) |
| **IT Steroid + Betahistine (high dose)** | 0.770 | 0.431 | 1.92 (1 to 5) |
| **IT Steroid** | 0.450 | 0.014 | 3.20 (2 to 4) |
| **Betahistine (high dose)** | 0.407 | 0.079 | 3.37 (1 to 5) |
| **Placebo** | 0.045 | 0.003 | 4.82 (3 to 5) |

* Mean rank with 2.5% and 97.5% quantiles in parentheses.

**Figure A10:** The probability of each treatment to be to be at a specific rank for hearing change from the RE consistency model


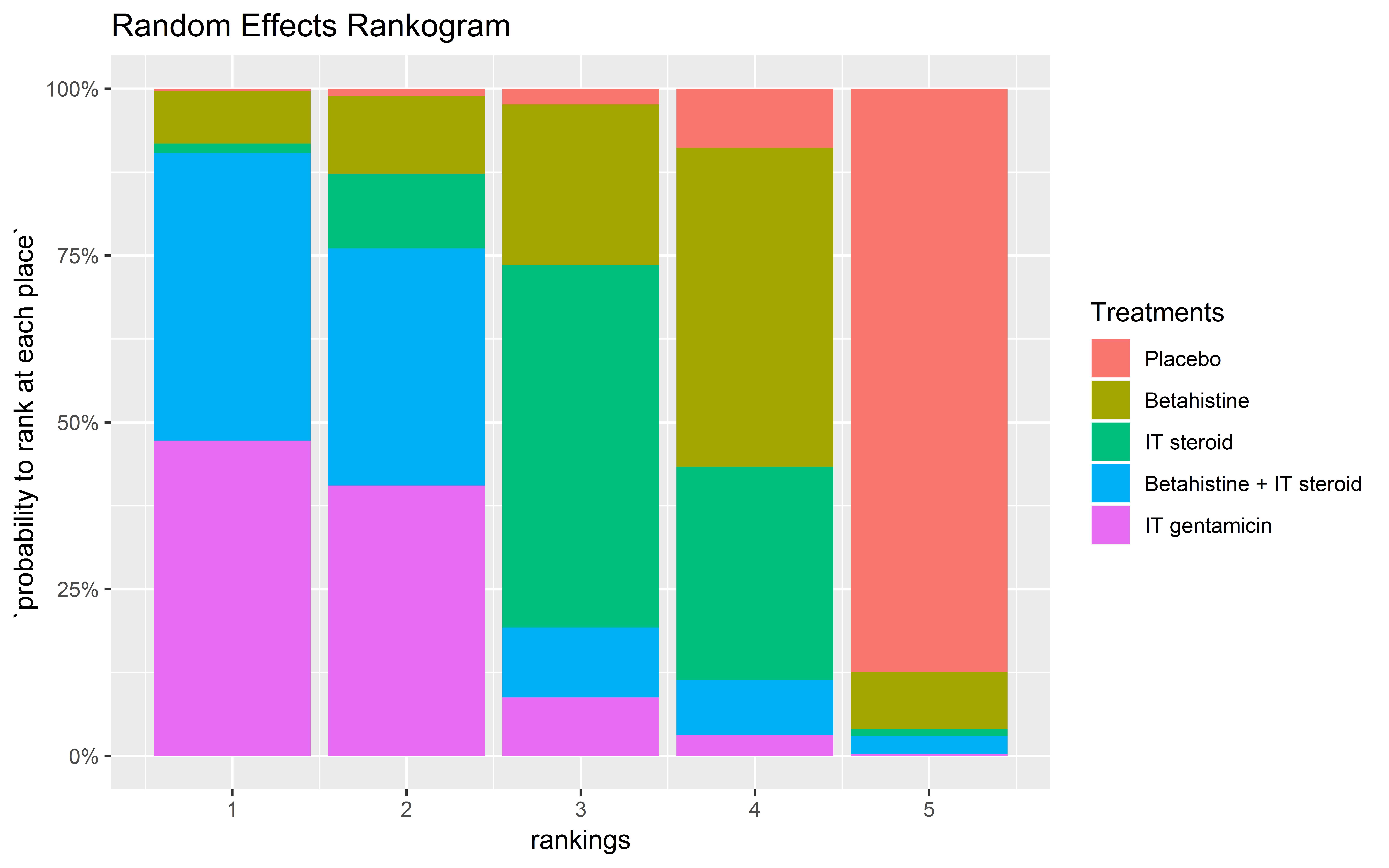


## 2.2 Vertigo frequency

Ten studies reported vertigo frequency (28,31,34,35,38,41–44,50). We could not perform quantitative analysis for vertigo frequency except for one study in which individual data revealed that vertigo frequency was highly skewed and zero-inflated (42). In addition, our decision not to run meta-analyses for vertigo frequency was associated with other reasons. These included insufficient information regarding the number of patients beyond baseline (28,50), absence of standard deviation (34,35), and variation in the unit of analysis (vertigo attack per day (41), month (31), 6 months (44), 12 months (43)). A narrative summary is presented next.

IT Gentamicin versus IT Steroid: There was a 90% reduction in the mean number of vertigo attacks in the methylprednisolone group (mean ± SD, 6 months before the first injection and before 24 months: from 16.4 ± 12.5 to 1.6 ± 3.4), and 87% reduction in the gentamicin group (from 19.9 ± 16.7 to 2.5 ± 5.8). However, no significant difference was noted between groups in the final six months (mean difference -0.9, 95 % CI -3.4 to 1.6). Similarly, the mean number of vertigo attacks at 24 months did not differ between groups (mean difference -0.2, 95% CI -1.4 to 0.9) and both drugs reduced the number of attacks (methylprednisolone by 91% and gentamicin by 90%) (31).

The mean number of vertigo attacks decreased from 43.8 ± 63.8 at baseline to 14.2 ±32.6 at 6-month follow-up in the gentamicin group and from 43.8 ± 63.8 to 2.47 ± 2.33 in the dexamethasone group. Similarly, at 1-year follow-up, both groups demonstrated a statistically significant reduction in the mean number of attacks (mean± SD was 11.8±29 in gentamicin group, and 2.9±2.5 in the dexamethasone group at 1-year). However, the differences of changes between groups were non-significant at both 6-month and 1-year follow-up (p>0.05) (44).

EDB versus ESD: There was a statistically significant difference in the mean ± SD number of vertigo spells at 24 months post-surgery (0.3±0.7 in the EDB group and 4.5±7.9 in the ESD group, p=0.0002); however the difference was not statistically significant in the last six months before the surgery (8.4±5.8 in the EDB group and 9.6±7.9 in the ESD group, p=0.1532). The number of patients per group beyond baseline was unclear (50).

Oral Steroid (prednisolone) plus maintenance therapy (diphenidol + acetazolamide + low-sodium diet (< 1,500 mg/d) versus Maintenance therapy alone: The mean number of vertigo attack per day was similar between the groups at the baseline (1.27 in prednisone plus maintenance therapy group and 1.01 in maintenance therapy group, p-value not significant between groups) among patients with limited vertigo control (Class C) and severe disability (Scale 3); however, the number of vertigo attacks reduced substantially to 0.50 (95% CI 0.34 to 0.66) in the prednisone group, while maintained 1.00 (95% CI 0.79 to 1.20) in the control group (p<0.01 for comparison between groups), then stayed constant till the end of study (0.46, 95% CI 0.23 to 0.69 in prednisone group and 0.90, 95% CI 0.68 to 1.12 in the control group, p<0.01 between groups) (41).

High and Low-dose Betahistine (144 mg/day and 48 mg/day) vs Placebo: There was no difference in vertigo attack rates between the three treatment groups (p=0.850 for intention-to-treat analysis and p=0.808 for per protocol analysis, using full analysis set after multiple imputation). the mean attack rate per 30 days was 2.36 (95% CI 1.58 to 3.71) in the placebo group, 2.00 (95% CI 1.32 to 3.02) in the low-dose betahistine group, and 2.09 (95% CI 1.37 to 3.20) in the high-dose betahistine group (34).

IT Steroid versus High-dose Betahistine: There was a significant reduction in mean number of vertigo attacks per month from baseline and during follow up in both the IT dexamethasone (8.6 at baseline, 1.8 at one month, and 1.5 at three months) and the high-dose betahistine (7.9 at baseline, 4.3 at one month, and 2.4 at three months) treatment groups. It was noted that IT dexamethasone had reduced vertigo attacks more rapidly than betahistine; however, the numbers of vertigo attacks maintained at a low level and were similar between the two treatments at 6, 9 and 12 months (35).

Low-dose Betahistine (24 mg twice daily versus 16 mg three times daily): The proportions of patients having two or more spells of active vertigo attack lasting more than one hour were 76.6% and 73.4% at baseline in betahistine 24 mg twice daily and 16 mg three times daily treatment groups, respectively. In contrast, no vertigo or one spell of acute vertigo lasting less than one hour was observed by 86.7% and 88.3% of patients at 12 weeks, and by 95.0% and 96.7% of patients at 24 weeks in the 16 mg and 24 mg treatment groups, respectively (38).

IT Gentamicin vs Placebo: There was more reduction in the number of vertiginous attacks per year in gentamicin group compared to placebo group, despite the baseline imbalances across groups (mean± SD in gentamicin group was 74±114 prior to treatment and 0 after treatment, p=0.002, and in placebo group was 25± 31 prior to treatment and 11± 10 after treatment, p=0.028) (43).

ESD plus steroid injection versus ESD alone: No statistically significant difference was observed between the steroid and non-steroid treatment groups (ESD plus steroid injection versus ESD alone) in the mean number of vertigo episodes per month (3.3 at baseline, 1.4 at 6 months, 3.9 at 12 months and 0.26 at 24 months in the steroid group, and 3.4 at baseline, 0.5 at 6 months, 2.0 at 12 months and 1.1 at 24 months in the non-steroid group). As such, the investigators combined data from both groups to assess the overall effectiveness of endolymphatic sac surgery on vertigo control. They reported that patients had fewer complaints of vertigo at 6, 12, and 24 months compared to before ESD surgery (p<0.05) (28). The number of patients per group beyond baseline was unclear.

IT Steroid versus Placebo: The mean definitive vertigo episodes per month reduced in both the IT steroid group (mean change -0.67, SD 0.69) and the placebo group (mean change -0.13, SD 1.13) between baseline and six months (42). If vertigo frequency can be considered as a normally distributed continuous outcome, IT steroid might result in more reduction in vertigo frequency than placebo (-0.55 episodes per month, 95% CI -1.33 to 0.24, N=22) but the difference was not significant. Nevertheless, we noted that data on vertigo frequency in this study was highly skewed and zero-inflated according to individual patient data (42).

## 2.3 Vertigo severity

Two studies reported vertigo severity (31,39).

IT Gentamicin versus IT Steroid: There was a 76% reduction in the mean number of vertigo symptom scale score (0-60) in the methylprednisolone group (from 21.8±10.5 at baseline to 5.3±6.3 at 24 months, and 67% reduction in the gentamicin group (from 24.7 ± 12.6 to 8.2 ± 11.4). However, no significant difference was noted between groups at 24 months (mean difference -2.9, 95 %CI -7.6 to 1.7) (31).

IT Gentamicin vs Placebo**:** The mean number of vertigo score [a four point scale: severe (3), moderate (2), mild (1) and none (0)] significantly decreased in gentamicin group (from 2.1±0.8 at baseline to 0.5±0.6 at 1 year) but not in placebo group (from 2.0±0.8 at baseline to 1.8±1.0 at 1 year) (39).

## III. Tinnitus

Eleven studies reported tinnitus (28,30,31,33–35,39,41,42,44,50). Of these, five reported the tinnitus handicap inventory (THI) (28,31,33,35,42), one reported tinnitus severity (39), intensity (34) and persistence (50), one reported whether tinnitus occurrence was continuous or intermittent (41), and two reported changes in tinnitus (completely relieved or improved (30) and disappeared, improved, remained the same or worsened (44)). Meta-analysis was precluded due to insufficient number of studies per treatment comparisons. The findings are presented next.

## 3.1 THI

Five studies reported this outcome (28,31,33,35,42). Meta-analysis was not possible because each pairwise comparison had a single study informing FLS before and after intervention (31,33,35,42). We denote the correlation between pre- and post-treatment as rho. **Figure A11** provides details on four pairwise comparisons. Another trial which compared endolymphatic sac decompression (ESD) with and without steroid injection had insufficient information regarding the number of patients beyond baseline (28), so we provided a narrative summary.

The THI score was based on 25 items divided into catastrophic, emotional, and functional subsets. Each question was answered with “no”, worth 0 points, “sometimes”, worth 2 points, or “yes”, worth 4 points. The higher values of the THI score reflect greater perceived disability from tinnitus.

**Figure A11**: Difference in Tinnitus Handicap Inventory (THI) change, where negative THI change per group indicates reduction in tinnitus handicap. For studies which reported mean and SD of THI before and after intervention, we assumed the correlation pre- and post-intervention to be 0.4 and 0.6 and calculated the mean THI changes and the corresponding SDs.


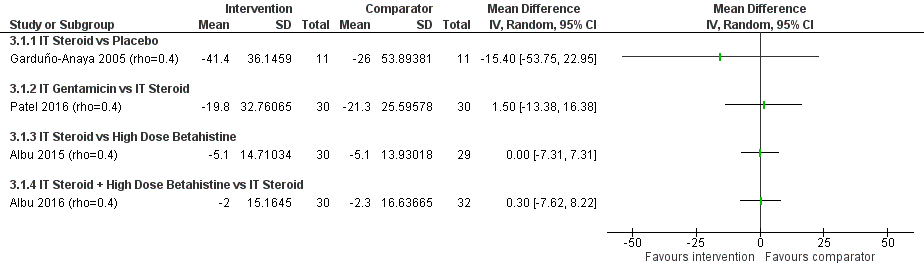


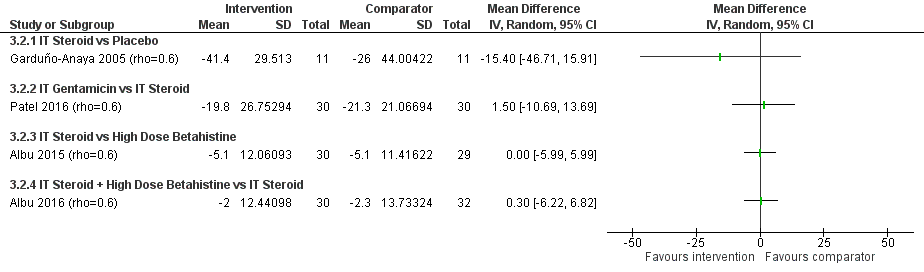


IT Steroid versus Placebo: Patients’ tinnitus on average reduced more in the IT steroid group than in the placebo group; however, the between group difference was not statistically significant (mean difference -15.40, 95% CI -53.75 to 22.95 if rho=0.4 or -46.71 to 15.91 if rho=0.6; N=22) based on a single trial (42).

IT Gentamicin versus IT Steroid: On average, patients performed similarly with regards to reducing tinnitus in both IT gentamicin and IT steroid groups and there was no difference between them (mean difference 1.50, 95% CI -13.38 to 16.38 if rho=0.4 or -10.69 to 13.69 if rho=0.6; N=60) based on a single trial (31). The investigators also reported the mean difference of THI at 24 months as -7.8 (95% CI -21.3 to 5.6).

IT Steroid versus High-dose Betahistine: On average, patients performed similarly with regards to reducing dizziness in both IT steroid and high-dose betahistine groups and there was no difference between the groups (mean difference 0.00, 95% CI -7.31 to 7.31 if rho=0.4 or -5.99 to 5.99 if rho=0.6; N=59) based on a single trial (35).

IT Steroid + High-dose Betahistine versus IT Steroid: Both IT steroid with and without high-dose betahistine performed similarly with regards to reducing dizziness and there was no difference between groups (mean difference 0.30, 95% CI -7.62 to 8.22 if rho=0.4 or -6.22 to 6.82 if rho=0.6; N=62) based on a single trial (33).

ESD plus steroid injection versus ESD alone: No statistically significant difference was observed between the steroid and non-steroid treatment groups in terms of THI (47 at baseline, 33.5 at 6 months, 32.3 at 12 months and 29.1 at 24 months in the steroid group, and 41.4 at baseline, 24 at 6 months, 22.5 at 12 months and 13.8 at 24 months in the non-steroid group). However, patients had lower THI at 6, 12, and 24 months (p<0.05) compared with THI before ESD surgery (28). The number of patients per group beyond baseline was unclear.

## 3.2 Tinnitus intensity

High and Low-dose Betahistine (144 mg/day and 48 mg/day) vs Placebo: There was no difference in tinnitus intensity (dB) between the three treatment groups. Compared to the placebo group, the difference in the adjusted mean change of tinnitus intensity was 1.40 (95% CI -5.10 to 7.90) in the low-dose betahistine group and -3.34 (95% CI -9.74 to 3.06) in the high-dose betahistine group (34).

## 3.3 Tinnitus persistence

EDB vs ESD: There was a significant difference in the proportion of patients with tinnitus persistence at 12 months (40% in the EDB group versus 87.5% in the ESD group, p=0.0366), 18 months (33.3% in the EDB group versus 80.1% in the ESD group, p=0.0201) and 24 months (31% in the EDB group versus 80.1% in the ESD group, p=0.021) except for at 6 months (80% in the EDB group versus 84.62% in the ESD group, p=0.727) (50). The number of patients per group beyond baseline was unclear.

## 3.4 Tinnitus severity

Tinnitus severity was scored on a four-point scale: severe (3), moderate (2), mild (1) and none (0).

IT Gentamicin vs Placebo: IT gentamicin and placebo did not change tinnitus severity score at 12 months compared to the baseline. The tinnitus severity score (mean± SD) was 2.5 ±0.8 at baseline and 2.3±0.8 at 12 months in gentamicin group, and 2.4±0.8 at baseline and 2.2±0.7 at 12 months in the placebo group (39). Tinnitus complaints remained moderate to severe in both groups.

## 3.5 Changes in tinnitus symptom

The total number of tinnitus attacks per month were classified as absent (tinnitus disappeared), intermittent (symptoms appeared and disappeared intermittently), or continuous (symptoms were always present throughout the day) (41), a subjective report of disappearance, improvement, same and worse (44), and unclear classification (30).

Oral Steroid (prednisolone) plus maintenance therapy (diphenidol + acetazolamide + low-sodium diet (< 1,500 mg/d) versus Maintenance therapy alone: Only patients with limited vertigo control (Class C) and severe disability (Scale 3) were included. Patients in both groups had continuous tinnitus; however, 50% (N=4) of the eight patients treated with prednisolone plus maintenance therapy demonstrated intermittent tinnitus at 18 weeks while the remaining 50% had no change in tinnitus pattern. None of the patients in the maintenance therapy group displayed any symptomatic modification (41).

IT Gentamicin versus IT Steroid: There was a non-significant difference between the two groups (p=0.099) on tinnitus. At six months, 13.3% (N=2) of patients reported disappearance of tinnitus, 13.3% (N=2) had improvement, 40% (N=6) observed the same intensity, and 33.3% (N=5) observed worsening of their symptoms in the IT gentamicin group. In IT dexamethasone group at six months, 13.3% (N=2) of patients reported disappearance, 26.7% (N=4) had improvement, 60% (N=9) observed no change and there was no case of worsening. At 12 months, 15.4% (2/13) reported disappearance, 15.4% observed improvement, 38.5% (5/13) had the same intensity, and 30.7% (4/13) reported worsening of their symptoms in the IT gentamicin group. However, in the IT dexamethasone group at 12 months, 7.7% (1/13) reported disappearance, 30.77% (4/13) were improved, 53.85% (7/13) had the same intensity and 7.7% (1/13) experienced worsening (44).

IT Steroid versus Low-dose Betahistine + Cinnarizine + diet restriction: Complete relief of tinnitus was reported in 10% (N=2) patients, and improved tinnitus in 605 (N=12) patients in the IT dexamethasone group while 15% (N=3) patients demonstrated complete relief, and 50% (N=10) showed some improvement in the conventional medical treatment group (30).

## IV. Relief from aural fullness

Seven studies reported aural fullness (30,31,39,41,42,44,50). Meta-analysis was not possible because single studies reported either binary or ordinal or continuous data on various aspects of the aural fullness; for instance, number of patients with complete relief from aural fullness or some improvement (30), analogue scale (0-10) score (31), categorical scale (0-3) severity score (39), proportion of persistent aural fullness (50), number of patients with 100% or 90% subjective improvement (42), frequencies of various categories of aural fullness change (disappeared, improved, remained the same, or worsened) (44), and a narrative finding (41).

Of these, the findings from three studies (30,42,44) are depicted in **Figure A12** demonstrating no significant difference for the pairwise comparisons (IT steroid versus placebo (42) at 24 months, IT gentamicin vs IT steroid (44) at 12 months, and IT steroid versus low-dose betahistine combined with cinnarizine and diet restrictions (30) at 6 months) on complete relief from aural fullness and/or improvement.

**Figure A12**: Risk ratio of relief from aural fullness.


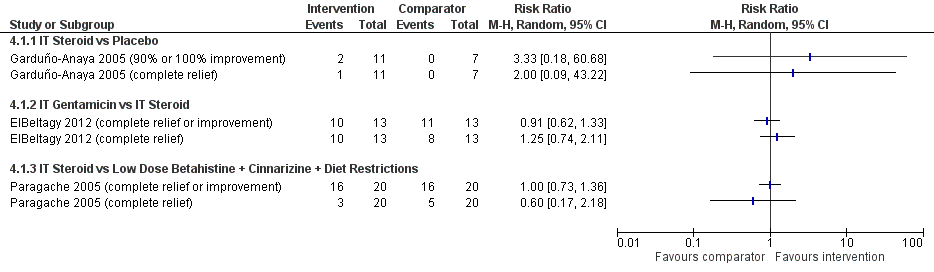


One study (41) scored aural fullness from 0 to 5, where 0 indicated no aural fullness and 5 indicated the worst possible status, treated patients for 18 weeks with a 12 months surveillance after prednisolone withdrawal. It only narratively stated that prednisolone combined with maintenance therapy compared to maintenance therapy alone did not produce statistically significant differences in aural fullness (41). Another trial which compared EDB versus ESD (50) reported statistically significant differences between the proportion of patients having aural fullness persistence at 12 months (41.18% in EDB versus 87.5% in ESD group, p=0.030), 18 months (25.3% in EDB versus 66.7% in ESD group, p=0.023) and 24 months (25% in EDB versus 81% in ESD group, p=0.014) in favor of EDB, however, the number of patients at each follow-up time point was unknown. One trial (31) which measured aural fullness in the analogue scale ranged 0-10 did not show a statistically significant difference at 24 months between the IT gentamicin and IT steroid (mean difference -0.6, 95% CI -2.0 to 0.8). Another trial (39) which measured aural fullness with a severity score ranged 0-3 (3: severe, 2: moderate, 1: mild, and 0: none) reported that mean change in the aural fullness severity score was significantly lower in the gentamicin group than the placebo group after one year (mean±SD: 1.8±1.1 in placebo group, and 0.9±1.1 in gentamicin group). We could not plot the data of these different pairwise comparisons in the same figure due to various metrics of measuring aural fullness.

## V. Handicap, disability and impairment of Quality of Life (QoL) measures

Nine studies reported quality of life using various measures, including Meniere’s Disease Outcomes Questionnaire (MDOQ) (28), Self-rating Depression Scale and Stress Response Scale-18 (SRS-18) (32), Functional Level Score (FLS) (31,33,35,37,42), mini-tinnitus impairment questionnaire score based on 12 items (MiniTF-12) and the Vestibular Disorders Activities of Daily Living (VDADL) (34), and disability as per AAO-HNS criteria (41).

## 5.1 FLS

Five studies reported FLS (31,33,35,37,42), a six-point functional level scale based on AAO-HNS 1995. Meta-analysis was not possible because each pairwise comparison had a single study informing changes in FLS (42) or FLS before and after intervention (31,33,35). We denote the correlation between pre- and post-treatment as rho. **Figure A13** provides details of FLS analysis for four pairwise comparisons.

**Figure A13**: Difference in Functional Level Scale (FLS) change, where negative FLS change per group indicates improvement in function and quality of life. For studies which reported mean and SD of FLS before and after intervention, we assumed the correlation pre- and post-intervention to be 0.4 and 0.6 and calculated the mean FLS changes and the corresponding SDs.


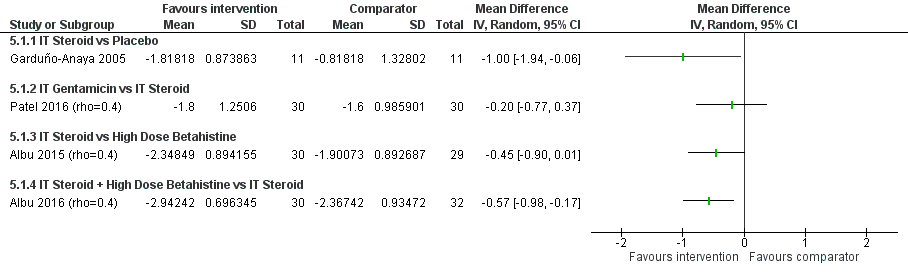

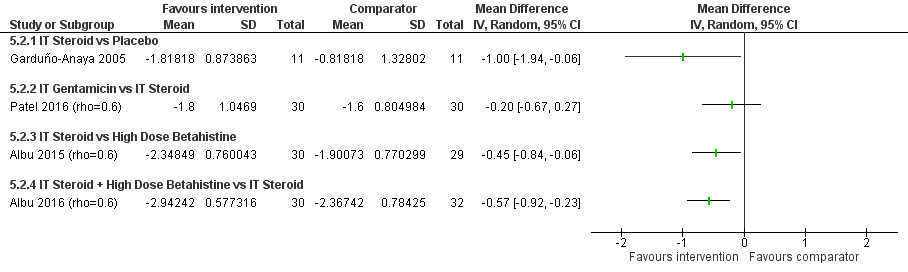


IT Steroid versus Placebo: IT steroid was associated with more decrease in FLS (more functional improvement) than placebo (mean difference -1.00, 95% CI -1.94 to -0.06; N=22) based on a single trial (42).

IT Gentamicin versus IT Steroid: On average, patients performed similarly on the improvement of functional level in both IT gentamicin and IT steroid groups. The difference between IT steroid and placebo on patients’ FLS change was not significant (mean difference -0.20, 95% CI -0.67 to 0.27 if rho=0.6 and -0.77 to 0.37 if rho=0.4; N=60) at 24 months based on a single trial (31) . The investigators also reported the mean difference of FLS at 24 months as -0.3 (95% CI -0.9 to 0.2).

IT Steroid versus High-dose Betahistine: Based on a single trial (35), patients on average experienced more decrease in FLS (more functional improvement) in the IT steroid group than the high-dose betahistine group (mean difference -0.45, 95% CI -0.84 to -0.06 if rho=0.6 or -0.90 to 0.01 if rho=0.4; N=59).

IT Steroid + High-dose Betahistine versus IT Steroid: A single trial (33) demonstrated that IT steroid plus high-dose betahistine group was associated with more decrease in FLS (more functional improvement) than the IT steroid only group (mean difference -0.57, 95% CI -0.92 to -0.23 if rho=0.6 or -0.98 to -0.17 if rho=0.4; N=62).

In addition, FLS improvement to level one was observed in 13 (40.6%) of patients in the IT gentamicin group and 11 (39.3%) patients in the IT dexamethasone group with no between group difference (p>0.05) at one year (37). However, a significant difference was reported at two years (p<0.05) as 22 (71%) patients achieved level one and 8 (25.8%) improved to level two in IT gentamicin group while only 12 (46.1%) reached level one and 5 (19.2%) got to level two in IT dexamethasone group (37).

## 5.2 VDADL

Only one study reported this outcome (34).

High and Low-dose Betahistine (144 mg/day and 48 mg/day) vs Placebo: The total Vestibular Disorders Activities of Daily Living (VDADL) score is defined as the median value of answers across all 28 questions. The higher values reflect greater perceived disability and impairment of QoL. The mean VDADL change from baseline to 9 months was -0.20 (95% CI -0.41 to -0.00, N=57) in the placebo group, -0.26 (95% CI -0.46 to -0.06, N=58) in the low-dose betahistine group, and -0.36 (95% CI -0.56 to -0.16, N=58) in the high-dose betahistine group (34). However, the difference of adjusted mean changes in VDADL did not show any difference between both betahistine doses and placebo (it was -0.05, 95% CI -0.32 to 0.22 between low-dose betahistine and placebo, and -0.06, 95% CI -0.33 to 0.20 between high-dose betahistine and placebo).

**Figure A14**: Difference in mean VDADL change, where negative VDADL change per group indicates improvement in function and quality of life.

**
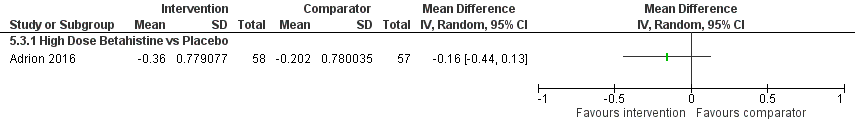
**

## 5.3 DHI

The Dizziness Handicap Inventory score was based on 25 items divided into physical, emotional, and functional subsets. Each question is answered with “no” (worth 0 points), “sometimes” (worth 2 points), or “yes” (worth 4 points). The higher values reflect greater perceived dizziness handicap disability and impairment of QoL. Findings from pairwise comparisons of interventions are as follows:

IT Steroid versus Placebo: Patients’ dizziness on average improved more in the IT steroid group than in the placebo group; however, the between group difference was not significant (mean difference -24.90, 95% CI -51.86 to 2.06 if rho=0.6 or -57.90 to 8.10 if rho=0.4; N=22) based on a single trial (42).

High-dose Betahistine versus Placebo**:** In one article (34), the 25-item DHI score was derived by averaging over all available answers to DHI components to deal with missing items; therefore, we multiplied the reported DHI change by 25. There was no difference in DHI improvement between high-dose betahistine and placebo groups (mean difference in unadjusted changes -0.45, 95% CI -7.20 to 6.30; N=113) based on a single trial (34).

IT Gentamicin versus IT Steroid: On average, patients performed similarly with regards to improving dizziness in both IT gentamicin and IT steroid groups and there was no difference between them (mean difference -0.10, 95% CI -10.00 to 9.80 if rho=0.6 or -12.09 to 11.89 if rho=0.4; N=60) based on a single trial (31). The investigators also reported the mean difference of DHI at 24 months as -8.2 (95% CI -19.7 to 3.3).

IT Gentamicin versus IT Steroid: There was a nonsignificant difference between the two groups (p=0.215) in DHI at 12 months. At six months, 46.7% (N=7) of patients had mild handicap, 46.7% (N=7) had moderate handicap, 6.7% (N=1) had moderately severe handicap, in the IT gentamicin group. In IT dexamethasone group at six months, 26.7% (N=4) of patients had mild handicap, 60% (N=9) had moderate handicap, and 13.3% (N=2) had moderately severe handicap. At 12 months, 46.1% (N=6) of patients had mild handicap, 53.9% (N=7) had moderate handicap, and no patients had moderately severe handicap in the IT gentamicin group. In IT dexamethasone group at 12 months, 23.1% (N=3) of patients reported had mild handicap, 61.5% (N=10) had moderate handicap, and 15.4% (N=2) had moderately severe handicap. (44).

IT Steroid versus High-dose Betahistine: On average, patients performed similarly with regards to improving dizziness in both IT steroid and high-dose betahistine groups and there was no difference between them (mean difference -2.10, 95% CI-9.34 to 5.14 or -10.82 to 6.62 if rho=0.4; N=59) based on a single trial (35).

ESD plus steroid injection versus ESD alone: The study reported no statistically significant difference between the steroid and non-steroid treatment groups (p>0.05) in terms of DHI (49.2 preoperatively, 30.7 at 6 months, 35.5 at 12 months and 33.7 at 24 months in the steroid group, and 60.5 preoperatively, 23.5 at 6 months, 25.8 at 12 months and 29.1 at 24 months in the non-steroid group). The number of patients per group beyond baseline was unclear (28).

**Figure A15**: Difference in Dizziness Handicap Inventory (DHI) change, where negative DHI change per group indicates reduction in dizziness handicap. For studies which reported mean and SD of DHI before and after intervention, we assumed the correlation pre- and post-intervention to be 0.4 and 0.6 and calculated the mean DHI changes and the corresponding SDs.


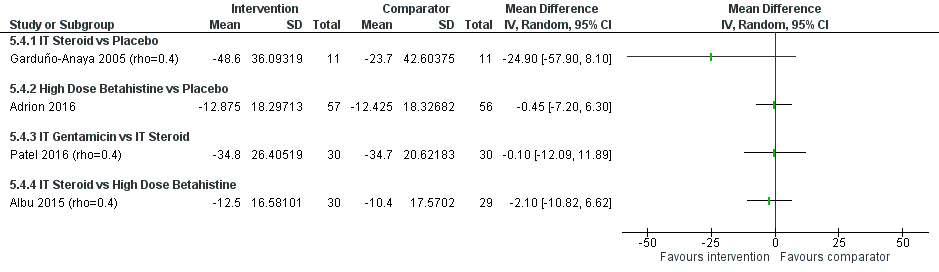


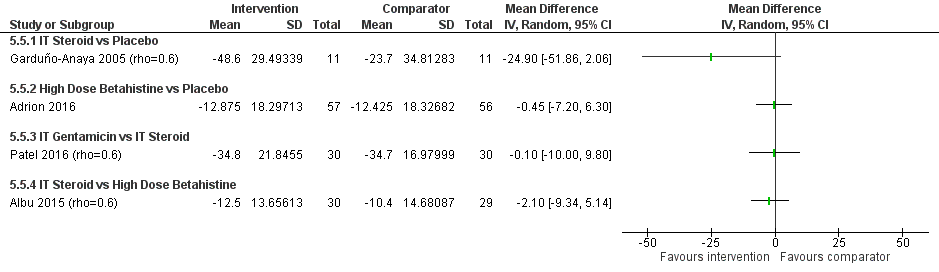


## 5.4 Self-assessed functional disability

Oral Steroid (prednisolone) plus maintenance therapy (diphenidol + acetazolamide + low-sodium diet (< 1,500 mg/d) versus Maintenance therapy alone: The self-assessed functional disability was based on a scale of 0 (none), 1 (mild), 2(moderate) and 3 (severe). Only patients with limited vertigo control (Class C) and severe disability (Scale 3) were included. One year after withdrawal of treatment, seven of eight patients in prednisolone plus maintenance therapy group reported mild disability, and one patient in this group along with eight patients in the maintenance therapy group remained with severe functional disability (41).

## 5.5 MiniTF

Only one study reported this outcome (34).

High and Low-dose Betahistine (144 mg/day and 48 mg/day) vs Placebo: MiniTF is a self-administered questionnaire on tinnitus impairment based on 12 items. The higher values reflect greater perceived disability and impairment of QoL. To deal with missing items for the MiniTF questionnaire, the mean total scores was derived by averaging the number of available answers in the study reported this outcome (34). The unadjusted mean MiniTF change from baseline to 9 months reported -0.12 (95% CI -0.22 to -0.02, N=54) in the placebo group, -0.11 (95% CI -0.21 to -0.01, N=58) in the low-dose betahistine group, and -0.14 (95% CI -0.24 to -0.04, N=56) in the high-dose betahistine group (34). However, both betahistine doses were not different from placebo in terms of MiniTF change. The difference of adjusted mean change was -0.01 (95% CI -0.14 to 0.13) between low-dose betahistine and placebo, and -0.02 (95% CI -0.15 to 0.11) between high-dose betahistine and placebo.

## 5.6 MDOQ

Only one study reported this outcome (28).

ESD plus steroid injection versus ESD alone**:** The average MDOQ, measuring quality of life, did not differ in patients underwent surgery with and without steroid at 2, 6, 12 and 24 months postoperative based on data from one trial (28). The number of patients per group beyond baseline was unclear.

## VI. Self-rating Depression Scale and Stress Response Scale-18 (SRS-18)

Only one study reported this outcome (32).

Tympanic ventilation tube + Medication (including diuretics, betahistine, diphenidol, dimenhydrinate, and diazepam) versus medication alone: Self-rating Depression Scale consists of 10 positively and 10 negatively worded items that enquire about symptoms of depression. SDS scores >40 (possible range 20–80) were classified as having depression. Stress Response Scale-18 (SRS-18) consists of 18 items that enquire about stressful feelings due to stressful lifestyle. Patients with SRS-18 scores >20 (possible range 0–54) were classified as having high stress.

There was no difference in both tympanic ventilation tube + medication and medication alone groups from baseline to 24 months with regards to Self-rating Depression Scale (mean± SD was 35.1±9.6 at baseline and 34.3±8.7 at 24 months in tympanic ventilation tube + medication group and 32.1±9.1 at baseline and 33.6±9.1 at 24 months in medication alone group) and SRS-18 (mean± SD was 19.7 ±6.4 at baseline and 19.5±6.2 at 24 months in tympanic ventilation tube group and 19.7±6.3 at baseline and 20.2±5.8 at 24 months in medication group) (32). We did not calculate the difference of their changes between groups because the number of patients per group beyond baseline was unclear.
