# Supplementary material for: Pharmacologic and surgical therapies for patients with Meniere’s disease: A systematic review and network meta-analysis
Source: PLoS One. 2020 Sep 1;15(9):e0237523. doi: 10.1371/journal.pone.0237523 (PMC7462264; doi:10.1371/journal.pone.0237523)
Supplement: S5 Text — (DOCX) [file pone.0237523.s005.docx]

# S5 Text: Differences between the current review and two existing SRs with NMA

**Table A9**- The differences between the current review and two existing SRs with NMA (Cao et al 2019, and Hao et al 2019), in terms of inclusion and data usage:

| **Studies** | **Cao et al (2019)** | **Hao et al (2019)** | **The current systematic review** |
| --- | --- | --- | --- |
|  | NMA for vertigo control (a mixture of definitions, mostly class A and B, and no vertigo complaints).   - Only trials which compared intratympanic (IT) injection of medications were included | NMA for PTA change and frequency of vertigo attacks; the NMA for vertigo control (class A and B), FLS, SDS, and THI had no study between IT gentamicin and placebo.   - Trials of IT glucocorticoids and gentamicin, compared with each other or placebo were included | NMA for PTA change and complete vertigo control; meta-analyses or narrative summary were also presented for hearing endpoints, vertigo endpoints, aural fullness, tinnitus, FLS, DHI and other endpoints related to QoL / handicap / disability measures   - Trials of pharmacologic and surgical interventions |
| Bremer (2014) | Included in SR and NMA. | Included in SR but considered as not having provided any available outcome data (due to premature end of the trial without sufficient data). | Included in SR but excluded from analyses. This study had been terminated early with an unscheduled interim analysis because one patient exceeded the accepted amount of hearing loss and the enrollment was very low. There were differences in the baseline characteristics across groups. |
| Guyot (2008) | Included in SR and NMA. | Not included in SR: intervention (ganciclovir) was not of interest. | Not included in this SR: intervention (ganciclovir) was not of interest. |
| Rask-Andersen (2005) | Included in SR and NMA. | Not included in SR: intervention (latanoprost) was not of interest. | Not included in this SR: intervention (latanoprost) was not of interest. |
| Casani (2012) | Complete or substantial vertigo control (class A and B):  30/32 (IT gentamicin) vs 17/28 (IT dexamethasone). | Complete or substantial vertigo control (class A and B):  30/32 (IT gentamicin) vs 17/28 (IT dexamethasone).  Besides, changes in PTA and SDS were also used in analyses. It was unclear how the SDs of the mean changes were calculated. | Complete or substantial vertigo  control (class A and B):  30/32 (IT gentamicin) vs 17/28 (IT dexamethasone).  We also used its results for changes in PTA and SDS, calculated RR of patients with improved hearing >= 10 dB, and narratively summarized changes in FLS. |
| Garduño-Anaya (2005) | Complete or substantial vertigo control (class A and B): 11/11 (IT dexamethasone) vs 4/7 (placebo) at 24 months. In the placebo group, only seven patients who completed the follow-up were used, among which two had switched to treatments other than placebo. Four patients in the placebo group who dropped out ended up with class F (treatment abandoned for one patient, vestibular neurectomy for one patient, IT dexamethasone for two patients). | Complete or substantial vertigo control (class A and B): 9/11 (IT dexamethasone) vs 4/11 (placebo) at 24 months.  Besides, changes in PTA, SDS, FLS, and vertigo frequency were also used in analyses. | Complete vertigo control (class A): 6/11 (IT dexamethasone) vs 0/11 (placebo),  Complete or substantial vertigo control (class A and B): 9/11 (IT dexamethasone) vs 2/11 (placebo) at 6 months.  Treatments beyond the protocol were applied and patient dropout happened from 6 months to 24 months. Therefore, we only used data at 6 months for patients with vertigo control, relief from aural fullness, and improved hearing >= 10 dB, and changes from baseline to 6 months for PTA, SDS, FLS, DHI, and THI for comparison between groups. Mean change for vertigo frequency per arm (highly skewed and zero-inflated) was summarized. |
| Masoumi (2017) | 25/36 (IT dexamethasone) vs 24/33 (IT methylprednisolone): The level of vertigo control that correspond to numeric values of 0-40 (class A and B per AAO-HNS 1995 definition) and 41-80 (class C) were used. The classes labelled in the primary study were not based on AAO-HNS 1995 and might have misled the authors of the review. | Not included in SR:  Possibly because dexamethasone and methylprednisolone belong to the same treatment node (IT steroid), and therefore cannot be used for NMA. | Included only for pairwise meta-analysis.  6/36 (IT dexamethasone) vs 7/33 (IT methylprednisolone): We had used the number of patients with level of vertigo control that correspond to numeric values of 0-40 only, which was consistent with vertigo control class A (0: complete control) and B (1-40: substantial control) per AAO-HNS 1995 definition. We also calculated RR of patients with improved hearing >= 10 dB. |
| Patel (2016) | 19 patients in the IT gentamicin group, and 20 patients in the IT steroid group had no attacks of vertigo between 18 months and 24 months after the first injection. | Complete or substantial vertigo control (class A and B): (25+3)/30 (IT gentamicin) vs (21+3)/11 (IT steroid)  Besides, changes in PTA, SDS, THI, FLS, vertigo frequency were also used in analyses. It was unclear how the SDs of the mean changes were calculated. | 25 (complete control) and 3 (substantial control) for the IT gentamicin group,  21 (complete control) and 3 (substantial control) for the IT steroid group, according to Table S1, Patel et al (2016).  We also used its results for changes in PTA, SDS, DHI, THI, FLS, calculated RR of calculated RR of patients with improved hearing >= 10 dB, and narratively summarized severe adverse events, changes in vertigo frequency, vertigo severity (vertigo symptom scale score), and aural fullness analogue score. |
| Postema (2008) | The primary study reported 9 patients without vertigo complaints in the IT gentamicin group 1 year after treatment. Cao et al (2019) assumed 0 patients without vertigo complaints in the placebo group. | Only mean changes in the extended Fletcher index were used (in the analysis of PTA change). | We used 9 patients in the IT gentamicin group and 1 patient in the placebo group (with vertigo score 0) as not having any vertigo complaints 1 year after treatment, according to Figure 4 in Postema et al (2008).  We also used its results for PTA change, and narratively summarized changes in vertigo severity, tinnitus severity, and aural fullness severity score (ranged 0-3). |
| Stokroos and Kingma (2004) | In the gentamicin group, all 12 patients reported no complaints of vertiginous attacks 6 weeks after the last treatment and during follow-up. In the placebo group, one patient reported a significant reduction in the frequency of vertigo attacks (considered as no vertigo complaints by Cao et al 2019). | Number of patients with no vertigo complaints or significant reduction in vertigo attacks were not used.  Changes in PTA and vertigo frequency were used in analyses. | In meta-analysis but not in NMA:  For no vertigo complaints, we used 12 patients in the IT gentamicin group and 0 patients in the placebo group.  For no vertigo complaints or significant reduction in vertigo attacks, we used 12 patients in the IT gentamicin group and one patient in the placebo group.  We also used its results for PTA change (only for sensitivity analysis), and narratively summarized changes in vertigo frequency. |
| Adrion (2016) | Oral betahistine (vs placebo) was not of interest. | Oral betahistine (vs placebo) was not of interest. | It did not report vertigo control as an outcome. We used its results for changes in PTA, DHI, and VDADL.  We summarized changes in vertigo frequency, tinnitus intensity, MiniTF, as well as severe adverse events and withdrawals due to adverse events. |
| Albu (2015) | Not included in SR:  IT dexamethasone vs high-dose betahistine | Not included in SR:  IT dexamethasone vs high-dose betahistine | Complete vertigo control (class A):  14/30 vs 12/29.  Complete or substantial vertigo control (class A and B): 20/30 vs 17/29.  We also used its results for changes in PTA, SDS, DHI, THI, FLS, and calculated RR of patients with improved hearing >= 10 dB.  We narratively summarized its results for changes in vertigo frequency. |
| Albu (2016) | Not included in SR:  IT dexamethasone with and without high-dose betahistine | Not included in SR:  IT dexamethasone with and without high-dose betahistine | Complete vertigo control (class A):  14/30 vs 12/29.  Complete or substantial vertigo control (class A and B): 27/30 vs 21/32.  We also used its results for changes in PTA, SDS, THI, FLS, and calculated RR of patients with improved hearing >= 10 dB.  We narratively summarized its results for changes in vertigo frequency. |
| ElBeltagy (2012) | Not included in SR  (unclear why this trial which compared IT gentamicin and IT dexamethasone was not included) | Complete or substantial vertigo control (class A and B): 13/13 vs 13/13.  Besides, changes in PTA, SDS, vertigo frequency were also used in analyses. It was unclear how the SDs of the mean changes were calculated. | Complete vertigo control (class A):  10/13 vs 4/13.  Complete or substantial vertigo control (class A and B): 13/13 vs 13/13.  We also used its results for changes in PTA and SDS, and patients with relief from aural fullness. Changes in vertigo frequency, DHI (categories), and tinnitus symptom (categories) were narratively summarized. |
| Sarafraz (2015) | Not included in SR  (unclear why this trial which compared IT gentamicin and IT methylprednisolone was not included) | Complete or substantial vertigo control (class A and B): 7/10 (IT gentamicin) vs 5/10 (IT steroid) | Not included in this SR:  This study reported data at 3-month follow-up, while our protocol had specified that eligible trials must have minimum follow-up duration of 6 months |
| Ganança (2009) | Betahistine 16 mg tid (vs 24 mg bid) was not of interest. | Betahistine 16 mg tid (vs 24 mg  bid) was not of interest. | It did not report vertigo control as an outcome. We narratively summarized its changes in vertigo frequency. |
| Morales-Luckie (2005) | Oral prednisolone + maintenance therapy (diphenidol + acetazolamide + low-sodium diet < 1,500 mg/d) and maintenance therapy alone were not of interest. | Oral prednisolone + maintenance therapy (diphenidol + acetazolamide + low-sodium diet < 1,500 mg/d) and maintenance therapy alone were not of interest. | Complete vertigo control (class A): 0/8 vs 0/8.  Complete or substantial vertigo control (class A and B): 7/8 vs 0/8.  We narratively summarized its aural fullness, withdrawals due to adverse events, changes in hearing, vertigo frequency, tinnitus symptom, and self-assessed functional disability. |
| Paragache (2005) | Not included in SR:  IT dexamethasone vs betahistine 48 mg/d + cinnarizine 75 mg/d + diet restrictions | Not included in SR:  IT dexamethasone vs betahistine 48 mg/d + cinnarizine 75 mg/d + diet restrictions | Sakata’s criteria (possibly excellent and good control of vertigo) had been used:  9/20 (IT dexamethasone) vs 8/20 (betahistine 48 mg/d + cinnarizine 75 mg/d + diet restrictions)  We also calculated RR of patients with relief from aural fullness and patients with improved hearing >= 10 dB, and narratively summarized the changes in SDS and tinnitus symptom. |
| Bojrab II (2018) | Endolymphatic sac decompression (ESD) surgery with and without steroid injection were not of interest. | Endolymphatic sac decompression (ESD) surgery with and without steroid injection were not of interest. | It did not report vertigo control as an outcome. We narratively summarized its results for changes in PTA, vertigo frequency, DHI, THI, and MDOQ, and patients with improved hearing. (The number of patients per group beyond baseline was unclear.) |
| Kitahara (2016) | Tympanic ventilation + oral medications (vs oral medications alone) was not of interest. | Tympanic ventilation + oral medications (vs oral medications alone) was not of interest. | Complete vertigo control (class A): 53/63 (tympanic ventilation + oral medications) vs 38/70 (oral medications alone).  We calculated RR of patients with improved hearing >= 10 dB, and narratively summarized its results for Self-rating Depression Scale and Stress Response Scale-18. |
| Saliba (2015) | Endolymphatic duct blockage (EDB) and endolymphatic sac decompression (ESD) were not of interest. | Endolymphatic duct blockage (EDB) and endolymphatic sac decompression (ESD) were not of interest. | It did not report vertigo control as an outcome. We narratively summarized its aural fullness, tinnitus persistence, and changes in PTA, SDS, and vertigo frequency. (The number of patients per group beyond baseline was unclear.) |
| Lambert (2016) | Exclusion of this study at which step was not clearly stated.  Not included in analyses. | Included in SR but considered as not having provided any available outcome data (due to not having reported data in appropriate forms) | Not included in this SR:  This study reported results up to 4-month follow-up, while our protocol had specified that eligible trials must have minimum follow-up duration of 6 months |
| Lambert (2012) | Exclusion of this study at which step was not clearly stated.  Not included in analyses. | Included in SR, and changes in THI were included in analyses. | Not included in this SR:  This study reported results up to 3-month follow-up, while our protocol had specified that eligible trials must have minimum follow-up duration of 6 months |

Extra references only for this Appendix:

Guyot JP, Maire R, Delaspre O. Intratympanic application of an antiviral agent for the treatment of Meniere's disease. *ORL J Otorhinolaryngol Relat Spec.* 2008; 70(1): 21-26.

Rask-Andersen H, Friberg U, Johansson M, Stjernschantz J. Effects of intratympanic injection of latanoprost in Meniere's disease: a randomized, placebo-controlled, double-blind, pilot study. *Otolaryngol Head Neck Surg* 2005;133(3):441-443.

Sarafraz M, Saki N, Nikakhlagh S, Mashali L, Arad A. Comparison the efficacy of intratympanic injections of methylprednisolone and gentamicin to control vertigo in unilateral Meniere’s disease. *Biomed Pharmacol J* 2015; 8 (October Spl Edition): 705-709.

Lambert PR, Carey J, Mikulec AA, LeBel C, Otonomy Ménièreʼs Study Group. Intratympanic sustained-exposure dexamethasone thermosensitive gel for symptoms of Ménière's Disease: Randomized phase 2b safety and efficacy trial. *Otol Neurotol* 2016; 37(10): 1669-1676.

Lambert PR, Nguyen S, Maxwell KS, Tucci DL, Lustig LR, Fletcher M, Bear M, Lebel C. A randomized, double-blind, placebo-controlled clinical study to assess safety and clinical activity of OTO-104 given as a single intratympanic injection in patients with unilateral Meniere's disease. *Otol Neurotol* 2012 ;33(7): 1257-1265.
